# Boolean Calcium Signalling Model Predicts Calcium Role in Acceleration and Stability of Abscisic Acid-Mediated Stomatal Closure

**DOI:** 10.1038/s41598-018-35872-9

**Published:** 2018-12-05

**Authors:** Pramuditha Waidyarathne, Sandhya Samarasinghe

**Affiliations:** 1 Complex Systems, Big Data and Informatics Initiative (CSBII), Lincoln University, Christchurch, New Zealand; 2 Coconout Research Institute, Lunuwila, Sri Lanka

## Abstract

Inconsistent hypotheses have proposed Ca^2+^ as either being essential or irrelevant and redundant in ABA induced stomatal closure. This study integrates all available information from literature to define ABA signalling pathway and presents it in a systems view for clearer understanding of the role of Ca^2+^ in stomatal closure. Importantly, it incorporates into an Asynchronous Boolean model time delays sourced from an extensive literature search. The model predicted the timing of ABA events and mutant behaviour close to biology. It revealed biologically reported timing for Ca^2+^ activation and Ca^2+^ dynamics consistent with biology. It also predicts that Ca^2+^ elevation is not essential in stomatal closure but it can accelerate closure, consistent with previous findings, but our model further explains that acting as a mediator, Ca^2+^ accelerates stomatal closure by enhancing plasma membrane slowly activating anion channel SLAC1 and actin rearrangement. It shows statistical significance of Ca^2+^ induced acceleration of closure and that of Ca^2+^ induced acceleration of SLAC1 activation. Further, the model demonstrates that Ca^2+^ enhances resilience of closure to perturbation of important elements; especially, ROS pathway, as did previous ABA model, and even to the ABA signal disruption. It goes further to elucidate the mechanisms by which Ca^2+^ engenders stomatal closure in these perturbations.

## Introduction

Plants have evolved a well-developed mechanism to quickly respond to droughts to sustain their life under extremes of water stress. The production of the phytohormone abscisic acid (ABA) in response to drought invokes a series of cell signalling pathways causing rapid stomatal closure in plants to prevent water loss and cell dehydration^1^. Calcium (Ca^2+^), the most ubiquitous second messenger in plant cells, is tightly connected with all the important functions of ABA signalling (Osmoregulation, Structural rearrangement, ROS (reactive oxygen species) and lipid) (Supplementary Fig. S1 with details to follow) thus appearing to be a central hub in the main system^2^. Coordinated activity of guard cell Ca^2+^ channels, pumps and transporters generate Ca^2+^ signals that initially appear as random Ca^2+^ elevations; these later develop into temporally defined oscillations^3^.

According to experimental literature, the role of Ca^2+^ in ABA signalling is not yet fully established. Some studies have shown that Ca^2+^ is fundamental to stomatal closure^4^. Other studies have shown slower stomatal closure in response to conditions that prevented Ca^2+^ elevation in guard cell cytoplasm^5^. Some other studies, however, have reported that Ca^2+^ elevation is not needed for stomatal closure^6^ by revealing uninterrupted downstream events, such as ion channel regulation^7,8^ (which can be regulated by Ca^2+^ as well), even after inhibition of Ca^2+^ signalling in guard cells. Further, some studies have suggested that there may be two transduction routes in ABA induced stomatal closure (Ca^2+^ dependent and independent) where activation of either pathway is sufficient to induce stomatal closure^9^. More recently another group of scientists hypothesized that certain threshold level of Ca^2+^ elevation regulates stomatal closure and a defined pattern of Ca^2+^ oscillation is needed to inhibit stomatal reopening, or maintain closure^3^. Supporting this latter hypothesis on closure maintenance, some research has reported that ABA-induced steady state stomatal closure is characterised by a series of Ca^2+^ oscillations with defined frequency and amplitude^3^. Together, these hypotheses raise a fundamental question about the functional significance of Ca^2+^ within the complex ABA signalling in plant guard cells.

Despite the progress in our understanding of the transmission of Ca^2+^ signals within the ABA signalling system in the past years, the regulatory principles that convert cellular Ca^2+^ signals into guard cell responses remain unexplored^10^. Engineering the guard cell signal transduction network could therefore make a major contribution to solving the mystery of Ca^2+^ signalling in guard cells. There have been few modelling attempts to solve this mystery due to the lack of knowledge of biological connectivity between the players of the large ABA network and extreme lack of data. The last modelling attempt of ABA signalling prior to our study, a random order asynchronous Boolean model with 43 proteins and 76 interactions, was reported in 2006^11^. Their model identified the important elements of the ABA network, but the reproducibility of realistic temporal events was limited due to the assumed randomness in network updating order as the model did not incorporate biological time delays associated with the reactions. However, authors concluded that stomatal closure is accelerated when the Ca^2+^ level is elevated but ABA signalling can achieve the end target even without Ca^2+^ in the system. In supporting their conclusions, they make the prediction that their model predictions will stay the same as the co-topological properties of the network stay the same even with future network modifications. Nevertheless, they reported on the essentiality of Ca^2+^ to the system under some important nodal disruptions^11^. However, later in 2010, the same authors^12^ acknowledged the significant dependence of the system behaviour on the manner of update indicating that incorporation of a biologically valid updating scheme (timing) may produce biologically realistic behaviour. Therefore, the following major additions different from the above mentioned previous ABA signalling network model^11^ were introduced to the existing ABA signalling network to better study the system behaviour. First, we incorporated a biologically valid updating scheme (timing) into the model. Second, the network obtained is significantly more comprehensive with the incorporation of a number of important novel experimental findings.

**Figure 1:**
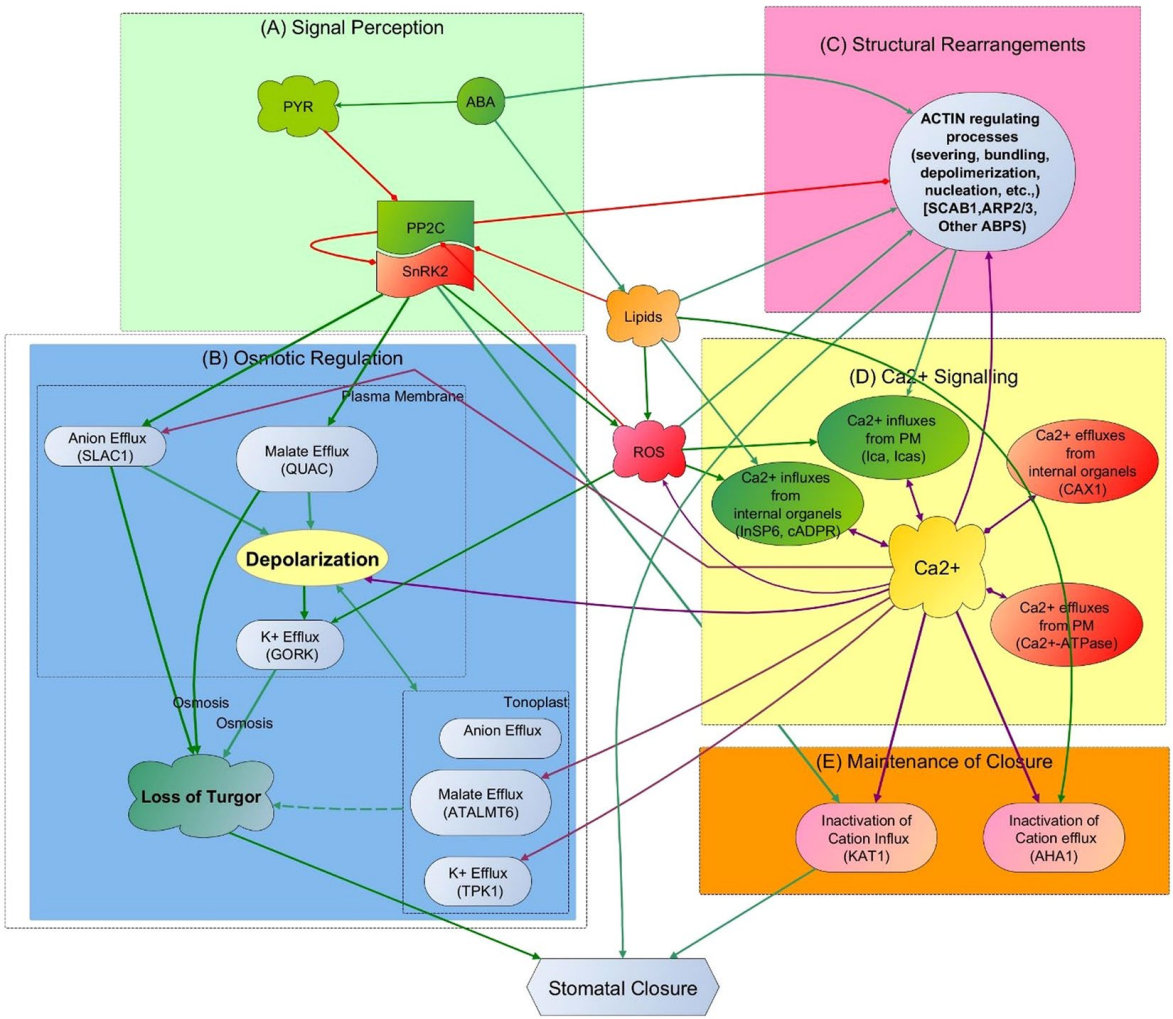
High level view of functional flow of the ABA signalling network with interacting functional subsets of: (**A**) signal Perception, (**B**) Osmotic Regulation, (**C**) Structural Rearrangement, (**D**) Ca^2+^ Signalling (all interactions for Ca^2+^ are shown here in purple to highlight the extent of involvement of Ca^2+^ in the system) and (**E**) Maintenance of Closure. (Pointed arrows show positive regulations and diamond-head arrows show negative regulations. In all subsets except Ca^2+^ signalling, green and red colour in arrows further highlight positive and negative regulations, respectively) (detailed description of each functional set is given in the supplementary text accompanied by Fig. S1)

To reveal the role of Ca^2+^ in stomatal closure, we first created the most complete ABA signalling network at the time of this study (56 nodes and 127 interactions) based on new information discovered since 2006 (Fig. S1- This extended network contains important new elements, interactions and functions that help better explain the organisation and intricate coordination of regulatory events involved in stomatal closure as highlighted in the next section). Importantly, we then transform the network into a meaningful systems view with a functional (temporal) hierarchy for gaining a higher-level functional view and clearer insights into the process of stomatal closure (Fig. 1). Then we introduce several methodological advancements based on asynchronous Boolean analysis to the extended ABA network to study its dynamic behaviour incorporating biologically realistic time delays (Table S1 and Methodology Section). We extracted these time delays from an extensive survey of fragmentary experimental literature (Table S1). This qualitative modelling framework allows us to probe into the role of Ca^2+^ with greater clarity and certainty than before. It is also the most promising approach at present in the absence of proteomics time series data and the required kinetic parameters for continuous dynamics modelling of drought induced ABA signalling network.

## ABA Signalling and Ca^2+^

We carefully studied all the elements and interactions as well as the logic of operation of the extended ABA signalling pathway shown in Fig. S1 from the time of receiving ABA signal (top green node) to the end goal of stomatal closure (brown node at the bottom). This helped us understand the operation of the whole system and the role of Ca^2+^ in context as well as provide a systems view of ABA signalling. In broad terms, ABA induced stomatal closure involves the reduction in guard cell volume by means of two major functional events- osmotic regulations and structural rearrangements of the cell. Osmotic regulations are made to reduce cell turgor by releasing water out. Guard cell structural rearrangements play an important concurrent role here by providing the required flexibility for cell shrinkage during stomatal closure. Figure S1 shows the detailed network with all the elements and interactions that support these two functional events and the text accompanying this figure provides a description of each interaction. Such large networks are complex and make it difficult to understand functional subsystems within it. Based on our current knowledge of ABA signalling, we divided the network into a smaller number of meaningful subsystems and their interactions to better reveal the coordination of these two events. Specifically, upon receiving the ABA signal, the receptor complex (light green box in Fig. S1) initiates a series of signalling events to coordinate in parallel osmoregulation (purple box in Fig. S1), structural (actin) rearrangement (blue box) as well as Ca^2+^ signalling (yellow box) - elucidating the proper role of the latter is the aim of this study. Two other signalling subsystems, coordinated by the ABA signal, facilitate the above two main functions and these are ROS signalling (pink box) and Lipid Signalling (grey box).

Ca^2+^ signalling regulates the level of Ca^2+^ and connects Ca^2+^ with osmoregulation, actin rearrangement, lipid and ROS pathways (bicolour nodes in Fig. S1). A number of important novel additions make our model distinguishable from the previous Li *et al*.^11^ network. For example, our extended network highlights the role of the newly discovered^13,14^ ABA receptor complex and the regulatory connections that combine it with the downstream effectors of the ABA signalling system, which was not defined in the previous model. Further, our network expands the components of cellular structural rearrangement and their connections with other network elements to better explain the concurrent coordination of signal transduction between guard cell turgor reduction and structural rearrangement. In addition, regulatory components that are newly added to the parts regulating guard cell turgor reduction - Ca^2+^ signaling and various other signaling events (e.g., ROS) - have substantially differentiated this extended network topology from the previous Li *et al*.^11^ network. The upstream components of the Ca^2+^ regulatory pathway are nearly similar in both models but the definition of the Ca^2+^ dependent downstream regulations is considerably different in the two models. For example, Ca^2+^ release by the internal organelles is regulated by four independent regulators (Inositol trisphosphate (InSP3), Inositol hexaphosphate (InSP6), Cyclic guanosine monophosphate (cGMP) and Cyclic adenosine diphosphoribose (cADPR)) in the Li *et al*.^11^ model but we assume that these four regulators are not independent (InSP6 is the phosphorylated product of InSP3^15^ and cGMP enhances the production of cADPR^16^); therefore, our model considers them as two regulatory paths. Further, our model considers the contribution of structural rearrangements to cytosolic Ca^2+^ increase through stretch activated Ca^2+^ channels in the plasma membrane, which was not defined in the previous model. Moreover, the previous model considered the Ca^2+^ efflux system as a single node regulated by Ca^2+^. We are certain that the Ca^2+^ efflux system consists of two effluxes (Ca^2+^-ATPase^17^ and H^+^/Ca^2+^ exchangers (CAX)^18^) that are differently regulated by their respective regulators. Therefore, our model considers individual regulations of the two Ca^2+^ effluxes assuming that the addition of all known regulators will produce biologically evidenced Ca^2+^ signature with our asynchronous Boolean model incorporating real timing. Further, several novel additions, such as Ca^2+^ involvement in slowly activating anion channel (SLAC1) regulation and ROS production via Ca^2+^ dependent protein kinases (CDPK), and Ca^2+^ regulation of Ca^2+^-dependent vacuolar malate channel, ATALMT6, as well as actin binding proteins, to our model make it considerably different from Li *et al*.^11^ model.

Figure 1 captures the high-level systems view of ABA signalling from the details in Fig. S1 to demonstrate the main events and their functional/temporal hierarchy in stomatal closure and how intimately they are linked to Ca^2+^. Overall, stomatal closure is accompanied by several hierarchical functional events as indicated in the figure: Signal Perception, Osmotic Regulation, Structural Rearrangements, Ca^2+^ Signalling and Maintenance of Closure. In brief, ABA recognition by its receptor complex activates the SNF-related serine/threonine protein kinase (SnRK2) (functional set (A) in Fig. 1) facilitating the activation of downstream targets of the network to reduce cell turgor (stiffness) through a balanced and timely regulation (opening and closing) of ion channel activities to release water from the cell (functional set (B) in Fig. 1). This process of turgor reduction involves a series of steps to pump water out by regulating osmotic load inside the cell, achieved predominantly through efflux of potassium (via GORK (Plasma membrane potassium efflux) channel) and chloride ions (via SLAC1) and removal of organic acid Malate (via ATALMT6) and/or metabolising Malate into osmotically inactive starch. (Molecules involved in turgor reduction are various types of plant lipids, protein kinases, protein phosphatases, ion channel proteins, reactive molecules such as ROS, cytosolic pH and cytosolic Ca^2+^ (Fig. S1)). A variety of actin binding proteins that concurrently disassemble the radially arranged actin filaments in the open stomata into a random orientation facilitate guard cell shrinkage by providing the required relaxed cytoskeleton for stomatal closure (functional set (C) in Fig. 1). The ABA signalling network coordinates both osmoregulation and structural rearrangements as well as Ca^2+^ signalling (functional set (D) (Fig. 1)) in parallel. This ultimately leads to stomatal closure followed by evoking the regulatory mechanisms needed to keep stomata closed (functional set (E) in Fig. 1) until the plant becomes stress free. As shown, all the above functions are intimately connected to the Ca^2+^ signalling system. (Fig. S1 and accompanying text provide details of all the elements and interactions in these functional sets).

**Figure 2:**
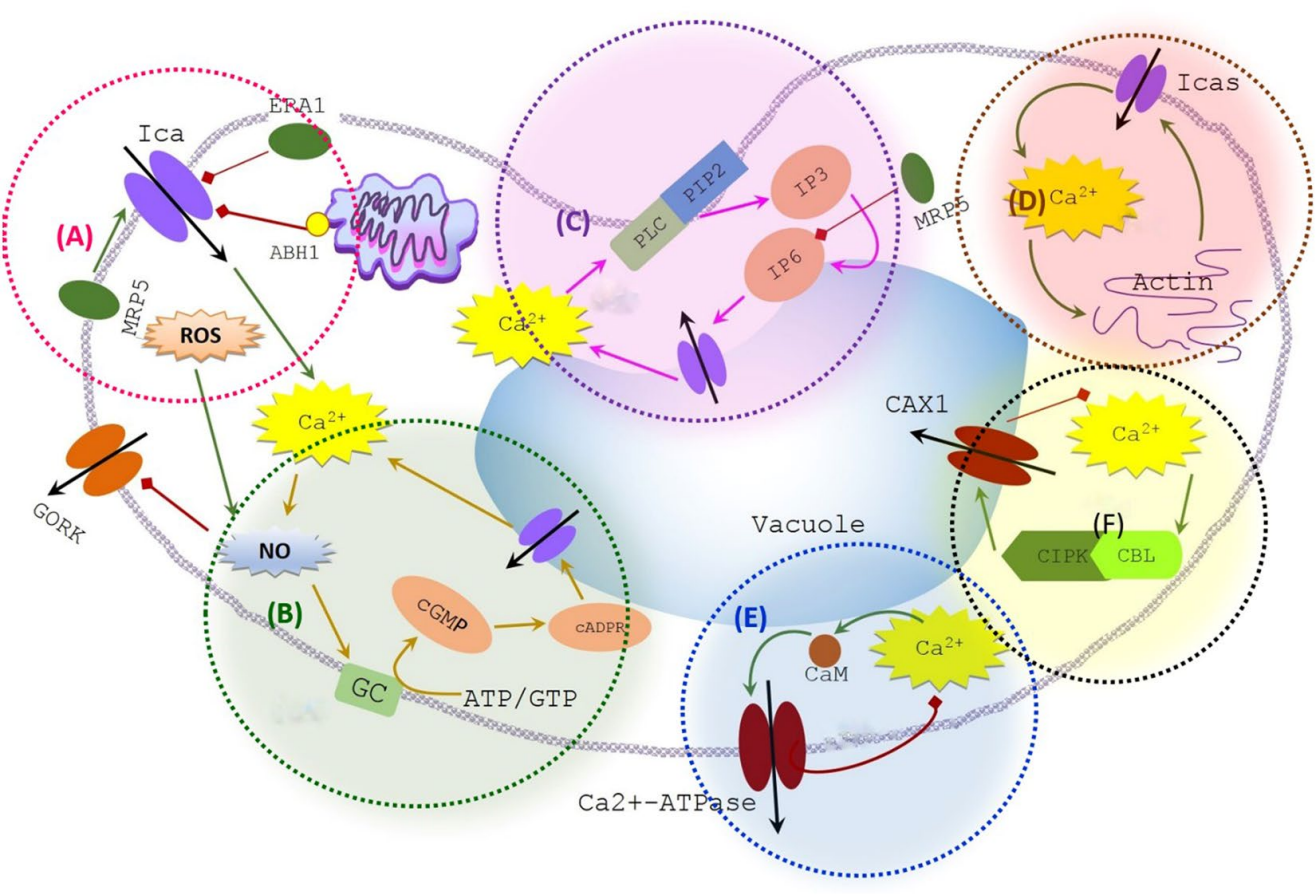
Calcium regulatory signalling in ABA signalling network. Shaded areas (superimposed on an outline of a cell) depict regulatory feedback loops of the system where: (**A**) Voltage-dependent plasma membrane calcium channel (Ica); (**B**) positive feedback between Ca^2+^  ↔ NO; (**C**) positive feedback between Ca^2+^  ↔ PLC; (**D**) positive feedback between Ca^2+^  ↔ actin; (**E**) negative feedback between Ca^2+^  ↔ Ca^2+-^ATPase; and (**F**) negative feedback between Ca^2+^  ↔ CAX1. For clarity, only vacuole is shown in the figure representing all internal organelles of the guard cell (Regular and diamond head arrows represent positive and negative interactions, respectively) (Ica = Hyperpolarization activated plasma membrane Ca^2+^ channel, ERA1 = farnesyltransferase subunit beta, MRP5 = ABC transporter, ABH1 = mRNA cap binding protein subunit 1, NO = Nitric Oxide, GC = Guanosine cyclase, PLC = Phospho Lipase C, PIP2 = Phosphatidylinositol 4,5-bisphosphate, IP3 = InSP3, IP6 = InSP6, Icas = Stretch activated plasma membrane Ca^2+^ channel, CIPK = CBL interacting protein kinase, CBL = Calcineurin B-like calcium-binding protein, CaM = Calmodulin).

Ca^2+^ that constitutes the most connected functional set in ABA signalling (yellow box and purple arrows in Fig. 1) is not an osmotically active cation in plant cells. Maintenance of cytosolic Ca^2+^ at a low micro-molar level (0.05–0.3 µM^19^) is an evolutionary constraint in all cell types. This is because Ca^2+^ can be toxic at higher concentrations as higher levels of free cytosolic Ca^2+^ bind with orthophosphates present in ATP causing precipitation of low soluble calcium salts in the cytoplasm^20^; this alters the biological reactions demanding free energy transduction. To maintain Ca^2+^ at a level safe to cellular functions, guard cells employ a well-developed signalling network (Fig. 2), which mainly comprises four Ca^2+^ influx systems and two Ca^2+^ efflux systems. Of the four different Ca^2+^ influxes, the initial contribution is from the voltage-dependent plasma membrane calcium channel (Ica) (Fig. 2(A) – circled with pink border), which transiently provides Ca^2+^ currents to the cytoplasm^21^. In addition, there are two positive feedback loops to enhance the cytosolic Ca^2+^ concentration by pumping Ca^2+^ out from the internal organelles. The first feedback loop is through nitric oxide (NO) ↔ Ca^2+^ (Fig. 2(B) – circled with green border) and the other is through phospholipase C (PLC) ↔ Ca^2+^ (Fig. 2(C) – with purple border). Further, there are reports to indicate the contribution of mechanosensitive Ca^2+^ channels in the plasma membrane to the influx system at a later stage, as a result of actin filament rearrangements^22^ (Fig. 2(D) – with brown border). Removal of Ca^2+^ from the cytoplasm is either through Ca^2+^-ATPases (Fig. 2(E) – with blue border) or CAX1 activity (Fig. 2(F) – with black border).

The coordinated activity of these regulators through positive/negative feedback loops in a timely and functionally-dependent manner generates the required Ca^2+^ signals in the guard cell cytosol. Generation of Ca^2+^ spikes^3^ in a tightly controlled environment strongly indicates that Ca^2+^ is a crucial element in the ABA signalling network. However, the significance of Ca^2+^ signalling to ABA signalling is yet to be confirmed.

## Temporal dynamics of Ca^2+^ in ABA signalling

We simulated ABA signalling network dynamics with Asynchronous Boolean approach by incorporating realistic time delays into a Boolean framework (see Materials and Methods). We collected fragmentary timing data for each interaction from an extensive search through various sources and fitted them into the ABA time scale while minimizing discrepancies. This was considered the best and most realistic option for incorporating timing into the model due to the lack of availability of a complete set of proteomics time series data to order the correct sequence of update in the ABA system. Under this condition, we constructed different but biologically realistic time delays for the 127 interactions in the ABA network.

We assumed that physical bindings, posttranslational modifications of proteins, ion channel activities, membrane charge alterations and other interactions determine the dynamics of the system. The key role here is played by the associated time delays as they correspond to the actual reaction speeds; for example, activation of a specific target at lower expression level is represented by lower time delay and vice versa. This approach incorporating realistic time delays is the first exploratory step to understand problems which are too complex to model using partial differential equations or other continuous approaches. In this model, the output of a Boolean function representing a reaction is the combined effect of the inputs and their associated time delays, bringing it one step closer to continuous dynamics. The value of this approach is further boosted by the current perception that the actual timing could be more important than the exact levels of proteins. (In fact, the closeness of timing of many model events to the actual ABA events indicates that the timing used in the study is reasonable (Supplementary Table S2)). The model was further validated comparing biological mutants with model mutants (Details are shown in Supplementary Table S3 as they are not relevant to the objectives of this paper). Here, we only discuss the temporal behaviour of Ca^2+^ signalling as our aim is to question the existing hypotheses on Ca^2+^ signals in ABA signalling.

We observed throughout 10,000 simulations with different initial conditions that cytosolic Ca^2+^ first appears as random elevations during stomatal closure process and later, after the onset of closure, it develops into regular oscillations with equal magnitude as observed in the previous Boolean model^11^ and in experimental studies^3,23^. Our model in particular identified that the initiation of Ca^2+^ elevations in guard cell cytoplasm occurred, 1–12.5 min after receiving the drought signal, ABA, agreeing with the biological evidence in the literature^23,24^. This is considerably variable compared to the timing of other functional events in the system and the 15 min time frame for stomatal closure. This large variation may be because Ca^2+^ does not have a must do role within this system; otherwise, Ca^2+^ elevations should occur within a less variable time frame to achieve the system outcome, stomatal closure, by 15 min.

**Figure 3:**
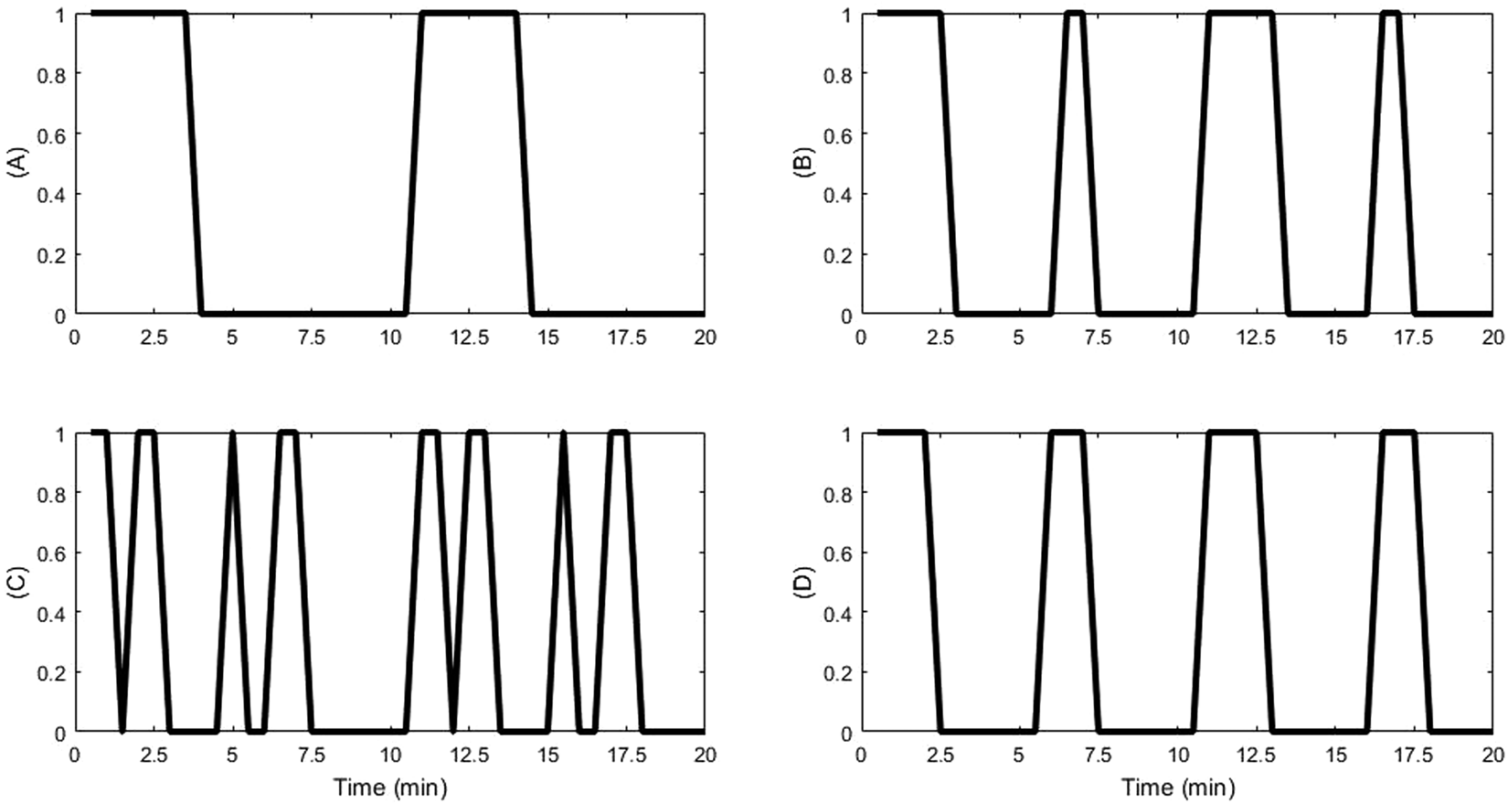
Pattern of Ca^2+^ oscillations in the four most common attractors found in the asynchronous system.

**Figure 4:**
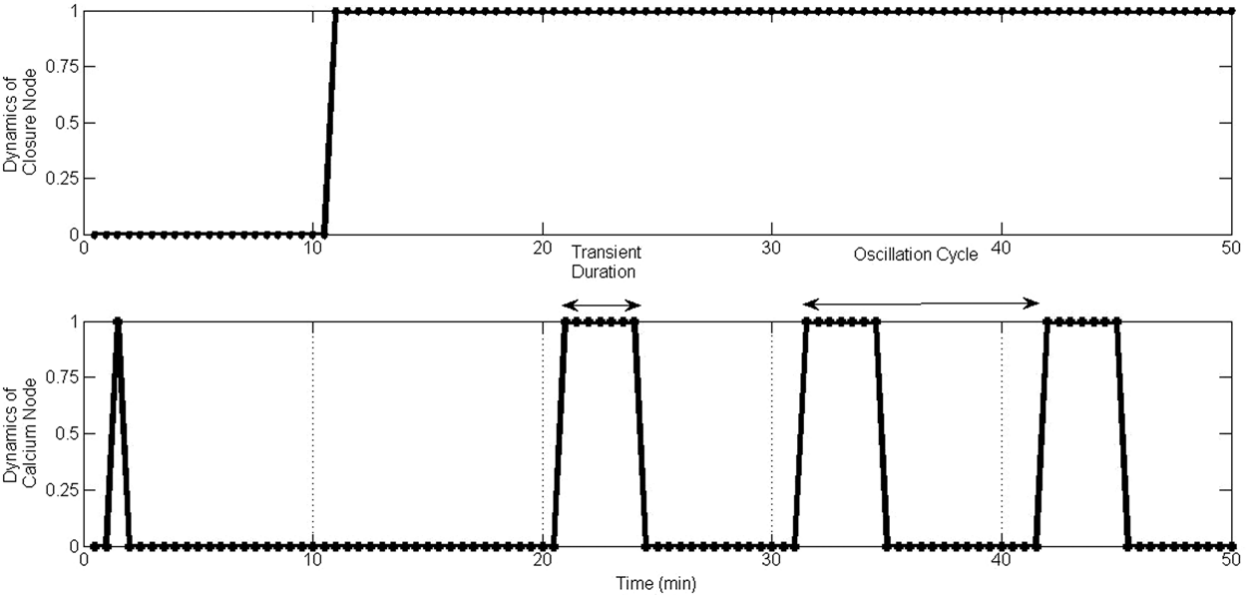
Oscillatory behaviour of Ca^2+^ in the most frequent attractor of the ABA signalling system.

Our model further showed that different initial conditions of the system cause behavioural differences in rhythmic Ca^2+^ oscillations (Fig. 3 shows Ca^2+^ oscillations of the four most common attractors) resulting in different steady state behaviour in the ABA signalling system. Some of the observed patterns are spurious as a result of assumed initial conditions for some nodes due to the lack of knowledge of biologically possible initial protein activity levels as well as currently unknown regulatory mechanisms for some nodes (e.g., MRP5, ERA1 and ABH1 (see Fig. 2)). Due to the real timing used in the model, it was observed that of the many observed patterns (>80), the most prominent steady state (~24% of the total) behaviour showed 3.5 min of Ca^2+^ transients with a periodicity of once every 10 min as reported in the experimental literature^3^ (Figs 3(A) and Fig. 4). The previous Li *et al*.^11^ model found Ca^2+^ oscillations decaying and suggested that strict timing pattern where positive feedback loops of Ca^2+^ is longer in time than negative feedback loops is required to sustain oscillations. In our model with real timing, Ca^2+^ oscillations were sustained without decay; and the positive feedback loops turned out to be longer than the negative loops. Specifically, there are four positive feedback loops with 4, 4, 6 and 24 model time units and two negative feedback loops with 3 and 9 model time units. Regardless of the different initial conditions, most nodes (42) in the system reach a fixed state with the exception of few nodes (14) that include Ca^2+^ and Ca^2+^ governed molecules that form a limit cycle attractor. Stationary nodes which were shared by both models (ours and Li *et al*.^11^) showed similar steady state levels but the dynamic nodes showed non-identical oscillatory patterns. (We analysed our network with synchronous Boolean approach as well and observed that the status of the frozen nodes stays the same regardless of the updating method, but the observed limit cycles differ considerably due to the different behavioural patterns of the dynamic nodes).

Observing temporal dynamics of Ca^2+^throughout the simulation, we conclude that the system converges into these rhythmic oscillations after the onset of closure. This raises the question as to why the rhythmic oscillations are needed after the onset of closure. Does the system require Ca^2+^ oscillations to maintain the steady state of stomatal closure? Biologically, if the ABA system does not need any signalling communication after the onset of closure, the steady state dynamics of the system should be a fixed-point attractor. If the ABA system can close stomata with no Ca^2+^ oscillations and if it can maintain closure with the same steady level activities of the other network elements, then it does not need Ca^2+^ oscillations to appear in the second phase. If any signalling communication occurs after the onset of closure, *i*.*e*. oscillatory behaviour, it should be assigned some important role during the steady state.

The observation that the Ca^2+^ oscillations appear after stomatal closure supports one of the existing hypotheses that there are two phases in ABA induced stomatal closure. The first phase induces closure (rapid phase) and the second phase maintains closure by inhibiting the reopening of the stomata. This hypothesis was supported by experimental literature which alludes to: “…short term ‘calcium-reactive’ closure that occurred rapidly when cytosolic Ca^2+^ was elevated, and ‘calcium programmed’ long-term steady-state closure that occurred with Ca^2+^ oscillations with a defined range of frequency, duration and amplitude”^3^. Accordingly, the role of cytosolic Ca^2+^ oscillations may be for the second phase (maintenance of closure) to keep the stomata closed without reopening because stomata are liable to open during daytime in response to favourable abiotic signals, such as blue light. However, our current model has not specifically incorporated the relevant pathways that control the inhibition of stomatal reopening to evaluate it further. This is because cell signalling mechanisms for stomatal reopening is another complex signalling system, which comprises nearly 70 molecules connected by 150 interactions governed by four signals (blue light, red light, CO_2_ and ABA). This was recently modelled using a multi-level dynamic model^25^ showing that there is Ca^2+^ regulated robust cross-talk between blue light and ABA. Moreover, a recent study done by Minguet-Parramona *et al*.^26^ (a model on guard cell ion transport) reported that rhythmic oscillations of Ca^2+^ are a by-product of ion channel activities in guard cells, specifically AHA1, an active H^+^ transporter on the plasma membrane that attempts to open stomata^27^. Further, experimental evidence also suggests that regular Ca^2+^ oscillations may be more important for the maintenance of closure than for the induction of closure^3,23^. Therefore, in this paper we emphasize the potential roles of Ca^2+^ elevations in short term closure (Ca^2+^-reactive).

## Role of Ca^2+^ in rapid stomatal closure

**Figure 5:**
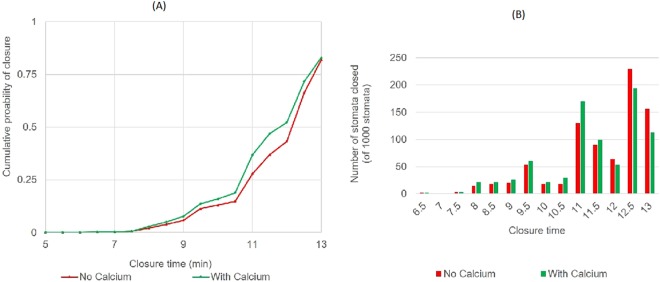
Acceleration of stomatal closure by Ca^2+^ elevation: (**A**) Cumulative probability of closure achieved with time; (**B**) number of stomata closed at each time step.

As Fig. 1 shows Ca^2+^ appears as a hub element in the system, communicating with almost all the other functional sets. Hub components in a scale-free network (ABA signalling network is hypothesized to have scale-free topology^28^) are extremely important and therefore have the potential to disturb the functional stability of the network. Importance of Ca^2+^ elevation prior to the system reaching steady state dynamics was, therefore, evaluated by the probability of stomatal closure (Eq. 4 in Methods Section) in the wild type (WT) and Ca^2+^ regulation knocked out systems. Here we hypothesised that Ca^2+^ may enhance the probability of stomatal closure under a variety of environmental conditions. Therefore, probability of closure in two ABA signalling systems, one with Ca^2+^ signalling pathway intact and the other with no Ca^2+^ regulation, was compared in response to a given set of initial conditions (1,000 here). The initial conditions used in the simulation were identical in both systems. The model results revealed that the presence of Ca^2+^ has no effect on the probability of closure, where the probability (~80%) remains the same in both Ca^2+^ dependent and independent systems (Fig. 5(A)). According to our model, closure probability is less than 100% due to the lack of knowledge of regulation of the three previously mentioned elements (MRP5, ERA1 and ABH1) and unknown initial conditions of many nodes (>50%) in the model. The model may achieve 100% closure when the regulatory mechanisms of these elements are revealed and all initial conditions are known. However, biological experiments also report that there are ABA unresponsive stomata as well as the difficulty in distinguishing open and closure states for some stomata leading to less than 100% stomatal closure. The model predicted that some of the model stomata do not produce Ca^2+^ elevations before the system reaches steady state which is also supported by literature^4,18^. Further, literature supports the fact that inhibition of Ca^2+^ elevation in guard cells has no effect on the occurrence of downstream events that are also connected to Ca^2+^, such as ion channel regulation^7,8^. Therefore, our results support the argument that stomata can be closed with no Ca^2+^ elevation.

At present, there are two opposing arguments about the essentiality of Ca^2+^ to guard cell signalling pathway based on SLAC1 activation. First, several studies^29–31^ conducted in oocytes expression system claimed that Ca^2+^-independent protein kinase SnRK2 (OST1) activates SLAC1 independently of Ca^2+^. To date, no evidence confirms that activation of SLAC1 in planta is solely through phosphorylation by OST1 as in oocytes. The second hypothesis claims that there is no Ca^2+^ independent SLAC1 regulation pathway in guard cells. Different research groups supporting the second hypothesis have shown that background level Ca^2+^ was required for ABA induced SLAC1 anion channel function using planta data^8,32–34^. One study, which claimed the second hypothesis showed that anion currents were observed even after blocking the Ca^2+^ entry into the cytoplasm but buffering Ca^2+^ below physiological level abolished ABA activation of anion channels^8^. In this study, we assumed that guard cell SLAC1 channel can be regulated independently by either OST1 (without elevating Ca^2+^) or Ca^2+^ dependent pathways via CPKs. Ca^2+^ elevation independent OST1 activation of SLAC1 was evidenced in oocyte expression system with plant SLAC1 and OST1 proteins^30,31^, hence, it may exist in plant guard cells as well. This and many other observations point to the possibility of Ca^2+^ independent SLAC1 activation by OST1 but some inconsistent counter evidence exists as explained below.

A recent study^33^ supported Ca^2+^ dependence of the system by showing altered stomatal sensitivity of quadruple mutants (cpk5/6/11/23) of Ca^2+^ dependent protein kinases (CPKs) to ABA. Two more studies from the same group^32,35^ reported that CPK6 and CPK23 display a prominent calcium-independent SLAC1 phosphorylation activity similar to OST1. It was shown that CPK23 is active at resting cytosolic Ca^2+^ concentrations^32^, but cpk23 mutant showed no stoma phenotype, which was discussed as the functional redundancy with other CPKs. Further, it was suggested in the experimental literature that CPK23 may act as a negative regulator of stomatal closure in response to drought that activates ABA signalling in plant guard cells as cpk23 mutant displayed greatly reduced stomatal apertures^36^, while overexpression of CPK23 increased stomatal apertures. Moreover, another study^29^ showed that CPK6 and OST1 kinases strongly and independently activate SLAC1 in oocytes but another study^37^ showed that loss of function of CPK3 and CPK6 (double mutants) substantially reduced SLAC1 activity and ABA induced stomatal closure in plants. Another recent study done on plants showed that quadruple mutants of cpk3/6/5/11 did not strongly impair ABA induced stomatal closure^38^ indicating the existence of several regulatory mechanisms for SLAC1. These experimental findings are not consistent; hence, further analyses are required to understand biological mechanisms behind the role of CPKs. Sometimes, an adequate amount of OST1 may not be released into the cytoplasm at low ABA levels, and hence help of CPK may be needed for full activation of SLAC1 to close the stomata. At higher levels of ABA where there may not be such limitation of OST1 in the guard cell cytoplasm, OST1 may fully activate SLAC1 where CPK can further accelerate the activity of SLAC1 by stimulating peak activity or inhibiting slow inactivation^39^. Exploration of structural mechanisms between SLAC1 and two kinases, OST1 and CPKs, may reveal more evidence to narrow down the ambiguity between the two hypotheses. To date according to in planta studies, S59 and S120 phosphorylation sites in SLAC1 are required for intact ABA-induced stomatal closing but the molecular mechanism that translates the phosphorylation signal into the opening of the pore is still not understood. All these point to the possibility that SLAC1 may be regulated by at least two mechanisms in ABA signalling network, a ‘shortcut pathway’ activated by OST1 and Ca^2+^ induced second ‘loop pathway’^38^ activated by CPKs. Therefore, we assume Ca^2+^ elevation independent OST1 activation of SLAC1 in this study (This issue can be completely resolved only when definitive evidence is found in future). Background level Ca^2+^ was considered as the initial level of Ca^2+^ in the model because Ca^2+^ is one of the most ubiquitous molecules available at a low micro-molar level in plant cell cytoplasm^19^ regardless of active ABA signalling, hence requirement of background level Ca^2+^ for any communication was not considered in the model.

Since Ca^2+^ elevation did not influence the probability of closure (Fig. 5(A)) and downstream events, the role of Ca^2+^ elevation should be secondary in rapid stomatal closure consistent with the finding of the previous Boolean model^11^ and therefore, it does not fit into a functional or temporal hierarchy in the system. However, Ca^2+^ activity is seen even during the first phase of rapid stomatal closure, without any solid evidence for a key role for Ca^2+^ in it. The previous Boolean model^11^ was also not able to provide complete clarification about the role of Ca^2+^ in rapid stomatal closure. Therefore, the following questions were explored to identify the significance of Ca^2+^ elevation in the first phase of stomatal closure. Additionally, through the following investigations we attempted to further question both existing hypotheses about the role of Ca^2+^ in ABA signalling.

## Ca^2+^ elevation accelerates stomatal closure

It was suggested in the experimental literature that Ca^2+^ does accelerate stomatal closure^5^. The previous ABA signalling model^11^ also reports that the disruption of Ca^2+^ elevation leads to a slower ABA response than wild-type. However, there is still a need to explore this hyposensitivity further to quantify the level of acceleration and to properly reason how the presence of Ca^2+^ elevation accelerates stomatal closure. Besides, more molecular links between Ca^2+^ and the two major functional sets (osmoregulation and Actin rearrangement) have been revealed since the 2006 ABA model and our model has incorporated them. This advancement of network topology improved the understanding of network functioning while preserving the core behaviour observed in the 2006 ABA model. This was because with the new network topology we were able to properly define some of the crucial regulators of the system and better define fine dynamics of the system (e.g., OST1 is primarily responsible for activating osmotic regulation by directly controlling several ion channels but the main regulatory links of OST1 were missing in the 2006 ABA model). Therefore, the time taken by ABA signalling systems, one with Ca^2+^ signalling pathway intact and the other with no Ca^2+^ regulation, to achieve the final goal in response to a given set of initial conditions (1,000 here) was compared. Consistent with the previous model^11^, the results revealed that when the system has its Ca^2+^ regulation intact, the stomata close faster than in the Ca^2+^ knocked out system (Fig. 5A).

Figure 5(A) shows 80% stomata in the sample in both systems closed by 13 min. There is no much difference between the two systems in the cumulative probability of closure in the initial steps (5–7.5 min). The insignificance of Ca^2+^ in these initial time steps may be because the random starting conditions that produced the fastest closure, even without Ca^2+^, may have already satisfied all the conditions needed to facilitate stomatal closure. The difference in the closure probabilities between the two systems is clearer, however, from 8–13 min. As indicated in Fig. 5(B), the number of stomata closed by 8–11.5 min is higher in the Ca^2+^ dependent system (44% of the sample stomata), which is 11% higher than the number of stomata closed in the Ca^2+^ independent system. In contrast, the highest percentage of stomata in the Ca^2+^ independent system closed within 12–13 min of getting the ABA signal. We repeated the same simulation with another two samples of 1,000 stomata and noted that, on average, 10–20% of the sample stomata were accelerated by Ca^2+^ elevations and the acceleration was by 0.5–5 min. Further, we observed that the majority of Ca^2+^ accelerated stomata (>25%) closed just 0.5 min faster and very few (<1%) showed 5 min accelerations. This indicates that the gain in acceleration is mostly around the lower end of the time scale (≈0.5 min). The model’s ability to shed light on these patterns of acceleration is another advantage of using real timing. From the biological perspective, even the slightest acceleration in stomatal responsiveness may still provide significant benefits to guard cells under actual stress conditions^40,41^. This is because as ABA induced stomatal closure is fast acting, even a slight acceleration can evade the negative control of elements that are at play to inhibit closure. Comparison of the degree of acceleration of the response time with the Mann-Whitney U test indicated that the time differences between the two systems are statistically significant with 0.02 probability of significance (Table S4). With this, we confirmed that Ca^2+^ elevations in the system do accelerate stomatal closure.

Further, we identified that this acceleration in closure is primarily due to the time taken to stabilize the activity of SLAC1 and actin rearrangement. The activation time for both SLAC1 and actin rearrangement is significantly lower in the Ca^2+^ dependent system (with <0.001 probability of significance) (Table S5). This is another finding made possible by the real timing used in the model. This is supported by the biological fact that the ABA induced Ca^2+^ elevation enhances SLAC1^8,42^. Current scientific arguments^43^ about the biological importance of Ca^2+^ to guard cell signal transduction also highlight the importance of Ca^2+^ for the rapid regulation of SLAC1 by stimulating CPKs. However, ABA also triggers OST1 that we assumed can activate SLAC1 independently of Ca^2+^ as explained earlier. According to our model results, Ca^2+^ and Ca^2+^ dependant CPKs help the system accelerate SLAC1 but they alone cannot activate SLAC1 without having OST1 in the system consistent with previous experimental findings^33^. It was reported in the literature that CPKs and OST1 phosphorylate different amino acid residues of SLAC1^33^, hence working together on different sites can better activate the channel. Some literature also reports that elevation of Ca^2+^ triggers actin reorganization in guard cells^44,45^. Supporting this, our model showed that having Ca^2+^ in the system accelerates actin reorganization via strengthening ROS producing pathway (ROS regulates Actin depolymerizing protein ARP2/3) and reversing the action of some Actin binding proteins to support stomatal closure. Since SLAC1 regulation and actin rearrangement are essential to close stomata, these findings signify the role of Ca^2+^ during the first phase of stomatal closure. These results conclude that ABA signal transduction can achieve improved rapidity in stomatal responsiveness with the elevated Ca^2+^ levels in the system indicating that Ca^2+^ elevation has a role to play during stomatal closure in addition to its potential role in closure maintenance.

## Ca^2+^ elevation improves resilience of the system

As the role of Ca^2+^ elevation was shown to be potentially non-essential in the first phase of ABA signalling but shown to accelerate closure, we checked for any other secondary roles of Ca^2+^. The previous model^12^ reported that perturbations of Ca^2+^ (or Ca^2+^ influxes), when combined with disruptions in PLD (Phospholipase D), PA, GPA1 (G protein α subunit1), or pH, lead to stomatal insensitivity to ABA response. Their results signified that Ca^2+^ elevation is required for the system to close stomata when pH, K^+^ efflux or the S1P (Sphingosine-1-phosphate) –PA pathway are perturbed but how Ca^2+^ safeguards the signalling flow of these pathways still remains to be explored.

With the extended topology and real time updating method, we studied how Ca^2+^ elevation in ABA signalling network resists these reported perturbations and if there are any other such important perturbations. The importance of Ca^2+^ in resisting perturbation to important functional events of the system was evaluated by comparing the system performance in the wild type with the Ca^2+^ knocked out system when functional links or elements of the important functional sets (Fig. 2) are perturbed in the ABA signalling system.

**Figure 6:**
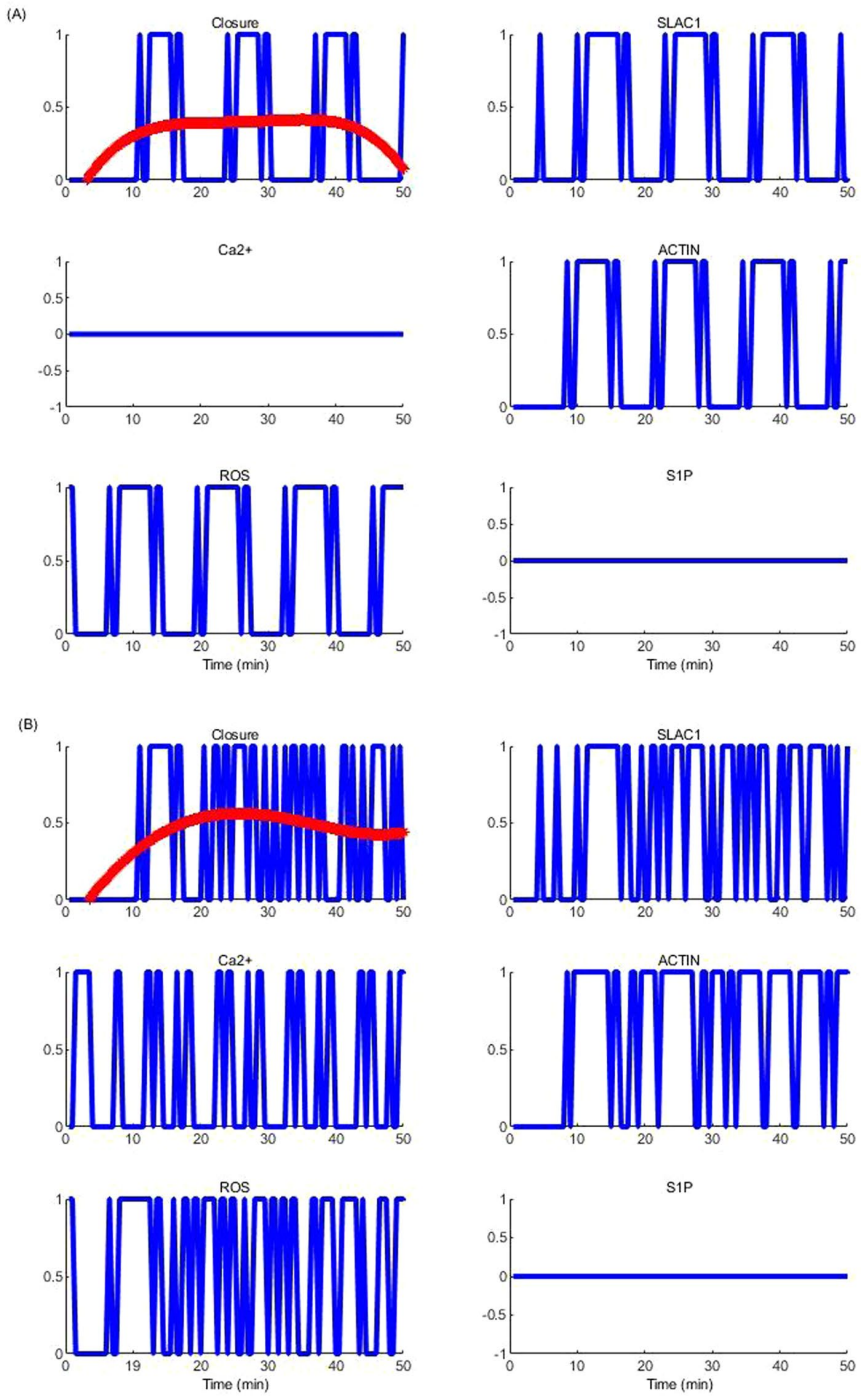
Importance of Ca^2+^ to the system when the lipid signalling pathway is altered. Evolution of temporal dynamics of Stomatal closure, SLAC1, Ca^2+^, Actin and ROS in ABA signalling in (**A**) Ca^2+^ and S1P knocked out system; and (**B**) S1P perturbed Ca^2+^ intact system, for the same initial conditions. Two smooth curves in red compares the level of stomatal closure in each case.

Our results revealed that Ca^2+^ cannot restore the system when the hub elements of each major functional set (osmoregulation (SLAC1 and Polarization), cytoskeleton rearrangement (Actin), ROS signalling (ROS) etc.,) are perturbed. However, results showed that Ca^2+^ plays an important supporting role in the absence of upstream regulatory elements or functional links of these hub elements by acting as a balancing loop to continue the downstream signalling flows. It was noted that some of the findings of the previous model^12^ are conserved in our model results; for example, perturbed lipid pathway (sphingolipids, PA) or plant pH together with Ca^2+^ produced drastically impaired stomatal closure (not stomatal insensitivity) but undisturbed Ca^2+^ elevation becomes beneficial to the system to withstand such perturbations (Fig. 6).

Our model further identified that this stomatal impairment is because lipid pathway and pH transmit the drought signal to keep the guard cell ROS production stable (see Fig. S1). ROS is another critical mediator in ABA signalling that communicates with all the major functional sets of the system. ROS controls guard cell osmoregulation via increasing the current intensity of GORK channel^46^ and regulating redox-sensitive proteins such as MAPK (mitogen activated protein kinase)^47^, which is an essential component for SLAC1 activation. Moreover, ROS regulates actin rearrangement via facilitating disassembly of actin filaments by weakening the inter-monomer bonds of the filaments^48^. Guard cell Ca^2+^ signalling is also supported by ROS via initiating Ca^2+^ influxes to the cytosol by adjusting the gating properties of the plasma membrane Ica channels^49^ and further facilitating NO production^50^ to pump Ca^2+^ from the internal organelles. Lipid signalling, especially Sphingolipid signalling, is very important to generate phosphatidic acid (PA), which mediates all the functional sets of the ABA signalling through the production of ROS^51^, inhibition of PP2C^52^ (Protein phosphatase type 2C) and inhibition of AHA1^53^. We observed that perturbed lipid pathway (by switching off S1P from Ca^2+^ intact and knocked out ABA signalling systems) altered the production of ROS and thereby altered osmoregulation and structural rearrangements resulting in defective stomatal behaviour in response to ABA. Of the two perturbed systems, the one with intact Ca^2+^ repeatedly attempts to reach stomatal closure by evoking Ca^2+^ dependent pathways (Fig. 6(B)) to stabilize the activity of SLAC1 and structural rearrangements compared to the Ca^2+^ knocked out system (compare Fig. 6(A) with Fig. 6(B)). As shown in Fig. 6(A), stomatal closure is inconsistent throughout the period of simulation when Ca^2+^ is not available in the system. This comparison is clearer with the smoothed dynamics (red lines is quadratic fit) on both graphs in Fig. 6(A,B), which clearly show that when Ca^2+^ is available guard cells achieve a better steady level closure. Near normal activity of SLAC1 was observed in ~50% of the sample stomata with undisturbed Ca^2+^ signalling pathway. The model showed that this is due to Ca^2+^ that attempts to restore the ROS production and thereby activate MAPK (mitogen activated protein kinase) which is an essential regulator of SLAC1 by inducing respiratory burst oxidase, RbOH, via CPKs. The model further reveals that restoration of ROS production by Ca^2+^ also stabilises structural rearrangements. This has support in the literature such that ROS supports structural rearrangement of guard cells via regulation of actin-related protein-2/3 (ARP2/3)^54^ and PI3P (Phosphatidylinositol 3-phosphate) mediated actin depolymerization by weakening the inter-monomer bonds of actin filaments^48^. Thus our model was able to elucidate how Ca^2+^ becomes required for engendering stomatal closure when upstream regulators of ROS production are perturbed by revealing the importance of ROS production through Ca^2+^ dependent pathway consistent with experimental literature^55^. In addition, literature supports that Ca^2+^ itself also acts as a mediator for guard cell cytoskeleton rearrangement on concentration basis, such that at low Ca^2+^, actin filaments are bundled by actin crosslinking proteins, whereas elevated Ca^2+^ levels modulate capping and depolymerization^56^, facilitating the disassembly of actin, and our model has captured these regulatory mechanisms.

Similar behaviour was observed in response to perturbations to plasma membrane QUAC (R (Rapid) type malate efflux channel, an essential element in osmoregulation) (see Supplementary Fig. S2) where Ca^2+^ promotes stomatal closure by facilitating osmoregulation through regulation of the vacuolar malate transport channels (*e*.*g*., ATALMT6). Moreover, the model output displayed a reduction in SLAC1 activity when both QUAC and Ca^2+^ were perturbed but restored SLAC1 channel when Ca^2+^ was re-established in the system indicating the interconnectedness of signalling events^57^ (compare SLAC1 panels in Fig. S2(A,B)). Interestingly, it was noted that GORK activity was untouched or poorly disturbed in all above perturbations but SLAC1 was the most sensitive to Ca^2+^ regulations (Fig. S3). Considering all these results, it can be concluded that Ca^2+^ plays an important facilitator/overseer role in emergency situations by strengthening the communication between important hub elements of the system to stabilize osmoregulation and structural rearrangements to achieve the end goal of stomatal closure.

**Figure 7:**
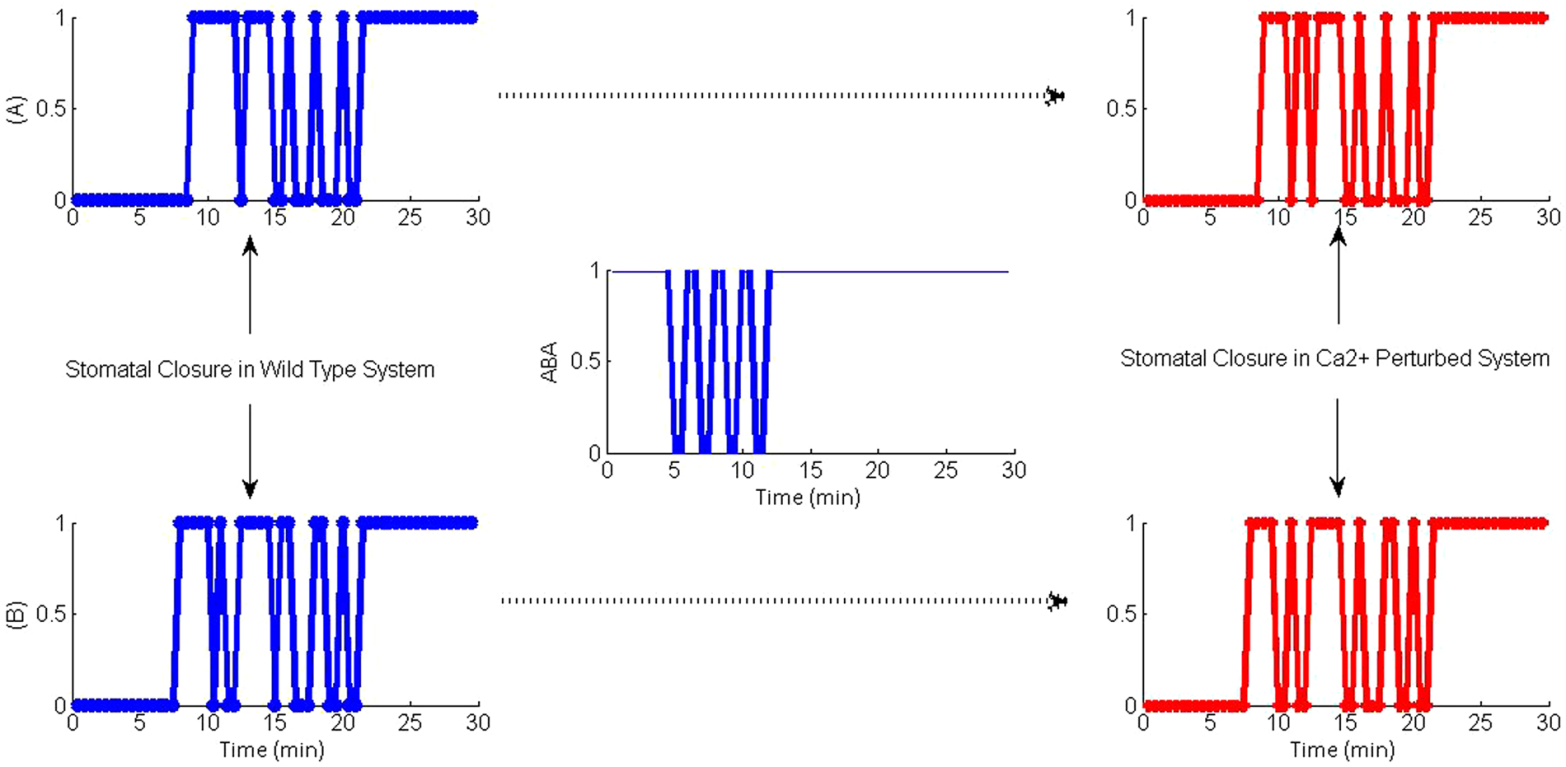
Stomatal closure in the WT system (blue) and the Ca^2+^ perturbed system (red) (**A**,**B** are two examples) when the ABA signal was initially provided as a pulse with 1 min delay between each pulse.

Finally, the importance of Ca^2+^ in situations where any delays or interruptions to the continuity of the ABA signal was evaluated by generating a perturbed system that experienced a delay in getting a continuous ABA signal. This was carried out by introducing a randomly selected pattern of ABA signal comprising a series of pulses, each accompanied by a fixed delay, which converged into a continuous signal 12 min later (middle graph of Fig. 7). It was noted that when the ABA signal was set as pulses and there was no active Ca^2+^ signalling in the system, guard cells could not achieve steady level closure (Stomatal closure tends to show oscillatory behaviour) as shown in red colour panels in Fig. 7(A,B) showing results for two experiments. Even with Ca^2+^, closure tends to show oscillatory behaviour but Ca^2+^ elevations tend to stabilize stomatal closure by acting as a balancing loop that minimizes the fluctuations and, thereby reduce the potential damage to guard cells (compare blue and red panels of Fig. 7). However, as the figure shows, in the absence of a continuing ABA signal, Ca^2+^ dependant system could not completely take over the role of Ca^2+^ independent pathways to achieve stomatal closure. Closer examination of model results revealed that even with no continuous ABA signal available in the system Ca^2+^ dependant pathways transiently activate SLAC1 to maintain osmoregulation. This is because the transient behaviour of Ca^2+^ regulated CPKs is evident in the model output, which attempts to maintain osmoregulation via ROS production and SLAC1 regulation with some success. However, the behaviour of Ca^2+^ governed CPKs should be biologically validated.

Similarly, model results revealed that Ca^2+^ dependant pathways attempt to sustain cytoskeleton rearrangement as observed in wild type plants but it does so with less success than that of osmoregulation. This may be because in comparison to osmoregulation, structural rearrangement is poorly defined in the model due to the lack of knowledge of the mode of action of actin regulatory proteins. This may explain the closure instability in the Ca^2+^ intact system as well.

According to the current knowledge captured by our model, actin disassembly is a cooperative effect of several actin regulatory proteins (SCAB1 (Stomatal Closure-Related Actin Binding Protein1), ARP2/3, ABPs (Actin binding proteins) and ATRAC1 (Rho-related GTPases), as in Fig. S1). Of these proteins, ARP2/3, ABPs and ATRAC1 can be regulated through Ca^2+^ dependent pathways as well. Conversely, Ca^2+^ could not activate SCAB1, a protein that has been found essential for structural rearrangements, due to the direct regulatory connection between SCAB1 and ABA, where the details of the regulatory mechanism between these two molecules are not yet known. Therefore, our model shows the necessity of ABA induced Ca^2+^-independent pathways to aid Ca^2+^-dependent signal transduction branch to achieve stable stomatal closure. However, if future experiments reveal a connection between SCAB1 and Ca^2+^ and/or CPKS, possibility exists for Ca^2+^ to take the role of ABA upon its discontinuation.

## Conclusions

In this study, we assembled an extended ABA network with the most current knowledge available at the time that incorporated a number of new important regulatory nodes and links. In particular, Ca^2+^ regulatory systems and their links to other important functional sets of ABA signalling were refined. We showed that the Asynchronous Boolean ABA model that was updated using real timing allowed us to produce system outputs close to biology (e.g., timing of ABA network events and behaviour of model mutants). Thus, the findings from our model are biologically validated and, therefore, insights from the model are useful. The model elucidated the role of Ca^2+^ and related mechanisms in greater clarity and detail than before. Specifically, it predicts that Ca^2+^ elevation is essentially not needed in rapid stomatal closure as stomata can close independently of elevating Ca^2+^. However, the system benefits in various ways from having Ca^2+^ in the system as a balancing loop to achieve faster and steady level closure. The previous model also predicted that Ca^2+^ can accelerate stomatal closure. Our model was able to provide the reason for it which is that Ca^2+^ accelerates stomatal closure through enhancing the activity of SLAC1 and actin rearrangement. Another new finding is that the model results identified SLAC1 as the most sensitive to Ca^2+^ regulations, much more so compared to GORK, and it was supported by experimental literature^33^.

Further, our model predicted the role of Ca^2+^ in enhancing closure resilience, similar to the previous model. With our model, we could go further to answer the question of how Ca^2+^ elevation enhances resilience (resistance to perturbation of important functions – e.g., S1P, QUAC, and disturbed ABA signal) of the system. According to the results, the cross-talk between Ca^2+^ and ROS, also supported by literature^58^, strengthens SLAC1 activity via direct regulation of CPKs by Ca^2+^ and indirectly via ROS regulation to induce MAPK, an essential regulator of SLAC1. In addition to these molecules, GHR1 (Guard Cell Hydrogen Peroxide-Resistant1) is also known as an essential regulator to activate SLAC1. The most recent literature observes that ROS and MAPK are involved in regulating SLAC1 through GHR1 in CO_2_ induced signalling^59^ but is not confirmed in ABA signalling. It was further observed in the oocytes expression system that GHR1 physically interacted with, phosphorylated, and activated SLAC1^60^. In the absence of confirmed mechanism of these molecules in guard cell ABA signalling, we assumed that ROS is an essential regulator for MAPK and MAPK is essentially required for activating SLAC1. As we have used timing from MAPK to SLAC1, ignoring putative nodes in between may not make a difference to the model output unless a novel regulatory mechanism for GHR1 is revealed. However, GHR1 also can be added to the model for obtaining more precise output once more information is available on this regulation. The cross-talk between Ca^2+^ and ROS regulating pathway further supports guard cell structural rearrangement by providing balancing loop regulations to Actin regulating proteins such as ARP23.

Updating the model using real timing provided useful insights into Ca^2+^ dynamics: It revealed biologically/experimentally consistent timing as reported in the literature for activation of Ca^2+^ during stomatal closure. It produced the parameters of Ca^2+^ oscillations (amplitude and duration) (i.e., attractor dynamics) closer to reality as well as the timing of occurrence of related events in the Ca^2+^ system. It helped quantify the level of acceleration of closure due to Ca^2+^and show that Ca^2+^ induced acceleration is statistically significant. It also allowed us to show that Ca^2+^ oscillations are sustained without decay in our model due to longer positive feedback loops. Previous model proposed this as a requirement for sustained oscillations and our model was able to confirm it with evidence. Further, timing helped explain the reason for a number of observations from the previous model and our model (e.g., how Ca^2+^ accelerates closure and how Ca^2+^ helps the system recover from the effects of some perturbations as stated above). Timing also helped us reveal the statistically significant reduction in activation time for both SLAC1 and actin rearrangement in the Ca^2+^ dependent system compared to Ca^2+^ independent system.

Our model supports the hypothesis of existence of a Ca^2+^ independent signalling pathway to regulate stomata if the OST1/SLAC1 pathway works independently of Ca^2+^ to regulate stomata. In the absence of evidence for OST1 and SLAC1 relationship directly from planta data, the Ca^2+^ independence found for plant OST1 and SLAC1 in oocytes was used in this study. Currently, the evidence for independence as well as somewhat inconsistent evidence against it exist. Our results in terms of timing of ABA events and Ca^2+^ dynamics were shown to correspond well with biology. However, future experiments may confirm the validity of this assumption. Further, the results indicate the dependence of Ca^2+^ dependent pathway on Ca^2+^ independent signalling pathway as the former could not achieve stable stomatal closure as evident in the latter case. This is because CPK, the main protein that decodes the Ca^2+^ signal, shows oscillatory behaviour that in turn leads to oscillations in stomatal closure. However, the behaviour of Ca^2+^ governed CPKs should be biologically validated. Additionally, our model used evidence in literature that SCAB1 is essential for actin rearrangement and it is regulated by ABA. Results indicate that if future experiments reveal a connection between SCAB1 and Ca^2+^ and/or CPKS, possibility exists for Ca^2+^ to take the role of ABA upon its discontinuation.

The model does not support or oppose the recently proposed Ca^2+^ sensitivity priming hypothesis at physiological resting Ca^2+^ levels as the mechanisms to support this hypothesis need further exploration. There are few evidences for existence of Ca^2+^ sensitivity priming in plants (ex: in plant pathogen signalling)^61–63^ but future research needs to show that Ca^2+^ independent signalling in guard cells is truly Ca^2+^ dependent to support this hypothesis. In our study, basal Ca^2+^ did not activate CPKs because Ca^2 +^ → CPK interaction was defined in the model such that above basal Ca^2+^ concentration is required to activate CPKs based on the structural properties of CPKs. We plan to explore Ca^2+^ sensitivity priming issue with our model at the next stage of research as considerable activities of some CPKs at basal Ca^2+^ level have been reported^64^. Whether CPKs are essential regulators in activation of SLAC1 in response to ABA in guard cells requires further clarification. However, we can hypothesise that if OST1 is a critically limiting component in ABA signalling^65^ as it has multiple essential phosphorylating targets, SLAC1, QUAC and RbOH, then some mutual equilibrium between OST1 and CPKs may reinforce regulation of SLAC1 to its full potential.

Considering the emerging understanding that the timing is more crucial than the exact level of proteins, we believe that the system dynamics revealed by our model is a closer qualitative approximation of the real system based on our current understanding of ABA signalling. Further, having greater details of the continuously changing levels of proteins (as in continuous dynamics analysis) may not provide much additional insight into this *fast-acting* protein signalling system that does not involve gene expression. Our model outcomes provide avenues for future experimental investigations to further consolidate the understanding gained here. In this paper, our focus was on elucidating the role of Ca^2+^ in stomatal closure. Soon, we also intend to report on model outcomes for the whole ABA system response from the perspective of, and highlighting, all its functional sub-systems, including Ca^2+^ sub-system, to elucidate how the system regulates them concurrently in a well-coordinated and timely manner to achieve stomatal closure. In particular, in future, the new knowledge of Ca^2+^ and our model could potentially help develop drought resistant plants that survive the extremes of climate change.

## Materials and Methods

The extended network shown in Supplementary Fig. S1 was used to examine the dynamics of ABA signalling with asynchronous Boolean approach as explained below.

### Conversion of network into boolean logic functions

We defined a Boolean network (Supplementary text 1) in such a way that the logic functions define the state of system variables (proteins, etc.) in qualitative form denoted by 0 (below a threshold) or 1 (above it). We used logical operators “AND”, “OR” and “NOT” in Boolean functions to represent the influence of a node (variable) on another considering biological interactions and their mode of actions (Table S1). Operator “AND” represents the association of two or more regulators in a dependent manner where all the regulators are needed for the activation of a node. If the activation of a node can be induced by any of its regulators, a Boolean function combines these independent regulators with an “OR” operator. The operator “NOT” represents a negative regulation of a node by another.

### Updating scheme of the model

We incorporated network node edge delays (time delays) into an asynchronous updating scheme in order to provide the network nodes with realistic biological asynchrony. Here we assumed that proteins get activated according to the time required for the activation of their regulators. Therefore, in this analysis, we updated a network node at time ‘t’ based on the status and the processing time of each of its immediate regulators (Eq. 1). The nodes that do not have regulators are left in the initial condition throughout the simulation (assuming they are positively self-regulated).

1 $${x}_{i}(t)={B}_{i}({x}_{1}(t-{\tau }_{1i}),\ldots \ldots \ldots \ldots \ldots .{x}_{n}(t-{\tau }_{ni}))$$

where *B*_*i*_ is the Boolean function associated with node *i* and ***τ***_ji_ are the corresponding edge delays for a regulator node *j* of node *i*.

### Edge based delays

Through an extensive literature survey, we collected research findings on time delays of ABA signalling network nodes defining edge delay as the biological half activation time (**τ**) of a reaction. For clarity, for a simple example of a three node network where A → B ← C, edge delays are defined in such a way that if A induces half activation of B within *τ*_A_ and C half activates B within ***τ***_C_, ***τ***_A_ and ***τ***_C_ are considered edge delays associated with node B.

In situations where no direct information is available (roughly 30% of interactions), we collected data from similar interactions occurring in other signalling systems in plants, animals or humans. When edge to edge timing is not available for an intermediate step, we calculated time scales by decomposing a (larger) available time scale into a set of smaller time scales using a simple algebraic formula such that, for instance, if node A activates node C via node B (A → B → C), where the time scales for A → C (***τ***1) and B → C (***τ***3) are known, then the time scale for A → B (***τ***2) is calculated as the difference ***τ***1-***τ***3.

As a consequence of the unavailability of a single data set for the timescales of the ABA system, relative timings of the network edges were extracted from various sources; hence, the heterogeneity in information pool may introduce some noise to the model results. Therefore, the data set was carefully scrutinised to minimize the noise as this was considered the best option for the model. We observed a number of favourable justifications for pooling information from different sources in that the time delays in plant, human and animal systems were comparable in many reactions and time delays were consistent between ‘*in vitro*’ and ‘*in vivo*’ experiments^66,67^. Further, we observed that the half time does not differ significantly with dose changes of reactants because differences in substrate levels cause differences in peak level of the product formation but not the time taken to reach that level^68^. Importantly, experimental conditions (T^0^, pH etc.) were in physiologically acceptable levels for plants, despite system, varietal, and kingdom level differences^44,69^.

We collected experimental results from literature for all network reactions and calculated the mean activity time for each, which correspond to the biological timeframe of ABA induced stomatal closure. According to the data collected, time delays in the current system ranges from milliseconds to minutes (Supplementary Table S1). Use of an extensive data set on a millisecond scale in asynchronous modelling update would be computationally exhaustive. Therefore, timing data was condensed into a smaller number of time units coding time delays into a finite series of time intervals whereby all actual time delays falling into an interval was given the same fixed delay corresponding to that interval. Specifically, coding for the model update is defined in such a way that each 0.5 min equals 1 time unit (e.g., 0–0.5 = 1, 0.5–1 = 2, ……… 14.5–15 = 30). Variability within 30 sec was ignored in the model (Supplementary Table S1). It is expected that this will make the model easy to handle. The coding will facilitate the organization and interpretation of the model by avoiding too lengthy simulation cycles further reducing random noise.

Interactions for which there was no information available in the literature (about 10%), we made realistic heuristic assumptions considering the most relevant factors/knowledge. In some situations, we generalized a reaction such that if a particular protein is phosphorylated independently by a number of kinases, we assumed that all such reactions happen on a similar timescale (e.g., OST1, MAPK, CDPK → SLAC1). Similarly, if one protein dephosphorylates several kinases, we assumed that all such reactions happen on a similar timescale (e.g., PP2C OST1, MAPK, CDPK, CIPK (CBL interacting protein kinase)). In some other cases such as the catalytic effect of an enzyme changing the concentration of an end product through an intermediary (OST1 → RbOH → ROS) where the time for each step is not known but the total time is known, the second step was assumed rapid. Finally, for non-biological nodes like ‘CLOSURE’, the smallest timescale was assumed for all its regulators.

### Updating operators for the model

We defined operators for the updating scheme considering the logic gates of corresponding nodes (Supplementary text 1 and Table S1). As an example, let B and C be two regulators of node A in a three-node network. If either B or C can independently regulate A, which combines the regulators B and C with a simple “OR” gate – $({B}_{(t-{\tau }_{B})}|{C}_{(t-{\tau }_{C})})$, updating operator for A at a given time (t) is defined as in Eq. 2.

2 $${A}_{t}=\{\begin{array}{ll}{B}_{(t-{\tau }_{B})}, & t\le {\tau }_{C}\,and\,t > {\tau }_{B}\\ {C}_{(t-{\tau }_{C})}, & t\le {\tau }_{B}\,and\,t > {\tau }_{C}\\ {B}_{(t-{\tau }_{B})}|{C}_{(t-{\tau }_{C})} & t > {\tau }_{C}\,and\,t > {\tau }_{B}\\ {A}_{t-1} & otherwise\end{array}$$

where *τ*_B_ and *τ*_C_ are the edge delays from B to A and C to A, respectively, ${B}_{(t-{\tau }_{B})}$ is the status of B at $t-{\tau }_{B}$ time point, ${C}_{(t-{\tau }_{C})}$ is the status of C at $t-{\tau }_{C}$ time point, and $A(t-1)$ is the status of A at previous time point.

If both regulators are needed for the activation of node A, which combines the regulators B and C with an “AND” gate (A = B and C), the updating operators are defined as in Eq. 3.

3 $${A}_{t}=\{\begin{array}{ll}{B}_{(t-{\tau }_{B})}\,and\,{C}_{(t-{\tau }_{C})} & if\,t > {\tau }_{B}\,and\,t > {\tau }_{C}\\ {A}_{t-1} & otherwise\end{array}$$

### Simulation of network dynamics

With Boolean functions and associated time delays, the network is simulated for 100 discrete time points with a large number of random initial states (10,000) to study the most frequently visited states/attractors of the network. The state of input node ABA was always kept at on (1) state and the target node (CLOSURE) set at the off (0) state for all initial conditions. As the stomata is considered in the open stage, the initial conditions of nodes, which satisfy the stomatal opening were considered active (Malate = 1, KAT1 (Plasma membrane K+ influx channel) = 1, AHA1 = 1, DEPOLAR (Plasma membrane depolarisation) = 0, PYR = 0 and PP2C = 1) when the simulation begins. Random initial states were assumed for all other nodes (>80%) where initial states were not known *a priori*. The probability of success (stomatal closure) was obtained from Eq. 4 as specified by Li *et al*.^11^.

4 $$P{(closure)}^{t}={\sum }_{j=l}^{N}{S}_{closure(j)}^{t}/N$$

where ${S}_{closure(j)}^{t}$ is the state of the node “CLOSURE” at time t in the *j*^th^ simulation and N is the total number of simulations.

The significance of a specific node on the global behaviour of the network was evaluated by keeping its state below the threshold level (0) over the simulation to reproduce biological knockouts and/or pharmacological inactivation. Further, overexpression of a particular node was mimicked by constantly expressing its state above the threshold level (1) throughout the simulation period.

### Evaluation of dynamics of WT and Ca^2+^ perturbed systems

The significance of Ca^2+^ to the system behaviour was studied by setting the state of Ca^2+^ node at inactive state (0) over the simulation. In order to see if having Ca^2+^ in the system accelerates closure, we compared the timing of stomatal closure for WT and Ca^2+^ knocked out systems. Comparison was done between time taken to close stomata by the two systems in response to a given set of initial conditions. Initial conditions used in the simulation were identical for both systems. We used 10,000 random initial conditions to simulate the dynamics of both systems. Time differences between the two systems for similar initial conditions were compared using nonparametric version of paired t-test, Mann Whitney U test, as the data set did not follow a normal distribution. Whether Ca^2+^ plays a role in improving system robustness was tested by perturbing crucial elements in the functional sets in the WT and Ca^2+^ knocked out systems and comparing the difference in the stability of stomatal closure.

## Electronic supplementary material

Supplementary Information
