# Supplementary material for: Boolean Calcium Signalling Model Predicts Calcium Role in Acceleration and Stability of Abscisic Acid-Mediated Stomatal Closure
Source: Sci Rep. 2018 Dec 5;8:17635. doi: 10.1038/s41598-018-35872-9 (PMC6281740; doi:10.1038/s41598-018-35872-9)
Supplement: Supplementary file 1 — Supplementary Information [file 41598_2018_35872_MOESM1_ESM.doc]

**Supplementary Materials**

**Boolean Calcium Signalling Model Predicts Calcium Role in Acceleration and Stability of Abscisic Acid-Mediated Stomatal Closure**

Pramuditha Waidyarathne and Sandhya Samarasinghe


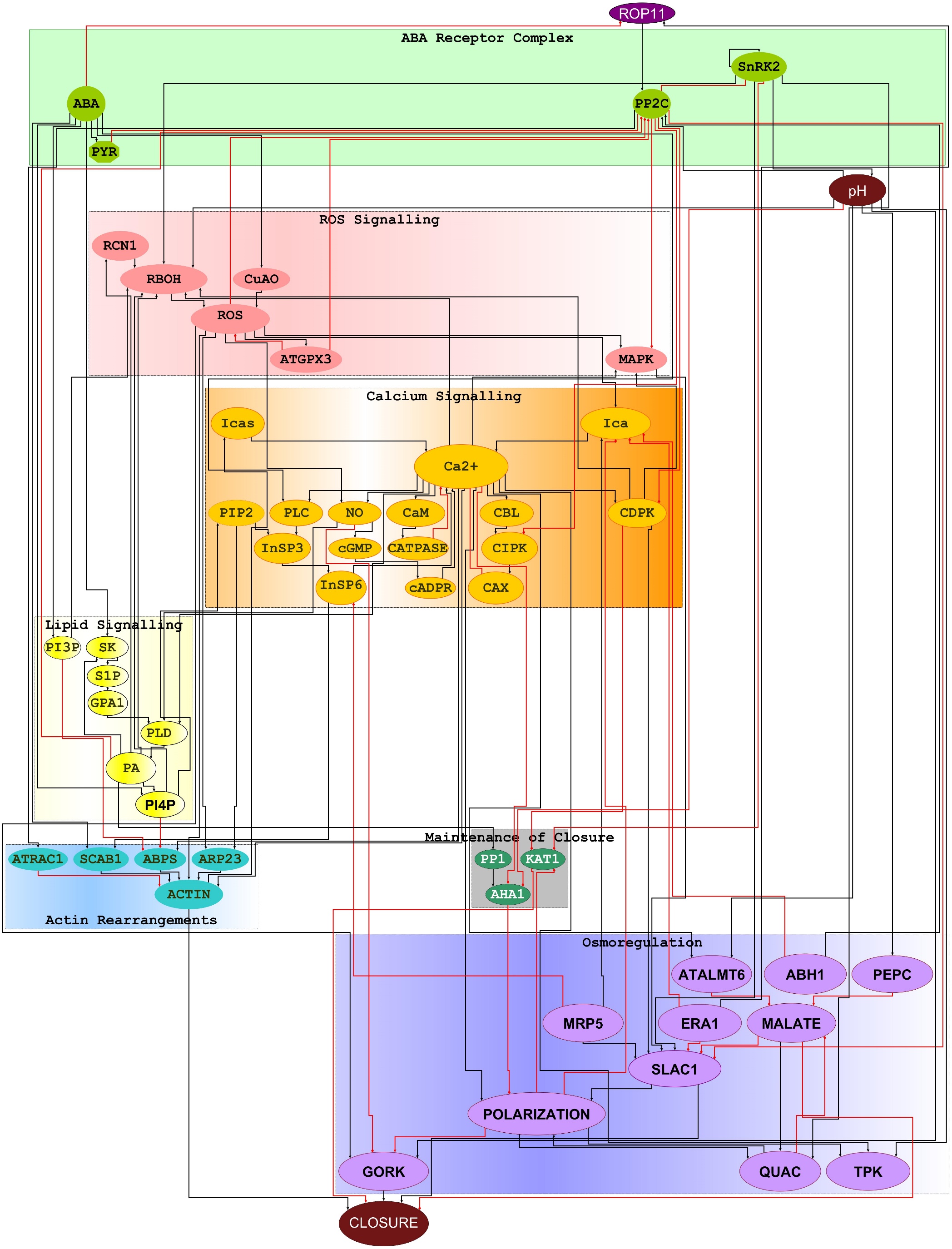


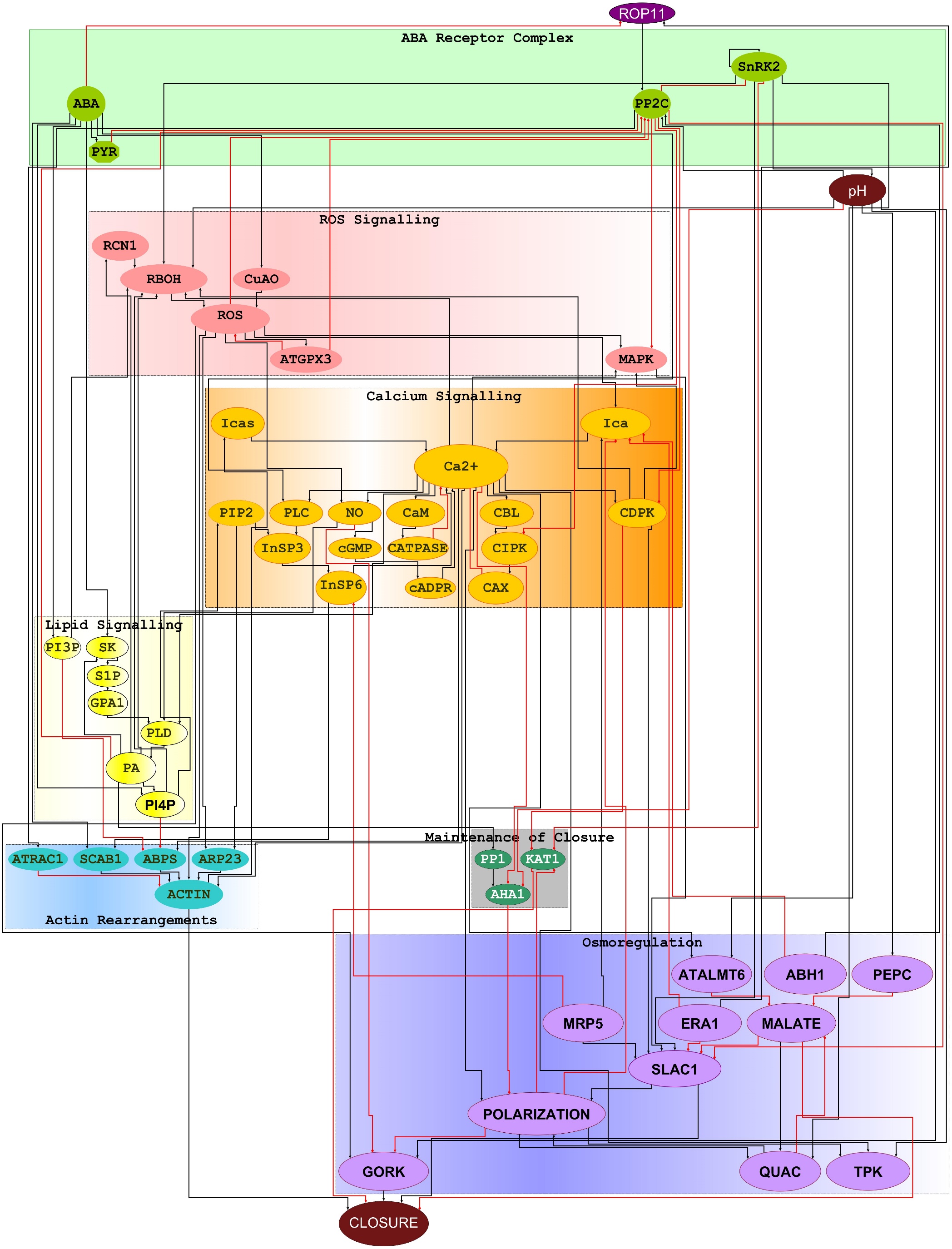


Figure S1: Detailed view of the extended ABA signalling network. Description of all the elements and interactions and their sources are given in the description below.

Following is a description of all the elements and interactions in the network shown in Fig. S1 and their role in ABA induced stomatal closure as obtained from literature. This network in Fig. S1 was used to develop the Boolean model described in the paper to investigate the role of Ca2+ in stomatal closure.

Signal transduction and perception occurs when the signalling molecule (ABA) activates its receptor, PYR/PYL/RCAR (pyrabactin resistance/PYR like/regulatory component of ABA receptor), allowing it to trigger or unleash a signalling molecule SNF-related kinase 2 protein (SnRK2) that is kept inhibited by protein phosphatase 2Cs (PP2C) in unstressed cells. This is because PP2C lacking PYR/PYL/RCAR binding strongly inactivates SnRK2 by dephosphorylating it ^1,2^. The light green box in Fig.S1 depicts how the ABA receptor complex works on releasing SnRK2 to proceed with signal transduction. Release of SnRK2 from PP2C facilitates guard cell osmosis through activation of various types of ion channels, which is the first task of the ABA signalling cascade.

Shrinkage of guard cells is a result of water efflux from these cells. Guard cell signalling regulates water efflux by the reduction of osmotic pressure inside the guard cells by releasing osmolytes from the vacuole to the cytosol and then to the cell exterior by regulating ion channels on plasma membrane and vacuolar membrane. As shown in the purple box in Fig. S1, various proteins and other molecules are involved in regulating these channels. The guard cell plasma membrane slowly activating anion channel (SLAC1) mediates the reduction of anions, mainly Cl^-^ ions, to promote turgor reduction. The required level of K^+^ concentration is achieved through the positive regulation of the guard cell outward rectifier K^+^ out channel (GORK) and the outward rectifying vacuolar ion channel (TPK1).

Phosphorylation is a pre-requisite for complete activation of SLAC1 but the mechanism that facilitates SLAC1 opening by phosphorylation is still unknown^3^. As shown in the purple box in Fig. S1, SLAC1 activation depends on number of regulators (SnRK2, Ca^2+^, CDPK, malate, MAPK, ERA1, ABH1, MRP5 and PP2C) in ABA signalling. Protein kinase SnRK2 phosphorylates the channel^4^. Ca^2+^-dependent activation of SLAC1 is either stimulating the peak activity or inhibiting slow inactivation^5^ by phosphorylating the channel through activation of calcium dependent protein kinases (CDPK). Both CDPKs and SnRK2 can independently regulate SLAC1 as the activation sites are different for SnRK2 and CDPK^4^. SnRK2 can activate the channel at the basal cytosolic Ca^2+^ level but CDPKs need Ca^2+^ levels to rise to activate the channel. In contrast, PP2C can directly bind to the activating site of CDPK on SLAC1 and down-regulates SLAC1 by dephosphorylation^4^. Both ABA and Ca^2+^ fail to activate SLAC1 in plants lacking mitogen-activated protein kinases (MAPK). There is evidence to indicate that MAPKs (AtMPK9/AtMPK12) regulate SLAC1, acting downstream of ROS^6^ (pink colour box), but it is not yet known whether the activation of SLAC1 is direct or indirect^7^. To date, the literature supports the hypothesis of indirect regulation of SLAC1 as it is unlikely to be directly regulated by MAPKs^8^. The literature suggests the availability of at least two MAPK pathways that convey diverse signals in ABA signalling to regulate stomatal aperture (e.g., SLAC1 regulation), but details of them have not yet been unravelled^7^. In addition, an ATP binding cassette transporter, MRP5, also enhances SLAC1 activity. There is evidence to consider the b-subunit of Arabidopsis farnesyltransferase (ERA1) and an mRNA cap binding protein (ABH1) as negative regulators of the channel as both ERA1 and ABH1 mutants show enhanced S-type currents.

The loss of anions through SLAC1 changes the polarity of the guard cell plasma membrane making it more positive (depolarized), which provides the main pathway for the efflux of K^+^ from guard cells through K^+^ efflux channel GORK, where the activation of the channel depends on voltage-dependent gating ^9^. Cytoplasmic pH acts on GORK by shifting the half maximal activation voltage to a more positive voltage^10^. Additionally, increased cytosolic pH further promotes the efflux of K^+^ by increasing the number of available GORK channels on the plasma membrane (a membrane delimited pathway)^11^. Reactive oxygen species (ROS) can also regulate the activities of the guard cell GORK channels through post-translational modification^12^ promoting a positive regulatory mechanism on the active channel; however, ROS activation of GORK strongly depends on membrane depolarization^13^. In contrast, higher concentrations of nitric oxide (NO) (≥20 nM) (orange colour box) can reverse the activity of GORK through nitrosylation ^14^.

TPK1 is an outward rectifying vacuolar ion channel that is responsible for K^+^ efflux from the vacuole to the cytosol. This channel shows very strong selectivity for K^+^ and is activated by (Ca^2+^)_cyt_  ^15^ but not by tonoplast membrane voltage changes^16^. The open probability of the channel is at maximum around cytoplasmic pH 6.7 but under more alkaline pH, the current drops steeply and it decreases moderately under acidic pH ^16^. Due to the reduction of vacuolar K^+^ release, mutants of TPK1 plants slow down the stomatal closure ^16^. However, vacuolar K^+^ channels should be crucial to stomatal closure because more than 90% of the total K^+^ during the closure mechanism is from the vacuole. Therefore, the low functionality of TPK1 at the physiological pH range is evolutionarily more favourable for the regulation of stomatal opening when considering the importance of vacuolar K^+^ release for stomatal closure. There are reports about three types of K^+^ fluxes across the tonoplast: fast vacuolar (FV), slow vacuolar (SV) and K^+^ selective vacuolar channels. TPK1 belongs to the group of K^+^ selective vacuolar channels. The recently identified two-pore channel (TPC1) is considered as an SV channel; it can be regulated for the vacuolar efflux of K^+^ but according to literature it does not have any significance in ABA signalling. Therefore, we did not consider TPC1 as a player in ABA signalling. Theoretically, FV channels should display an instantaneous K^+^ efflux from the vacuole but molecular studies are necessary to better understand how FV channels are involved in guard cell osmoregulation (Pareek and Bohnert, 2010). As the detailed signalling chain is not complete for the tonoplast transport system and no vacuolar anion channels have been found yet, some studies suggest that slow vacuolar (SV) and K^+^ selective vacuolar channels are responsible for effluxes of both anions and cations (MacRobbie, 1997). Further, it is a well-accepted fact that K^+^ channels on the plasma membrane provide the dominant pathway for K^+^ efflux required for stomatal closure^17^.

Malate, as a divalent anion, often accompanies K^+^ cations in the guard cell cytosol to maintain electrical balance in the cell during stomatal opening and, therefore, it is important as an osmoticum to regulate guard cell turgor. [Carboxylation](http://en.wikipedia.org/wiki/Carboxylation) of [phosphoenolpyruvate](http://en.wikipedia.org/wiki/Phosphoenolpyruvate) (PEP) by PEP carboxylase (PEPC) in guard cells mediates the synthesis of malate as an intermediate product of photosynthesis. Therefore, stomatal closure needs to limit the production of malate by either modulating PEPC degradation, facilitating efflux of malate or converting it into osmotically inactive starch. When stomata are closing ABA acts negatively on PEPC and make it unavailable in the system (Schnabl et al., 1982) and this is possibly through inhibition of the pathway responsible for the degradation of the PEPC phosphorylating kinase (Monreal et al., 2007). It was further reported that malate itself downregulates PEPC activity when stomata are closing but insensitive when stomata open (Outlaw and Zhang, 2001). According to literature, both metabolism and transport of malate are important for regulating stomatal responses to diurnal changes, but malate transport seems more important in ABA signalling (Penfield et al., 2012). Transportation of malate is mainly through the regulation of the plasma membrane rapidly activating anion channel (QUAC) and vacuolar malate transport system (ATALMT6 in Fig. S1). The QUAC on the plasma membrane is normally activated by plasma membrane depolarization and phosphorylation (Hedrich et al., 1990). When malate is present as a substrate in the medium, the active potential becomes more negative (hyperpolarized) (Raschke, 2003). The activation mechanism of QUAC is not yet fully understood but recent literature highlights the importance of SnRK2 protein kinase for the activation of QUAC, possibly through phosphorylation (Imes et al., 2013). AtALMT6, a member of the aluminium-activated malate transporter family located on the vacuolar membrane, mediates inward rectifying currents of malate under the regulation of micro-molar concentrations of [Ca^2+^]_cyt_ (Meyer et al., 2011). The functional behaviour of accumulating or releasing malate through this channel is determined by the interplay between cytosolic malate and vacuolar pH to regulate the activation threshold depending on the tonoplast potential.

The blue colour box in Fig. S1 depicts the ABA induced actin regulatory network demonstrating how actin dynamics are regulated by [Ca^2+^]_cyt_, other proteins (PP2C, ARP2/3, SCAB1), phospholipids (PI3P and PI4P), ROS and Rho-related GTPases (AtRAC1) through their interactions with actin regulatory processes. Depolymerization of F-actin polymers in the cytoskeleton controls the disassembly of Actin filaments in guard cells. Rho-related GTPases (AtRAC1) inhibit the disassembly of actin filaments (Lemichez et al., 2001) by inhibiting the Actin binding and depolymerizing activities of the actin depolymerizing proteins. AtRAC1 is positively regulated by PP2C, where inactivation of PP2Cs can deactivate AtRAC1 (Lemichez et al., 2001). The actin-related protein-2/3 (ARP2/3) complex modulates actin remodelling in stomatal closure (Wang et al., 2011), possibly through enhancing the dispersal of actin bundles to finer filaments by exerting mechanical force on actin filaments and microtubules (Jiang et al., 2012). Regulation of ARP2/3 depends on the ABA induced ROS production, but the underlying mechanism remains elusive (LI et al., 2014a). PIP2 is also a possible target because it has been identified as the general regulator of ARP2/3 in other systems (Rozelle et al., 2000).The recently-identified plant specific stomatal closure-related actin binding protein1 (SCAB1) also mediates structural rearrangements by stabilizing the depolymerized actin filaments (Zhao et al., 2011). Details of regulators or mode of activation of SACB1 is not clear, but there is evidence suggesting it may be regulated by inositol phosphates (InSP3/InSP6) (Zhang et al., 2012). Mutation of SCAB1 exhibits stomatal insensitivity to ABA, delaying the switch of actin filament movement from a radial to a longitudinal configuration during stomatal closure (Zhao et al., 2011). In addition to the above proteins, there is emerging evidence supporting the involvement of phosphoinositides (PI3P and PI4P) in the modulation of actin dynamics by regulating actin depolymerization (Choi et al., 2008). Generally, it appears that PI3P and/or PI4P binding to actin bundling and crosslinking proteins, such as fimbrin, villin and profilin (ABPS in Fig. S1), releases these proteins from actin filaments to facilitate the access of actin depolymerizing proteins to filaments stimulating depolymerization (Huang et al., 2005). Another possible mechanism for the PI3P effect is through the regulation of ROS, which induces PI3P mediated actin depolymerization by weakening the inter-monomer bonds of actin filaments (Choi et al., 2008). A second messenger, [Ca^2+^]_cyt_, also acts as a mediator for guard cell cytoskeleton rearrangement on concentration basis, such that at low [Ca^2+^], actin filaments are bundled by actin crosslinking proteins, whereas elevated [Ca^2+^] levels modulate capping and depolymerization (Yokota et al., 2005), facilitating the disassembly of actin.

Orange colour box in Fig. S1 shows the ABA induced Ca^2+^ regulatory network, which comprises four Ca^2+^ influx systems and two Ca^2+^ efflux systems. Of the four different Ca^2+^ influxes, the initial contribution is from the voltage-dependent plasma membrane calcium channel (Ica), which transiently provides Ca^2+^ currents to the cytoplasm (Trouverie et al., 2008). In addition, there are two positive feedback loops to enhance the cytosolic Ca^2+^ concentration by pumping out Ca^2+^ from the internal organelles. The first feedback loop is between nitric oxide (NO) ↔ Ca^2+^) and the other is through phospholipase C (PLC) ↔Ca^2+^). Further, there are reports to indicate the contribution of mechanosensitive Ca^2+^ channels in the plasma membrane to the influx system at a later stage, due to actin filament rearrangements. Removal of Ca^2+^ from the cytoplasm is either through Ca^2+^-ATPases or H^+^/Ca^2+^ antiporter activity.

The voltage-dependent Ca^2+^ channel (Ica) plays a prominent role in ABA induced Ca^2+^ signalling by initiating a Ca^2+^ influx across the plasma membrane. Two distinct regulators, ROS and the plasma membrane with voltages above -100 mV (hyperpolarization), regulate Ica channel activity (Wang et al., 2013). There are three other proteins (b-subunit of Arabidopsis farnesyltransferase (ERA1), ABH1 protein and MRP5 transporter protein) in the ABA signalling system that also show regulatory effect on Ica, but the accurate mode of control of these proteins remains elusive.

Ca^2+^ elevation in the cytoplasm through endomembranes occurs via the feedback loop between Ca^2+^ and NO, which triggers the activity of a Ca^2+^ releasing second messenger, cyclic ADP Ribose (cADPR). Ca^2+^ releasing cADPR-gated channels, located in endomembranes of plant guard cells, release Ca^2+^ from the vacuolar and the endoplasmic reticulum to cytosol. Activation of this feedback loop (Ca^2+^ ↔ NO) depends on the generation of cytosolic ROS and Ca^2+^ induced CaM binding. According to literature, Ca^2+^ induced CaM directly binds to plant nitric oxide synthase-like enzyme (NOS) in an NADPH-dependent pathway to generate NO (Guo et al., 2003, Vidhyasekaran, 2014). NO is further synthesized by nitrate reductase (NIA1) in a ROS-dependent mechanism (Wang et al., 2010), which is possibly with a mediatory effect of Ca^2+^ (Neill et al., 2008). NO then induces the production of cyclic guanosine monophosphate (cGMP), which then stimulates the activity of cADPR to generate the Ca^2+^ influx. Inhibition of guanylyl cyclase (GC) completely suppresses ROS and NO induced [Ca^2+^]_cyt_ (Dubovskaya et al., 2011, Leckie et al., 1998).

Plant phospholipase C (PLC) is a plasma membrane bound enzyme which acts as the sole mediator for Ca^2+^ mobilization through the ABA induced phospholipid pathway and this is the second feedback loop, which releases Ca^2+^ from the internal organelles. Ca^2+^ binds to PLC and activates it by stimulating the hydrophobic surface, targeting membrane binding for enhanced catalytic activity of the enzyme (Rupwate and Rajasekharan, 2012). The literature provides further evidence that ABA induces the activity of PLC but the mode of action is not clear (Webb and Robertson, 2011); it may be through the G-protein-coupled receptor as in the mammalian pathway. In addition, there is evidence for NO-dependent activation of PLC but we suspect that NO regulation may be mediated through Ca^2+^ release. PLC stimulates the hydrolytic cleavage of phosphatidylinositol-4,5-bisphosphate (PIP2) to produce inositol-1,4,5-trisphosphate (InSP3). Phosphatidylinositol 4-phosphate (PI4P) acts as a precursor of PIP2 synthesis via phosphorylation by the relevant inositol kinases. Similarly, PIP2 can be hydrolyzed into PI4P by a variety of enzymes available.

In Arabidopsis plants, two peaks of InsP3 are observable (at 30 s and 30 min) in response to ABA (Perera et al., 2008). This is similar to another report which claims that ABA can enhance InsP3 level by 90% of the maximum within 10s (Lee et al., 1996). InSP3 is then rapidly converted into InSP6. This conversion may happen because plant cells lack InSP3 receptors and InSP6 can release Ca^2+^ faster than InSP3 with a 10-fold lower concentration level. Lemtiri *et al*., reported that InSP6 enhances [Ca^2+^]_cyt_ levels to a maximum within 1 s by activating vacuolar ion channels (Lemtiri-Chlieh et al., 2003). However, it was further reported in the literature that the InSP6 induced Ca^2+^ increase only lasts for few min and then decays to the resting level. This may be in relation to MRP5, an ATP binding cassette transporter with high affinity for InSP6, which transports InSP6 to the vacuole making InSP6 not available for channel regulation.

In addition to the three Ca^2+^ influxes discussed above, structural rearrangements may mediate a Ca^2+^ influx to the cytosol via stretched activated Ca^2+^ channels (Icas) (Zhang et al., 2007), where stabilized actin filaments block the channel activation.

There are two major types of Ca^2+^ efflux systems available in guard cells, Ca^2+^-ATPase and Ca^2+^/H^+^ exchangers (Bose et al., 2011). The major Ca^2+^-ATPase, which is responsible for hormonal signalling and [Ca^2+^]_cyt_ homeostasis, is auto-inhibited Ca^2+^-ATPases (ACA). Calmodulin (CaM) suppresses the auto-inhibitory action of ACA by physically binding to the auto inhibitory N-terminus of ACA. This interaction activates Ca^2+^ pumping by increasing the affinity for free Ca^2+^. Ca^2+^/H^+^ exchangers (CAX) are low affinity (K_m_ =10 – 15 µM), but high capacity Ca^2+^ pumps powered by proton-motive forces. In Arabidopsis, CAX1 and CAX3 are involved in Ca^2+^ homeostasis. Of them, CAX1 is strongly expressed in leaves where it is located on the tonoplast membrane and transports Ca^2+^ into the vacuole (Robertson, 2013) and undergo post-translational regulations through an auto-inhibitory N-terminus similar to ACA. Further, protein–protein interactions are also possible for the activation of CAX.

There are calcium sensor proteins; some are freely available in the guard cell cytosol, and some are attached to membranes, which capture the Ca^2+^ signature and process the information to decode them for transmission towards the final target, stomatal closure. Calcium dependent protein kinases (CDPKs) are high Ca^2+^ affinity sensor proteins harbouring regulatory domains at C-terminal EF hand for calcium-binding and an auto-inhibitory junction region. Upon binding to Ca^2+^, CDPK undergoes conformational changes facilitating kinase activity by covering the auto inhibitory domain, which allows the active site of the kinase domain to be available for its substrates. The full activation of the kinase is achieved through the parallel process of auto-phosphorylation. Of 34 CDPKs available in Arabidopsis, few are identified as regulators/targets of ABA signalling. Regulatory activities of CDPKs in ABA mediated stomatal closure are: CDPK1(CPK10) inhibits the activity of inward K^+^ channels (Zou et al., 2010), CDPK3(CPK6) and CDPK6 (CPK3) activate SLAC1 and regulate Ca^2+^ channels (Mori et al., 2006), and CPK5/CPK6 and CPK4/CPK11(CDPK2) regulate ROS production (Boudsocq et al., 2010). CDPKs that function in ABA induced stomatal responses are localized in the plasma membrane and the nucleus. Activation of CDPK depends on the availability of cytoplasmic Ca^2+^ (She and Song, 2008) and the inhibition of PP2Cs because PP2C down regulates CDPK kinases by dephosphorylating them. Mutants of CDPKs lack Ca^2+^ induced SLAC1 activity (Mori et al., 2006) and are defective in Ca^2+^ and ABA induced stomatal closure (Kudla et al., 2010). These mutants further impair the activity of Ica (plasma membrane Ca^2+^ influx channel) through weakening of the activation of RbOH and, thereby, ROS production.

The calcineurin B-like calcium-binding protein (CBL) and CBL interacting protein kinase (CIPK) function as sensor molecules for decoding the information encoded by cellular Ca^2+^ signals. Unlike other kinases, CBL-CIPK binding forms a complex signalling structure combining Ca^2+^ binding activity in the CBL molecule and kinase activity in the CIPK molecule. CBLs are multi-localized proteins in the plasma membrane, vacuolar membrane, cytoplasm and the nucleus (Batistič et al., 2008), but CIPK proteins are mainly localized in the cytoplasm and nucleoplasm (Batistič et al., 2010). However, CBLs can target CIPKs to different cellular locations to perform different functions. Binding of Ca^2+^ bound CBL proteins to CIPK activates the kinase by releasing the auto-inhibitory domain from the kinase domain (Gong et al., 2002). Active tonoplast-localized CBL/CIPK24 complexes mediate the activity of vacuolar Ca^2+^/H^+^ antiporter CAX1 to maintain intracellular Ca^2+^ homeostasis and vacuolar ion transport mechanisms (Cheng et al., 2004). Some studies report the potential negative regulation of CIPKs by Ca^2+^ through CBL proteins (Tominaga et al., 2010). Therefore, the detailed mechanisms of regulation of CBL/CIPK under drought stress need further exploration. In addition to CBL proteins, protein–phosphatase (PP2C) mutually, and exclusively, interact within the C-terminus of CIPKs; this interaction may cause dephosphorylation of target proteins in the complex or direct dephosphorylation of CIPK (Weinl and Kudla, 2009).

In achieving timely coordination between each functional component for the required performance, there are several other regulatory mechanisms, which mediate the activities of the events discussed above. As shown in the yellow box in Fig. S1, phosphatidic acid (PA) and some species of long-chain base-1- phosphate such as sphingosine-1-phosphate (S1P), one of the most predominant sphingoid bases available in plants, promote ABA-induced stomatal closure (Guo and Wang, 2012). Sphingolipid signalling is very important to generate phosphatidic acid (PA), one of the crucial elements of the ABA signalling network. PA is involved in mediating all the functional sets of the ABA signalling described above through the production of ROS, inhibition of PP2C and inhibition of AHA1. Sphingosine kinase (SphK) is an enzyme that catalyses the production of sphingosine-1-phosphate (S1P) by phosphorylating sphingosine (SP) (Coursol et al., 2005) in response to an ABA signal, but the physiological function and the mode of regulation of SphK activation remains elusive (Guo and Wang, 2012). S1P physically binds to the guanine nucleotide binding site of G-protein α1 subunit (GPA1) stimulating the exchange of GTP for GDP. In general, phospholipase D (PLDα1) (PLD in Fig. S1) binds to GPA1 and reciprocally inhibits the activities of both proteins (GPA1 and PLD α1). If GPA1 is bound to GDP, the complex inhibits PLDα1 activity, but PLDα1 can inactivate the GPA1 protein by accelerating its GTPase activity. S1P may regulate GPA1 through the addition of GTP, permitting the release of PLDα1 to enhance the production of PA by catalysing the hydrolysis of phosphatidyl choline. As well as PLDα1, PLDδ also contributes to ABA signalling, which is regulated by nitric oxide (NO) (Distéfano et al., 2012), possibly through S-nitrosylation (Cys-residues) or nitration (Tyr-residues) of PLDδ. Recent studies suggest that [Ca^2+^]_cyt_ also activates PLDα1 (Jiang et al., 2013). In completing a reinforcing feedback loop PA, in turn, stimulates SphK activity by promoting its substrate binding capacity (Guo et al., 2012). This is a self-enhancing mechanism to facilitate the production of PA even at low concentration levels of SP. Interaction between PA and respiratory burst oxidase homologs (RbOH) regulates ABA-mediated ROS production (Zhang et al., 2011), which acts as a critical element in all aspects of the ABA signalling.

Figure S1 (pink box) depicts how ROS communicates with the downstream factors and how ROS production is regulated in guard cells. ROS controls guard cell osmoregulation via regulating GORK through post-translational modifications and, thereby, increasing the current intensity of the channel. In parallel, ROS modulate osmoregulation with post-translational modifications to redox-sensitive proteins (cysteine oxidation) such as MAPK, a protein kinase, which is an essential component for SLAC1 activation. Moreover, ROS regulates actin rearrangement via facilitating disassembly of actin filaments by weakening the inter-monomer bonds of the filaments. Guard cell Ca^2+^ signalling is also supported by ROS via initiating Ca^2+^ influxes to the cytosol by adjusting the gating properties of the plasma membrane Ica channels and further facilitating NO production to pump Ca^2+^ from the internal organelles. The importance of the activities discussed above highlights the essential nature of ROS signalling in ABA induced stomatal closure.

In the ABA signalling network, ROS are generated via two independent pathways that are regulated by respiratory burst oxidase homologues (RbOH) and copper amine oxidase (CuAO). There are two prominent respiratory burst oxidase homologues: RbOH-F and D, which are responsible for ROS production in plants under ABA regulation and share a functional redundancy. RbOH-F is regulated by SnRK2 (Kwak et al., 2003, Beguerisse-Díaz et al., 2012) through phosphorylation of Ser13 and Ser174 sites in the N-terminal region (Sirichandra et al., 2009) and synergistically by Ca^2+^ (Kimura et al., 2012). ROS production by RbOH-D is Ca^2+^-dependent (Ogasawara et al., 2008). Calcium activates RbOH-D either by inducing conformational changes or indirectly through activating CDPKs to phosphorylate the protein (Ogasawara et al., 2008). The current assumption about RbOH is that RbOH-D plays a more important role in plant–pathogen interactions while RbOH-F is more important in ABA signalling (Sirichandra et al., 2009). In addition to SnRK2 and CDPK, there are several important regulators of the activity of RbOH, such as the regulatory A subunit of protein phosphatase 2A (RCN1), PA, and phosphatidylinositol phosphates (PI3P and PI4P). PA physically binds to both RbOH homologues (Zhang et al., 2009) for activation. The literature shows that there is no physical binding between RbOH and PI3P/PI4P (Zhang et al., 2009). Therefore, the mode of regulation by PI3P/PI4P may be through the localization of cytoplasmic regulatory subunits of NADPH, as in animal cells (Ellson et al., 2006). The level of membrane-bound phospholipids PI4P in guard cells depends on the activity of phosphatidylinositol-4-OH kinases (PI4PK), which catalyze the production of PI4P by phosphorylating structural lipids, phosphatidylinositols, upon stimulation by ABA (Munnik and Nielsen, 2011). Similarly, PI3P production is catalyzed by phosphatidylinositol-3-OH kinase (PI3PK). PA may also stimulate the production of PI4P through PI4PK as PI4PK is identified as a target of PA (Hong et al., 2010). Inhibiting either the production of PI3P or/and PI4P, or blocking PI3P/PI4P binding domains, inhibits ABA induced stomatal closure. RCN1 is identified as an essential regulator of ROS production but activation of RCN1 in ABA signalling is not clear. Some literature suggests that RCN1 is a possible target of PA (Li et al., 2009). Further, ABA enhances ROS production through CuAO that catalyzes the oxidation of polyamines, such as putrescine, resulting in H_2_O_2_ (a type of ROS). Some literature reports that CuAO is a potential candidate for producing NO as a result of polyamine oxidation and that it could be mediated either by H_2_O_2_ or by an unknown mechanism (Wimalasekera et al., 2011). Both CuAO and RbOH are independently responsible for ABA induced ROS production (Trouverie et al., 2008).

Despite its beneficial regulations, ROS can cause cell damage if available in excess. Glutathione peroxidase 3 (GPX3) acts as a ROS scavenger in guard cells. There is evidence to suggest that ROS scavengers are activated by high ROS concentrations (Gutscher et al., 2009). However, some studies claim that the depletion of GPX3 does not show any significant effect on the increase in reactive oxygen species production in guard cells (Okuma et al., 2011) giving clues to the presence of other potential ROS scavenging pathways. According to the above findings, SnRK2, RCN1, PA, PI3P and PI4P (Kwak et al., 2006) are identified as essential regulators of the initiation of ROS production through regulation of RbOH. It is not clear that Ca^2+^ alone can produce ROS because phosphorylation is a prerequisite for Ca^2+^-dependent production of ROS. Based on the information available, we can suggest that SnRK2 activity may be the initial trigger for ABA induced ROS production and Ca^2+^ and CDPK may participate later in the process.

Cytosolic pH is a secondary messenger in guard cell ABA signalling. In open stomata, the cytosolic pH of guard cells ranges from 7.2 - 7.7; whereas pH varies from 7.4-7.9 in closed stomata (Hills et al., 2012). Some studies propose that cytosolic alkalization functions downstream of ROS production (Islam et al., 2010) as modulation of pH has no effect on the production of ROS (Gonugunta et al., 2009).

The coordinated activity of the signalling transduction mechanisms explained above leads guard cells to significantly reduce transpiration by half-closing the stomata within 5 to 10 min (Hedrich, 2012). However, the resulting closure should be maintained until plants are relieved of water stress because light dependent stomatal opening mechanisms may re-open the stomata when it is still harmful to do so. Maintenance of stomatal closure in the ABA signalling network is via the regulation of two influx systems in the guard cell plasma membrane to avoid accumulation of osmotic loads in the cytosol (Fig. S1 - ash colour box with dark green nodes). Significant ion channels deregulated during this process are the plasma membrane K^+^ influx channels and proton (H^+^) pumps (H^+^-ATPases). Maintenance of stomatal closure needs discontinuation of the activity of these two channels to further facilitate turgor reduction. During stomatal opening, the plasma membrane proton pumps mediate the H^+^ efflux from guard cells. The efflux of H^+^ ions from the cytoplasm causes plasma membrane hyperpolarization facilitating gating requirements for hyperpolarization activated K^+^ influx channel, KAT1. As a result of KAT1 activity, the level of K^+^ ions increase in the guard cell cytosol, altering the osmotic potential to a level that is favourable for attracting more water inside, ultimately leading to guard cell swelling. Plasma membrane H^+^-ATPases (AHA1) responsible for H^+^ efflux are sensitive to the blue light spectrum of natural daylight; hence down regulation of this protein phosphatase is an essential initial step for stomatal closure and it is carried out by phosphatidic acid (PA), which inhibits the phosphatase activity of PP1 (Protein phosphatase 1), the regulator of AHA1 (Takemiya and Shimazaki, 2010). Elevated cytosolic Ca^2+^ and cytosolic alkalization (pH) also act negatively on the activity of the proton pump where a reduction of pump activity enhances the depolarization of the plasma membrane in guard cells (Gonugunta et al., 2009) further facilitating the K^+^ efflux through GORK (plasma membrane K^+^ out channel). The plasma membrane hyperpolarization activated (-80 to -100 mV) inward rectifying K^+^ channel (KAT1) in guard cells plays a key role in stomatal opening by mediating K^+^ uptake. There are several other secondary K^+^ influx channels, such as KAT2, AKT1 and AKT2 available in the system but they only become active when KAT1 does not function well in the system. Deactivation of KAT1 is done by two protein kinases, SnRK2 and CDPK, by phosphorylating the channel by two independent pathways. Deactivation by SnRK2 is by phosphorylating the cytosolic C-terminal region (Thr306 and Thr308) (Sato et al., 2009) and CDPKs phosphorylate the KAT1 protein in a Ca^2+^ dependent manner, possibly at one site in the cytosolic N terminus (Thr45) and five sites in the cytosolic C-terminus (Thr308, Ser312, Ser589, Ser590 and Ser641) (Sato et al., 2010). Mutant plants lacking KAT1 show reduced amplitudes of inward K^+^ currents which correspond to a 38 – 45% lower stomatal opening (Kwak et al., 2001).

| (A)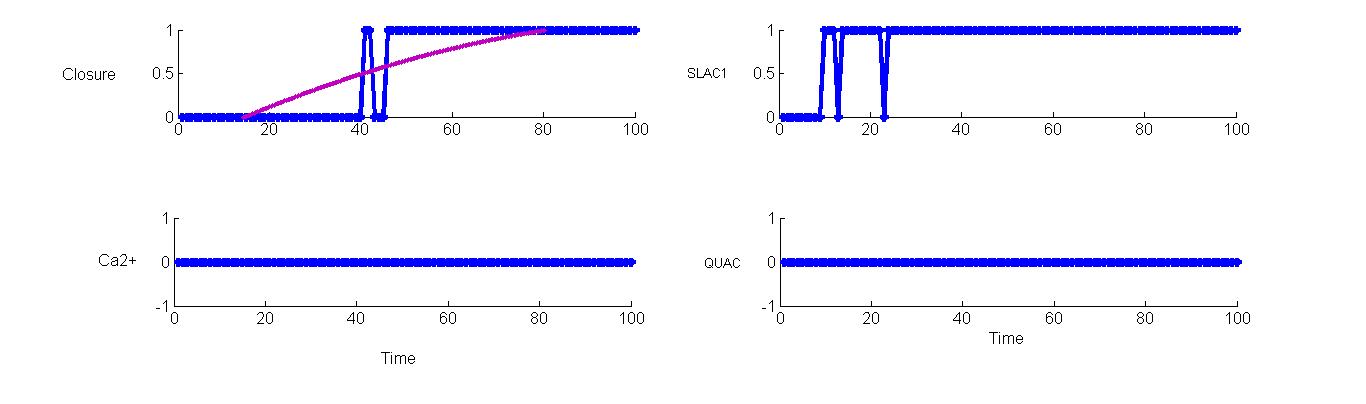 |
| --- |
| (B)  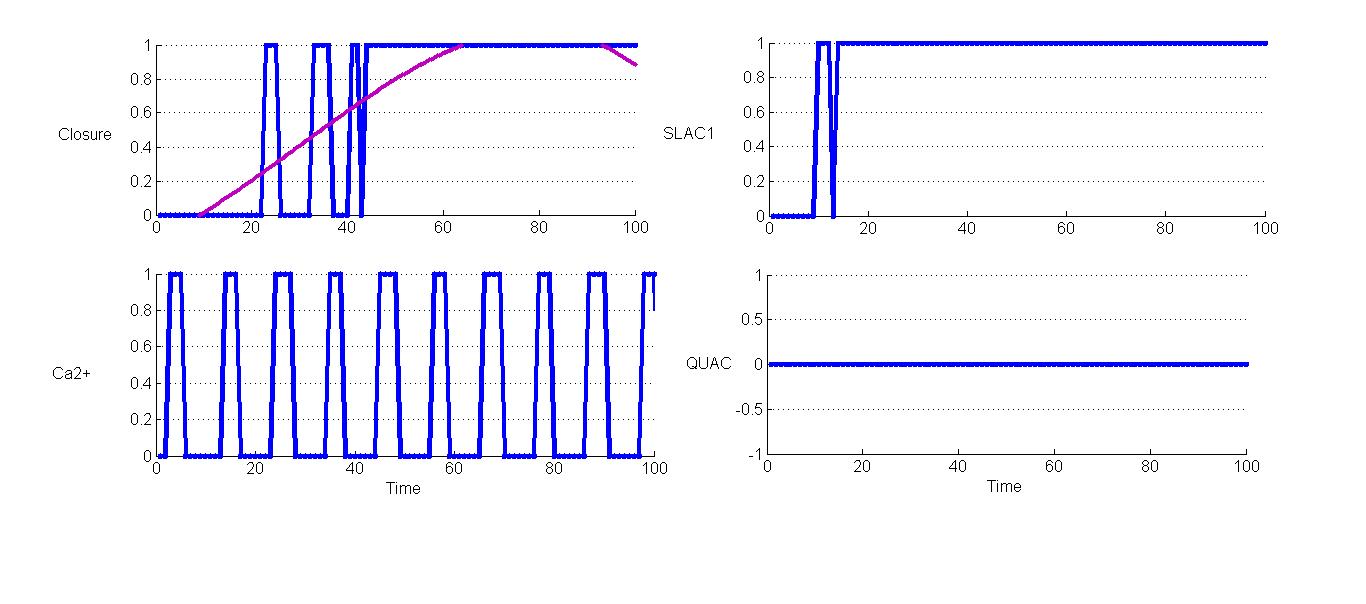 |

Figure S2: Importance of Ca^2+^ to the system when the QUAC channel is altered. Stomatal closure and SLAC1 induced by ABA (A) in a Ca^2+^ and QUAC knocked out system; and (B) in a QUAC perturbed system for a given initial condition


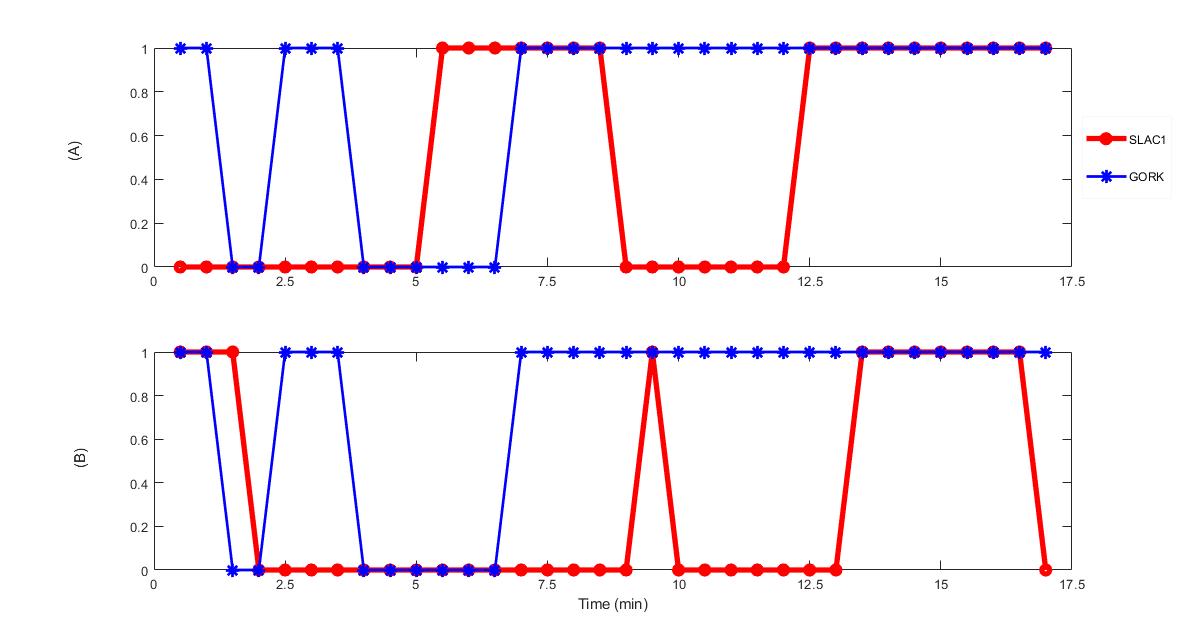


Figure S3: GORK activity is poorly disturbed in (A) QUAC and (B) S1P perturbations compared to SLAC1

Supplementary Table S1: Model interactions (edges), biological time and model time (newly added interactions are shown in bold letters)

| Interaction | | Regulation | Reference for Interaction | Boolean Function | Biological Half-time (sec) | Data Extraction Source | Model time |
| --- | --- | --- | --- | --- | --- | --- | --- |
| Target | Source |  |  |  |  |  |  |
| Sphingolipid Signalling | | |  |  |  |  |  |
| SK | ABA | Not clear | 18 | ABA or PA activates SK | 90 | ABA signalling ^19,20^ | 3 |
| **SK** | **PA** | Increase the specificity constant to promote substrate binding | 21 |  | 40 | ABA signalling (*in vivo*)^20^ | 2 |
| S1P | SK | Phosphorylation of Sphingosine to produce S1P | 22 | SK activates S1P | rapid | ABA signalling^22^ | 1 |
| GPA1 | S1P | Addition of GTP permitting PLD release | 19 | S1P activates GPA1 | <30 | S1P induced PLD activity reaches maximum in 1min in C6 glioma cells (*in vivo*)^23^. This agrees well with ABA → PLD time course in guard cells^18^ | 1 |
| PLD | GPA1 | Binding of GTP to GPA1 (Gα-GTP) dissociates GPA1 from PLD | 24 | GPA1 or NO or CA activates PLD | <30 |  | 1 |
| **PLD** | **NO** | May be S-nitrosylation of Cys residues or nitration of Tyr residues | 25 |  | 60 | Plants (*in vivo*)^26^ | 2 |
| **PLD** | **CA** | Strengthen enzyme-substrate binding/targeting membrane localization of proteins | 27 |  | <60 | Ca^2+^ activates maximum PLD activity in 3min in humans (*in* *vivo*)^28^ | 1 |
| PA | PLD | Facilitates the hydrolysis of P-choline to produce PA | 18 | PLD activates PA | Rapid | Considering the time frame of PA activation by ABA via several intermediary steps^24^ | 1 |
| **RCN1** | **PA** | Not clear | 29 | PA activates RCN1 | 60 | Assumed | 2 |
| Signal Perception via ABA | | |  |  |  |  |  |
| **PYR** | **ABA** | Physical binding (conformational changes) | 30 | ABA activates PYR | <10 | ABA signalling (*in vitro*)^31^ | 1 |
| **CuAO** | **ABA** | Induce biosynthesis | 32 | ABA activates CUAO | 150 | Peak activity of CuAO after adding ABA is 5 min (*in vivo*)^32^ | 5 |
| **PI4P** | **ABA** | Phosphoinositide turnover | 33 | ABA or PA activates PI4P | 100 | Generally PIP level peaked by 200 s in response to ABA in *Vicia faba*^33^ | 4 |
| **PI4P** | **PA** | Phosphoinositide turnover | 34 |  | 100 | Assumed same activation time scale as by ABA | 4 |
| **PI3P** | **ABA** | Phosphoinositide turnover | 33 | ABA activates PI3P | 100 | Assume same as PI4P | 4 |
| **PIP2** | **PI4P** | Phosphorylation | 33 | PI4P activates PIP2 | 2.5 | Both PIPs (PI4P) and PIP2 levels peaked by 200 s in response to ABA in *Vicia faba*^33^. Therefore, the time delay between PI4P to PIP2 is assumed to be rapid. | 1 |
| Inositol Signalling | | |  |  |  |  |  |
| PLC | ABA | Possibly via phosphorylation | 35 | ABA & CA  activates PLC | <30 | This was assumed based on ABA activation of InSP3, which occurs within 10 - 60s-time scale (assumption: PLC is needed for InSP3 synthesis)^33,36,37^ | 1 |
| PLC | CA | Ca^2+^ driven activity and membrane targeting | 38 |  | 30 | Assumed | 1 |
| InSP3 | PLC | PLC hydrolyse PIP2 to produce InSP3 | 33 | PLC & PIP2  activates InSP3 | 40 | Kinetic model for humans^39^ and various other sources | 2 |
| InSP3 | PIP2 |  | 33 |  | 40 |  | 2 |
| **InSP6** | **InSP3** | Phosphorylation by respective kinases | 40 | InSP3 & (no MRP5) activates InSP6 | 15 | This reaction is reported as rapid^36,41^ and the life time of InSP3 as 15s^39^ | 1 |
| **InSP6** | **MRP5** | Transport InSP6 to the vacuole | 42 |  | 138 | Maximum uptake of InSP6 is at 5.5 min in Arabidopsis^42^ | 5 |
| Osmoregulatory Enzymes/molecules | | |  |  |  |  |  |
| **pH** | **SnRK2** | Not clear | 43 | SnRK2 activates pH | 350 | Since pH increase is noticeable after 6 min in ABA signalling (*in vivo*)^44^ | 12 |
| **PP2C** | **PYR** | Physical binding (conformational changes) | 30 | (no PYR & no PA & no ROS & no ATGPX3 & (pH or ROP11))  or  (no PYR & no PA & no ROS & no ATGPX3 & ROP11)  or  no PYR  or  no PYR & ABH1  activates PP2C | 10 | ABA signalling (*in vitro*)^45^ | 1 |
| PP2C | PA | Decreases the phosphatase activity and/or membrane-tethering role | 46 |  | 10 | Based on general deactivation of protein phosphatases by lipids^47^ | 1 |
| PP2C | ROS | Oxidization | 48 |  | 360 | ABA signalling (*in vitro*)^48^ | 12 |
| **PP2C** | **GPX3** | Possibly by oxidation | 49 |  | 280 | Based on the time scale ROS ⟞ PP2C ^48,50^ | 11 |
| PP2C | pH | Regulating phosphatase activity | 51 |  | 60 | Assumed | 2 |
| **PP2C** | **ROP11** | Physically binds to protect from Receptor inhibition | 52 |  | 60 | Assumed | 2 |
| **PP2C** | **ABH1** | Not clear | 53 |  | 60 | Assumed | 2 |
| **ROP11** | **ERA1** | Changing localization | Assumed | ERA1 activates ROP11 | 60 | Assumed | 2 |
| **SnRK2** | **PP2C** | Dephosphorylation | 54 | PP2C deactivates SnRK2 | 10 | ABA signalling (*in vitro*)^55^ | 1 |
| **MAPK** | **ROS** | May be through post-translational modification of redox sensitive proteins (cysteine oxidation) | 56 | (ROS or (CDPK & CA)) & no PP2C activates MAPK | 150 | Human (*in vivo*)^57^ | 5 |
| **MAPK** | **CDPK** | Not Known | Assumed^58^ |  | 120 | This time scale is common for phosphorylation of MAPK in general^59^ | 4 |
| **MAPK** | **CA** | Not Known | Assumed^58^ |  | 120 |  | 4 |
| **MAPK** | **PP2C** | Dephosphorylation | 60 |  | 10 | Considering the time scale in which PP2C dephosphorylates SnRK2 | 1 |
| **CDPK** | **CA** | Ca^2+^ binding to the N-terminal lobe separates auto inhibitory domain from the active site | 61 | CA & no PP2C activates CDPK | 10 | Calculated based on Ca2+ dependant activation of SLAC1^5,62^ | 1 |
| **CDPK** | **PP2C** | Dephosphorylation | Assumed |  | 10 | Considering the time scale in which PP2C dephosphorylates SnRK2 | 1 |
| RbOH | SnRK2 | Phosphorylation | 63 | (SnRK2 or (CDPK & CA)) & RCN1 & PA & (PI3P or PI4P) & pH activates RbOH | 60 | Considering the time course of CDPK → RBOH because both SnRK2 and CDPK are kinases in the same family | 2 |
| **RbOH** | **CDPK** | Phosphorylation | 64 |  | 60 | In Arabidopsis (*in vivo*)^64^ |  |
| **RbOH** | **CA** | Ca^2+^ binds to EF-hand motif of RbOH but the significance of this binding to ROS production is not clear |  |  |  |  | 2 |
| **RbOH** | **RCN1** | Not clear | 65 |  | 60 | Assumed similar to other edge delays connected to RBOH with an ‘AND’ gate | 2 |
| **RbOH** | **PA** | Physically binds and stimulates | 66 |  | 60 |  | 2 |
| **RbOH** | **PI4P** | Not clear | 67 |  | 60 |  | 2 |
| **RbOH** | **PI3P** | Not clear | 67 |  | 60 |  | 2 |
| RbOH | pH | Not clear | 68 |  | 60 |  | 2 |
| **ROS** | **CuAO** | Catabolism of CuAO (oxidation) | 69 | ((CuAO or RBOH) & no ATGPX3)  or  (CuAO & RBOH)  activates ROS | 300 | In *Vicia faba* (*in vivo*)^32^ | 10 |
| ROS | RBOH | 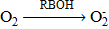(ROS) | 70 |  | <30 | Assumed rapid because in all experiments involving RBOH → ROS, RbOH activity is measured in terms of ROS production (refer the condition 3 below the table) | 1 |
| **ROS** | **GPX3** | 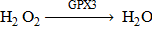 | 71 |  | 120 | In mitochondria (*in vitro*) ^72^ | 4 |
| **GPX3** | **ROS** | High levels of ROS lead the activation of the ROS scavenging system | Assumed | ROS activates GPX3 | 200 | Based on the time taken to oxidize redox regulated peroxidase in mammalian cells (*in* *vivo*)^73^ | 7 |
| NO Signalling | | |  |  |  |  |  |
| NO | ROS | Not clear | 74 | ROS & CaM activates NO | <10 | ABA signalling (*in* *vivo*)^75,76^ | 1 |
| **NO** | **CaM** | Ca^2+^- bound CaM interact NOS like Enzyme to produce NO | 77 |  | 60 | ABA signalling (*in vivo*)^78^ | 2 |
| **CADPR** | **CGMP** | 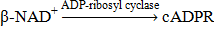  Activate ADP-ribosyl cyclase via G-kinase to stimulate cADPR synthesis | 79 | CGMP activates CADPR | <30 | Indirect calculation based on NO → Ca^2+^ (1.25min)^76^, cADPR → Ca^2+^ (40s)^80^ and NO → cGMP (20s)^81^ time frames because this regulation flow is as NO → cGMP → cADPR → Ca^2+^. | 1 |
| CGMP | NO | Activates soluble guanylyl cyclase resulting in cGMP by nitrosylation | 82 | NO activates CGMP | <30 | Human smooth muscle (*in vitro*)^81,83^ | 1 |
| Ion Channels | | |  |  |  |  |  |
| **SLAC1** | **SnRK2** | Phosphorylation | 4 | ((SnRK2 or (CDPK & no-PP2C)) & MAPK & (no-ERA1 or MRP5 or no-ABH1)) & no-MALATE activates SLAC1 | <30 | ABA signalling (*in vivo*)^84^ | 1 |
| **SLAC1** | **CDPK** | Phosphorylation | 4 |  | <30 | ABA signalling (*in vivo*) ^5,62^ | 1 |
| SLAC1 | PP2C | Dephosphorylation | 4 |  | 10 | Considering the time scale, in which PP2C dephosphorylates SnRK2 | 1 |
| **SLAC1** | **MAPK** | Not clear | 8 |  | <30 | Assumed the same time scale as for other activating kinases (assume equal to SnRK2/CDPK → SLAC1) | 1 |
| **SLAC1** | **ERA1** | Protein farnesylation | 85 |  | <30 | ABA signalling^85^ | 1 |
| **SLAC1** | **MRP5** | Not clear | 86 |  | 30 | Assumed | 1 |
| **SLAC1** | **Malate** | Increasing current noise | 87 |  | 10 | Assumed | 1 |
| GORK | NO | Nytrosylation | 88 | ((no  -NO or ROS or pH) & DEPOLAR) activates GORK | 120 | Guard cells^14^ | 4 |
| GORK | pH | Membrane delimited pathway | 11 |  | 0.45 | Guard cells (*in vivo*)^10^ | 1 |
| GORK | ROS | Post-translational modification increasing current intensity | 13 |  | 360 | ABA signalling (*in vivo*) ^89^ |  |
| GORK | DEPOLAR | Setting active potential for channel opening | 13 |  | <2 | Guard cells (*in vivo*)^90^ | 1 |
| **QUAC** | **SnRK2** | Phosphorylation | 91 | (SnRK2 & DEPOLAR) activates QUAC | 2 | Based on the time taken to activate SLAC1 by SnRK2 (both SLAC1 and QUAC are activated by phosphorylation) | 1 |
| **QUAC** | **DEPOLAR** | Voltage gated regulation | 92,93 |  | <1 | Guard cells^92,93^ | 1 |
| **ATALMT6** | **CA** | Ca^2+^-dependent current activation | 94 | CA activates ATALMT6 | 120 | Guard cells^94^ | 4 |
| **KAT1** | **CDPK** | Phosphorylation | 95 | SnRK2 or CDPK deactivates KAT1 | 1 | In *Xenopus laevis* oocytes^96^ | 1 |
| **KAT1** | **SnRK2** | Phosphorylation | 97 |  | 660 | ABA signalling^98^ | 33 |
| **QUAC** | **MALATE** | Malate is a substrate for R-type ion channels | Assumed | MALATE activates QUAC | <30 | Assumed rapid | 1 |
| TPK1 | CA | Conformational changes induced by binding into EF-hands | 99 | (CA & no-pH) activates TPK1 | <30 | Arabidopsis^15^ | 1 |
| TPK1 | pH | Not clear | 100 |  | 25 | Arabidopsis^101^ | 1 |
| Malate | PEPC | Catalyses the b-carboxylation of PEP to yield oxaloacetate (OAA) and inorganic phosphate (the branch-point step in the malate-accumulation pathway)  PEP(C_3_) → OAA(C_4_) → Malate | 102 | PEPC activates Malate | 130 | ABA signalling^103^ | 5 |
| **AHA1** | **PP1** | Through phosphorylation, displaces C-terminal auto inhibitory domain | 104 | (PP1 & no CA & no pH) activates AHA1 | <30 | ABA signalling^105^ | 1 |
| AHA1 | CA | Post-translational modulation | 106 |  | 30-60 | Guard cells (Fava Bean)^12^ | 2 |
| AHA1 | pH | Not clear | 107 |  | 20 | Plants^108^ | 1 |
| Calcium Signalling | | |  |  |  |  |  |
| **CA** | **Icas** | Influx of Ca^2+^ to cytosol | 109 | ((Icas or CADPR or Ica or InSP6) & no CaATPASE & no CAX ) activates CA | <30 | Guard cells^110^ | 1 |
| CA | CADPR | Influx of Ca^2+^ to cytosol | 80 |  | 40 | ABA signalling (*in vivo*) ^80^ | 2 |
| CA | Ica | Influx of Ca^2+^ to cytosol | 85 |  | <30 | Assumed rapid | 1 |
| CA | InSP6 | Influx of Ca^2+^ to cytosol | 111 |  | <1 | ABA signalling (*in vivo*) ^111^ | 1 |
| CA | Ca^2+^-ATPase | Efflux of Ca^2+^ from cytosol | 112 |  | 5 | CaATPase activation is immediately after Ca2+ binding to its transport site^112^ | 1 |
| **CA** | **CAX1** | Efflux of Ca^2+^ from cytosol | 113 |  | 100 | In Arabidopsis leaves ^113^ | 4 |
| **Ca^2+^-ATPase** | **CaM** | Suppress auto-inhibitory action by binding into N-terminus | 114 | CaM activates Ca^2+^-ATPase | 5 | Based on the time course from Ca^2+^ to Ca-ATPase ^112^ | 1 |
| **CaM** | **CA** | Ca^2+^ binding enhances catalytic activity | 114 | CA activates CaM | 5 | In Rat^115^ | 1 |
| Ica | ABH1 | Not known | 116 | ((no ABH1 or no ERA1 or MRP5) & ROS & no DEPOLAR) activates Ica | 30 | Assumed | 1 |
| Ica | ERA1 | May be through farnesylation | 85 |  | 30 |  | 1 |
| **Ica** | **MRP5** | Not Known | 86 |  | 30 |  | 1 |
| Ica | ROS | ROS modify Ica channel proteins through directly and/or through additional intermediate proteins | 117 |  | 120 | ABA signalling (*in vivo*) ^89^ | 4 |
| Ica | DEPOLAR | Changes in electrical potential | 117 |  | 18 | Guard cell (*in vivo*) (assume Ca2+ increase/decrease upon changing the polarity of the plasma membrane is due to Ica)^118^ | 1 |
| **CBL** | **CA** | Ca^2+^ binding for activation | 119 | CA activates CBL | <30 | Indirect calculation based on Ca2+ dependent inhibition of AKT1 channel^120^  [CBL → AKT1 10ms, Ca2+ → AKT1 115ms] | 1 |
| **CAX1** | **CIPK** | May mask auto-inhibitory domain | 121 | CIPK activates CAX1 | 12 | Indirect calculation based on Ca-CAX time course (~1.5min)^113^ | 1 |
| **SCAB1** | **ABA** | Not clear | 122 | ABA or InSP6 activates SCAB1 | 240 | As this is responsible for the bundling of actin filaments in ABA signalling, time taken to appear bundling is considered^123^ | 8 |
| **SCAB1** | **InSP6** | Not clear | 124 |  | 150 | ABA signalling^124^ | 5 |
| **Icas** | **ACTIN** | Stretch activation | 125 | ACTIN activates Icas | 1200 | In guard cells^125^ | 31 |
| **CIPK** | **CBL** | Masks auto-inhibitory domain and facilitate localization | 126 | CBL & no  PP2C activates CIPK | 78 | Plants^127^ | 3 |
| **CIPK** | **PP2C** | Replaces CBL protein to enhance auto inhibition | 128 |  | 30 | Considering the timescale in which PP2C dephosphorylates SnRK2 | 1 |
| **PP1** | **PA** | Inhibits the phosphatase activity | 55 | PA deactivates PP1 | 165 | ABA signalling ^129^ | 6 |
| **ABPS** | **CA** | Capping and depolymerization | 130 | (CA & no PI3P & no PI4P) activates ABPS | 42 | Indirect calculation | 2 |
| **ABPS** | **PI3P** | Inactivates actin stabilization and facilitates depolymerization | 131 |  | 360 | Assumed | 12 |
| **ABPS** | **PI4P** |  |  |  | 360 | Assumed | 12 |
| **ACTIN** | **ARP23** | Actin nucleation | 132 | ((ARP23 & SCAB1) & no AtRAC1 & (ROS or (CA & ABPS))) activates ACTIN | 150 | Human (*in vitro*) ^133^ | 5 |
| **ACTIN** | **SCAB1** | Stabilizes actin filaments | 122 |  | 180 | ABA signalling ^122^ | 6 |
| ACTIN | ATRAC1 | Not clear | 134 |  | 120 | ABA signalling^134^ | 4 |
| **ACTIN** | **ROS** | Depolymerizes actin ﬁlaments via weakening of inter-monomer bonds | 132 |  | 90 | G-actin in general (in vitro)^135^ | 3 |
| ACTIN | CA | Facilitates depolymerization | 136 |  | 90 | Arabidopsis^137^ | 3 |
| **ACTIN** | **ABPS** | Stabilizes actin filaments | 137 |  | 50 | Arabidopsis ^137^ | 2 |
| PEPC | Malate | Binding to inhibitory site (feedback inhibitor-binding site) | 102 | MALATE inhibits PEPC | 130 | ABA signalling^103^ | 5 |
| ATRAC1 | PP2C | Not clear | 134 | PP2C activates ATRAC1 | 60 | Assumed | 2 |
| **ARP23** | **PIP2** | PIP2 concentration may induce a ‘switch’ for N-WASP mediated Arp2/3 actin polymerization | 138 | (PIP2 or ROS) activates ARP23 | 110 | Calculated based on the time taken for nucleation of actin filaments in guard cells and was similar in human^123,139^ | 4 |
| **ARP23** | **ROS** | Not clear | 132 |  | 30 | Assumed | 1 |
| DEPOLAR | CA | Accumulation of positive ions makes membrane more positive | 140 | (CA or TPK1 or no AHA1 or SLAC1 or QUAC) activates DEPOLAR | <30 | ABA signalling^141^ | 1 |
| DEPOLAR | TPK1 | Addition of K^+^ to cytosol from the vacuole | Assumed |  | 0.001 | Common for all ion channels^142^ | 1 |
| DEPOLAR | AHA1 | Facilitates membrane hyperpolarization | 140 |  | 30 | Human^143^ | 1 |
| DEPOLAR | SLAC1 | Removal of negative charges makes membrane more positive | 144 |  | 0.004 | ABA signalling^145^ | 1 |
| **DEPOLAR** | **QUAC** | Removal of negative charges makes membrane more positive | 146 |  | 0.025 | Guard cells^146,147^ | 1 |
| CLOSURE | GORK | Removal of K^+^ (osmotic regulation) | 17 | (GORK & ACTIN & no MALATE & SLAC1) activates CLOSURE | 30 | Assumed | 1 |
| CLOSURE | ACTIN | Rearrangement of cytoskeleton | 148 |  |  |  | 1 |
| CLOSURE | Malate | Removal of malate (osmotic regulation) | 149 |  |  |  | 1 |
| CLOSURE | SLAC1 | Removal of Cl^-^ (osmotic regulation) | 150 |  |  |  | 1 |

Table S2: Comparison of model outputs in reference to activation time (min) from ABA treatment

| Node | Biological Time (min) | Model Time (min) |
| --- | --- | --- |
| Depolarization | 2^151^ | 1.5 - 3 |
| SLAC1 | 5 (t_1/2_ = 1.5) ^5^  2 ^151^ | 3.5 - 12 |
| S1P | 2 ^19^ | 1.5 - 2 |
| PYR | 0.5 - 1^31^ | 1 |
| PA | 2.5 - 5, 15 - 25 ^18^ | 3 - 4 |
| ROS | 2 (t_1/2_ = 1) ^141^  30 (t_1/2_ = 10)^152^  24 (t_1/2_ = 6)^153^  t_1/2_ = 5^154^  10 (t_1/2_ = 6)^66^ | 3 - 8 |
| pH | 12 (t_1/2_ = 6) ^44^  15 (t_1/2_ = 5)^152^  24 (t_1/2_ = 3)^153^ | 6 |
| Ca^2+^ | 10 - 20^155^  1 - 15^111^  5 ^156^ | 1 - 20 |
| PP2C | 3.3 (t_1/2_ = 10s) ^45^  2.5 (t_1/2_ = 30s) ^157^ | 2 |
| ACTIN | t_1/2_ = 6 ^148^  t_1/2_ = 3 ^148^ | 7 - 10 |
| KAT1 | t_1/2_ = 8 - 14 ^98^ | 4 - 10 |
| PIP2 | P1 - 10s P2 - 3 ^33^ | 2 |
| GORK | t_1/2_ = 2 ^158^ | 2 - 4 |
| SnRK2 | NA | 3 |

Table S3: Comparison of model outputs in reference to the behaviour of model mutants of selected nodes with biological evidence

| Node | Model mutant | Biological mutant |
| --- | --- | --- |
| Depolarization | - No stomatal closure due to alteration of GORK channel - Increase of Ca^2+^ | Induces a large rise in [Ca^2+^]_cyt_ ^5^ |
| SLAC1 | Stomatal insensitivity | Strong ABA insensitivity ^144^ |
| S1P | - Less sensitive to ABA | - Less sensitive to ABA ^20^ - 17-30% low PA production and 19% lower in S1P production ^159^ - Impairs ABA inhibition of PP2C activity^160^ |
| PYR | ABA insensitive | ABA insensitive (no stomatal closure)^161^ |
| PA | Greatly reduce ABA induce stomatal closure | ABA insensitive (no stomatal closure) ^46^ |
| ROS | Insensitive to ABA   - SLAC1 altered - ACTIN altered | Insensitive to ABA^162^ |
| pH | - Greatly reduce ABA induced closure - Changes the pattern of NO production and thereby the Ca^2+^ - Impairs ROS production | - Acidification of guard cell cytosol induces stomatal opening^140^ - Changes the patterns of NO production^140^ - Impairs ROS production^68^ |
| Ca^2+^ | - Slows down the stomatal closure | - Slows down the stomatal closure^111^ |
| PP2C | - Dominant positive perturbation makes stomata insensitive to ABA - Impair:   Ca^2+^ signalling  ROS production  SnRK2 activation  SLAC1 activation | - ABA insensitive (no stomatal closure) ^2^   - Impair:   Ca^2+^ signalling  ROS production  SnRK2 activation  SLAC1 activation |
| ACTIN | Stomata is insensitive to ABA | Disrupts both stomatal opening and closure ^163^ |
| KAT1 | No significant influence to ABA induced stomatal closure | Co-expression of multiple K^+^ in channels (KAT1, KAT2 and AKT1) potentially activated by the above mechanisms, result in functional redundancy of individual channels; hence, knocking out one channel may not have significant effect on stomatal action^98^. |
| PIP2 | No influence on ABA induced stomatal closure | No clear difference in stomatal conductance between WT and mutant plants^98^ |
| GORK | Stomata is ABA insensitive | Causes a loss of outward K^+^ current^164^ |
| SnRK2 | - ABA insensitive (no stomatal closure) - Impairs ROS production - Impairs Cytosolic alkalization | - ABA insensitive (no stomatal closure)^164^ - Impairs ROS production ^165^ - Impairs Cytosolic alkalization^43^ |
| PI3P  PI4P | - PI3P or PI4P alone do not show any sensitivity but stomata are nearly insensitive to ABA when both PI3P and PI4P are removed - Impair ROS production | - ABA insensitive (no stomatal closure) - Inhibits ROS generation^166^ |
| RCN1 | - Stomatal closure is drastically affected - Abolishes SLAC1 activity - Abolishes ROS production | - Insensitive to ABA (no stomatal closure)^65^ - Impairs activation of Anion channels^167^ - Impairs ROS production and [Ca^2+^]_cyt_ elevation ^66^ |
| QUAC | - Does not show any difference if malate metabolism path remains in the system but impairs closure if only malate transport is considered | - Impairs stomatal closure ^146^ - Reduces R-type current^146^ by 40% compared to the wild type^3^ |
| MAPK | - ABA insensitive (no stomatal closure) - Abolishes SLAC1 activity - Does not alter ROS production | - ABA insensitive (no stomatal closure)^56^ - Abolishes anion channel activity^56^ - Abolishes ABA induced ROS production ^168^ |
| ATALMT6 | - Does not have any influence on ABA sensitivity | - Similar to WT plants ^94^ |
| MRP5 | - Less sensitive to ABA (reduced probability of closure) - Impairs ABA activation of Ica channel | - Less sensitive to drought stress ^169^ - Impairs ABA activation of Ica channel ^86^ |
| AHA1 | - Overexpression makes stomata insensitive to ABA - Interferes with pattern of Ca^2+^ oscillation | - Overexpression makes stomata insensitive ^170^ - Interferes with Ca^2+^ oscillations ^171^ |
| PP1 | - Does not have any effect on the probability of closure | - Impairs phosphorylation of the H^+^-ATPase and thereby H^+^ pumping ^104^ |
| ACTIN | - Stomata are insensitive to ABA | - Disrupts both stomatal opening and closure^163^ |
| ARP2/3 | - Stomata are insensitive to ABA - Reduces Ca^2+^ oscillations | - Stomatal insensitivity ^172^ - Alter Ca^2+^ elevation^173^ |
| ATRAC1 | - Overexpression makes stomata insensitive to ABA - Disrupts Ca^2+^ increases in 50% of stomata | - Dominant positive mutants inhibit the ABA induced actin cytoskeleton and stomatal closure ^134^ |

Table S4: Mann-Whitney test results for the comparison of stomatal closure time in Ca^2+^ intact and Ca^2+^ independent systems

|  | |
| --- | --- |
|  | Closure Time |
| Mann-Whitney U | 114779.500 |
| Wilcoxon W | 240029.500 |
| Z | -2.263 |
| Asymp. Sig. (2-tailed) | .024 |

Grouping Variable: Ca^2+^ intact (Group 2) and Ca^2+^ independent (Group 1)

Table S5: Mann-Whitney test results for the comparison of SLAC1 activation time in Ca^2+^ intact and Ca^2+^ independent systems

| | SLAC1 activation time |  | | --- | --- | | Mann-Whitney U | 473.000 | | Wilcoxon W | 2751.000 | | Z | -7.989 | | Asymp. Sig. (2-tailed) | .000 | |  |
| --- | --- | --- | --- | --- | --- | --- | --- | --- | --- | --- | --- |
| Grouping Variable: Ca^2+^ intact (Group 2) and Ca^2+^ independent (Group 1) | |
|  |  |
|  |  |
|  |  |
|  |  |
|  |  |
|  |  |
|  |  |
|  |  |

## **Supplementary Text 1: The Boolean Model used in the Study**

SK = (ABA | PA)

S1P= (SK)

GPA1= (S1P)

PLD= (GPA1 | NO|CA)

GTG= (ABA & ¬ GPA1)

PA= (PLD | (PLC & PIP2))

RCN1= (PA)

CuAO= (ABA)

PI4P= (ABA|PA)

PI3P= (ABA)

PIP2= (PI4P)

PLC= (ABA & CA)

InSP3= (PIP2 & PLC)

InSP6= (InSP3 & ¬ MRP5)

PYR= (ABA)

pH=(SnRK2 & ¬ AHA1)

PP2C= (¬ PYR &¬ PA &¬ ROS &¬ ATGPX3 & (pH | ROP11))| (¬ PYR &¬ PA &¬ ROS &¬ ATGPX3 & ROP11)|¬ PYR| (¬ PYR & ABH1)

ROP11= (ERA1)

SnRK2= ( ¬ PP2C )

RBOH= (SnRK2 | (CDPK & CA)) & RCN1 & PA & (PI3P|PI4P) & pH

ROS= ((CuAO | RBOH) & ¬ ATGPX3)|(CuAO & RBOH)

ATGPX3=(ROS)

NO= (ROS & CaM)

CADPR= (CGMP)

CGMP= (NO)

Ica= ((¬ ABH1 |¬ ERA1| MRP5) & ROS & ¬ DEPOLAR )

SLAC1=((SnRK2|(CDPK & ¬ PP2C ))& MAPK & (¬ ERA1 | MRP5|¬ABH1))& ¬MALATE

MAPK= ( ROS|(CDPK & CA) ) & ¬ PP2C

CDPK= (CA & ¬ PP2C)

CA= ( (Icas | CADPR | Ica | InSP6) & ¬ CaATPASE & ¬ CAX )

CBL= (CA)

GORK= ((¬ NO | ROS| pH) & DEPOLAR)

CaATPASE= (CaM)

CaM= (CA)

QUAC= (SnRK2 & DEPOLAR)

CAX= (CIPK)

SCAB1= (ABA | InSP6)

Icas= (ACTIN)

CIPK= (CBL & ¬ PP2C)

TPK1= (CA & ¬ pH)

PP1= (¬ PA)

AHA1= (PP1 & ¬ CA & ¬ pH)

ABPS= (CA & ¬ PI3P & ¬ PI4P)

ACTIN= ((ARP23 & SCAB1) & ¬ AtRAC1 & (ROS|(CA & ABPS)))

PEPC= (¬ MALATE & ¬ ABA)

MALATE= (PEPC & ¬ QUAC & ¬ ATALMT6)

AtRAC1= (PP2C)

ARP23= (PIP2 | ROS)

DEPOLAR= (CA | TPK1 | ¬ AHA1 | SLAC1 | QUAC)

ATALMT6= (CA)

KAT1= (¬ CDPK & ¬SnRK2 & ¬ DEPOLAR)

CLOSURE= (GORK & ACTIN & ¬ MALATE & SLAC1)

## **Supplementary text 2:**

## **MATHLAB codes for Asynchronous Boolean Model**

function [ output_args ] = ABA_TOTAL_local_auto( input_args )

CL=zeros(51,100);

CAL=zeros(7,100);

z1=0;

z2=0;

for a=1:1

X=zeros(76,57);

Z= xlsread('Local half time.xlsx','Thalf_code');

m=randi([0,1],[1,57]);

%m=[1 0 0 1 1 0 0 0 0 0 0 1 1 1 0 1 1 0 0 1 0 1 0 0 0 0 0 0 0 1 1 0 0 1 1 1 1 0 1 0 1 1 0 0 0 1 0 0 1 1 0 0 0 1 1 1 0];

m(42)=1;

m(14)=1;

m(15)=0;

m(40)=0;

m(39)=1;

m(41)=1;

m(37)=1;

m(53)=0;

m(27)=0;

m(57)=0;

m(51)=0;

m(19)=0;

%m(45)=0;

m(2)=0;

m(11)=0;

Y=zeros(57,57);

X(1,:)=m;

for t=1:75

if t<=Z(2,4) && t<=Z(2,42)

m(2) =m(2);

elseif (t>Z(2,4) && t<=Z(2,42))

m(2)= X((t-Z(2,4)),4);%S1P

%m(2)=0;

elseif ( t> Z(2,42) && t> Z(2,4))

m(2)= X((t-Z(2,42)),42)| X((t-Z(2,4)),4);%S1P

%n(2)=0;

end

if t<=Z(4,46)

m(4)=m(4);

elseif ( t>Z(4,46) && t<=Z(4,20)&& t<=Z(4,27))

m(4)=X((t-Z(4,46)),46);%PA

elseif t>(Z(4,46) && t>Z(4,20)) && t<=Z(4,27)

m(4)=X((t-Z(4,46)),46)| X((t-Z(4,20)),20) ;%PA

elseif t>(Z(4,46) && t>Z(4,27) && t>Z(4,20))

m(4)=X((t-Z(4,46)),46)| X((t-Z(4,27)),27) | X((t-Z(4,20)),20);%PA

%n(4)=0;

end

if t<=Z(5,4)

m(5)=m(5);

elseif t>Z(5,4)

m(5)=X((t-Z(5,4)),4);%RCN1

%n(5)=0;

end

if (t<=Z(6,42))

m(6)=m(6);

elseif (t>Z(6,42)|| t>Z(6,4))

m(6)=X((t-Z(6,42)),42)|X((t-Z(6,4)),4);%PI4P

%n(6)=0;

end

if t<=Z(7,6)

m(7)=m(7);

elseif t> Z(7,6)

m(7)=X((t-Z(7,6)),6);%PIP2

%n(7)=0;

end

if ( t<=Z(8,42) && t<=Z(8,27))

m(8)=m(8);

elseif ( t>Z(8,42) && t>Z(8,27))

m(8)=X((t-Z(8,42)),42)& X((t-Z(8,27)),27);%PLC

%n(8)=0;

end

if ( t<=Z(9,7) && t<=Z(9,8))

m(9)=m(9);

elseif (t>Z(9,7) && t>Z(9,8))

m(9)=X((t-Z(9,7)),7)& X((t-Z(9,8)),8);%IP3

%n(9)=0;

end

if t<=Z(10,9)

m(10)=m(10);

elseif (t> Z(10,9) && t<= Z(10,45))

m(10)=X((t-Z(10,9)),9);%IP6

% n(10)=0;

elseif (t> Z(10,45))

m(10)=X((t-Z(10,9)),9)& ~X((t-Z(10,45)),45);%IP6

%m(10)=0;

end

if t> Z(11,42)

m(11)=X((t-Z(11,42)),42);%PYR

%n(11)=0;

end

if t<=Z(12,43)

m(12)=m(12);

elseif t> Z(12,43)

m(12)=X((t-Z(12,43)),43); %ROP11

%n(12)=0;

end

if t<= Z(13,15)

m(13)=m(13);

elseif t> Z(13,15)

m(13)=X((t-Z(13,15)),15); % pH

%n(13)=0;

end

if (t> Z(14,11)) && t<= Z(14,12)

m(14)=~X((t-Z(14,11)),11);%PP2C

elseif (t> Z(14,12) && t<= Z(14,19))

m(14)=( ~X((t-Z(14,11)),11) & ~X((t-Z(14,4)),4) & (X((t-Z(14,12)),12)| X((t-Z(14,13)),13)));%PP2C

elseif (t> Z(14,19) && t<= Z(14,18)) %n(14)=0;

m(14)=( ~X((t-Z(14,11)),11) & ~X((t-Z(14,4)),4) & ~X((t-Z(14,19)),19)& (X((t-Z(14,12)),12)| X((t-Z(14,13)),13)));%PP2C

elseif ( t> Z(14,18) )

m(14)=(~X((t-Z(14,11)),11) & ~X((t-Z(14,4)),4) & ~X((t-Z(14,18)),18) & ~X((t-Z(14,19)),19) & (X((t-Z(14,12)),12)| X((t-Z(14,13)),13)))|(~X((t-Z(14,11)),11) & ~X((t-Z(14,4)),4) & ~X((t-Z(14,18)),18)& ~X((t-Z(14,19)),19)& (X((t-Z(14,12)),12) | X((t-Z(14,44)),44)));%PP2C

end

if t> Z(15,14)

m(15)=~X((t-Z(15,14)),14);%SnRK2

%n(15)=0;

end

if ( t<=Z(16,4))

m(16)=m(16); %RBOH

%n(16)=0;

elseif (t>Z(16,4))

m(16)=((X((t-Z(16,15)),15) & X((t-Z(16,5)),5) & X((t-Z(16,4)),4) & (X((t-Z(16,6)),6) | X((t-Z(16,48)),48)) & X((t-Z(16,13)),13)))|( X((t-Z(16,27)),27) & X((t-Z(16,26)),26) & X((t-Z(16,5)),5) & X((t-Z(16,4)),4) & (X((t-Z(16,6)),6)|X((t-Z(16,48)),48))& X((t-Z(16,13)),13)); %RBOH

%n(16)=0;

end

if t<= Z(17,42)

m(17)=m(17);

elseif t> Z(17,42)

m(17)= X((t-Z(17,42)),42); % CuAO

%n(17)=0;

end

if (t> Z(18,16) && t<=Z(18,19))

m(18)= X((t-Z(18,16)),16) ; %ROS

elseif (t> Z(18,16) && t> Z(18,19) && t<= Z(18,17))

m(18)=(X((t-Z(18,16)),16) & ~X((t-Z(18,19)),19)); %ROS

%n(18)=0;

elseif (t> Z(18,17))

m(18)=((X((t-Z(18,16)),16)| X((t-Z(18,17)),17))& ~X((t-Z(18,19)),19))|(X((t-Z(18,17)),17)& X((t-Z(18,16)),16)); %ROS

%n(18)=0;

end

%{

if t> 0

m(18)=0;%ROS

end

%}

if t<= Z(19,18)

m(19)=m(19);

elseif t> Z(19,18)

m(19)=X((t-Z(19,18)),18); % ATGPX3

%n(19)=0;

end

if (t<=Z(20,49))

m(20)=m(20);

elseif (t> Z(20,18) && t>Z(20,49))

m(20)=X((t-Z(20,18)),18)& X((t-Z(20,49)),49); %NO

%n(20)=0;

end

if t>Z(21,22)

m(21)=X((t-Z(21,22)),22); %cADPR

end

%{

if t> 0

m(21)=0;%cADPR

end

%}

if t> Z(22,20)

m(22)=X((t-Z(22,20)),20); %cGMP

%n(22)=0;

end

if (t>(Z(23,44)|| Z(23,43)|| Z(23,45)) && t> Z(23,18) && t> Z(23,40))

m(23)=((~X((t-Z(23,44)),44)|~X((t-Z(23,43)),43)|X((t-Z(23,45)),45))& (X((t-Z(23,18)),18)& ~X((t-Z(23,40)),40)));%Ica

%n(23)=0;

end

if t<=Z(24,15)

m(24)=m(24);

elseif (t> Z(24,15) && t> Z(24,26) && t> Z(24,14) && t> Z(24,25) && t> Z(24,45) && t> Z(24,43) && t> Z(24,44) )

m(24)=(X((t-Z(24,15)),15) & X((t-Z(24,25)),25) & (X((t-Z(24,45)),45)| ~X((t-Z(24,43)),43)| ~X((t-Z(24,44)),44)))| ( X((t-Z(24,26)),26)& ~X((t-Z(24,14)),14) & X((t-Z(24,25)),25) & (X((t-Z(24,45)),45)| ~X((t-Z(24,43)),43)| ~X((t-Z(24,44)),44)) );%SLAC1

end

if (t<=Z(25,26))

m(25)=m(25); % MAPK

%n(25)=0;

elseif (t>Z(25,26))

m(25)=((X((t-Z(25,18)),18)| (X((t-Z(25,26)),26)& X((t-Z(25,27)),27)))& ~X((t-Z(25,14)),14)); % MAPK

end

if ( t> Z(26,27) && t>Z(26,14))

m(26)= X((t-Z(26,27)),27)& ~X((t-Z(26,14)),14); % CDPK

%n(26)=0;

end

if ( t> Z(27,10) && t<= Z(27,21) && t<= Z(27,33) )

m(27)=((X((t-Z(27,10)),10)|X((t-Z(27,23)),23)) ); %CA

elseif ( t> Z(27,10) && t> Z(27,21) && t<= Z(27,33) )

m(27)=((X((t-Z(27,21)),21)|X((t-Z(27,10)),10)|X((t-Z(27,23)),23)| X((t-Z(27,51)),51)) & ~X((t-Z(27,31)),31)); %CA

elseif ( t> Z(27,10) && t> Z(27,21) && t> Z(27,33) )

m(27)=((X((t-Z(27,21)),21)|X((t-Z(27,10)),10)|X((t-Z(27,23)),23)| X((t-Z(27,51)),51)) & ~X((t-Z(27,31)),31)& ~ X((t-Z(27,33)),33)); %CA

end

%{

elseif t>30

m(27)=0;

end

%}

if t> Z(28,27)

m(28)= X((t-Z(28,27)),27); % CBL

% n(28)=0;

end

if ( t> Z(29,27) && t>Z(29,40))

m(29)= X((t-Z(29,27)),27) & X((t-Z(29,40)),40);% TPC1

% n(29)=0;

end

if ( t> Z(30,13) && t> Z(30,40) && t<=Z(30,18))

m(30)=X((t-Z(30,13)),13)& X((t-Z(30,40)),40); % GORK ( i removed NO from the Boolean function

%n(30)=0;

%elseif (t> Z(30,20) && t>Z(30,13) && t>Z(30,40)&& t<=Z(30,18))

%m(30)=((~X((t-Z(30,20)),20)| X((t-Z(30,13)),13))& X((t-Z(30,40)),40)); % GORK

elseif (t>Z(30,13) && t>Z(30,40) && t>Z(30,18) )

m(30)=(( X((t-Z(30,18)),18)| X((t-Z(30,13)),13))& X((t-Z(30,40)),40)); % GORK

end

if t> Z(31,49)

m(31)=X((t-Z(31,49)),49); % CaATPase

%n(31)=0;

end

if (t>Z(32,40))

c1=(X((t-Z(32,40)),40)& X((t-Z(32,15)),15));

c2=( X((t-Z(32,39)),39) & X((t-Z(32,15)),15) & ~X((t-Z(32,40)),40));

m(32)= c1|c2 ; %QUAC

%n(32)=0;

end

if t> Z(33,34)

m(33)=X((t-Z(33,34)),34); % CAX

%n(33)=0;

end

if (t> Z(34,28) && t> Z(34,14))

m(34)= X((t-Z(34,28)),28)& ~ X((t-Z(34,14)),14); %CIPK

%n(34)=0;

end

if t> Z(35,27) && Z(35,13)

m(35)=X((t-Z(35,27)),27)& ~X((t-Z(35,13)),13); % TPK

%n(35)=0;

end

if t<= Z(36,4)

m(36)=m(36);

elseif t> Z(36,4)

m(36)=~X((t-Z(36,4)),4); % PP1

end

if ( t> Z(37,36) && t<= Z(37,27))

m(37)=m(37);

elseif ( t> Z(37,36) && t> Z(37,27) && t> Z(37,13))

m(37)=( X((t-Z(37,36)),36)& ~X((t-Z(37,27)),27)& ~X((t-Z(37,13)),13)); % AHA1

%n(37)=0;

end

if t> Z(38,39)

m(38)= ~X((t-Z(38,39)),39);%PEPC

%n(38)=0;

end

if ( t>Z(39,32) && t>Z(39,56) && t<=Z(39,38))

m(39)= ~X((t-Z(39,32)),32) & ~X((t-Z(39,56)),56);%MALATE

%n(39)=0;

elseif (t> Z(39,38) && t>Z(39,32) && t>Z(39,56))

m(39)= X((t-Z(39,38)),38) & ~X((t-Z(39,32)),32) & ~X((t-Z(39,56)),56);%MALATE

%n(39)=0;

end

if t> (Z(40,27) || Z(40,35) || Z(40,37) || Z(40,32) || Z(40,24))

m(40)=(X((t-Z(40,27)),27)|X((t-Z(40,35)),35)| X((t-Z(40,32)),32)| X((t-Z(40,24)),24))& ~ X((t-Z(40,37)),37);% DEPOLAR

%n(40)=0;

end

if ( t> Z(41,40) && t<= Z(41,15))

m(41)= ~X((t-Z(41,40)),40) & ~X((t-Z(41,26)),26);

elseif ( t> Z(41,40) && t> Z(41,15) && t>Z(41,26))

m(41)= ~X((t-Z(41,40)),40)& ~X((t-Z(41,15)),15) & ~X((t-Z(41,26)),26);% KAT1

% n(41)=0;

end

if t> Z(46,2)

m(46)= X((t-Z(46,2)),2);% GPA1

%n(46)=0;

end

if (t> Z(47,42) && t>Z(47,46))

m(47)= X((t-Z(47,42)),42)& ~X((t-Z(47,46)),46); % GTG

%n(47)=0;

end

if t> Z(48,42)

m(48)=X((t-Z(48,42)),42);% pi3p

%n(48)=0;

end

if t> Z(49,27)

m(49)=X((t-Z(49,27)),27);%CaM

%n(49)=0;

end

if t> Z(50,10) && t<=Z(50,42)

m(50)=X((t-Z(50,10)),10) ; % scab1

%n(50)=0;

elseif (t> Z(50,10) && t>Z(50,42) )

m(50)=X((t-Z(50,10)),10) | X((t-Z(50,42)),42); % scab1

end

if t<= Z(51,53)

m(51)=m(51);

elseif t> Z(51,53)

m(51)=X((t-Z(51,53)),53) ;%Icas

end

%{

if t> 0

%Icas

m(51)=0;

end

%}

if ( t> Z(52,27) && t<=Z(52,6))

m(52)=X((t-Z(52,27)),27); %ABPS

%n(52)=0;

elseif ( t> Z(52,27) && t>Z(52,6))

m(52)=X((t-Z(52,27)),27)& ~X((t-Z(52,48)),48)& ~X((t-Z(52,6)),6); %ABPS

end

if ( t> Z(53,55) && t>Z(53,50) && t>Z(53,54) && t>Z(53,27) && t>Z(53,52) && t> Z(53,18))

m(53)=((X((t-Z(53,55)),55)| X((t-Z(53,50)),50))& ~X((t-Z(53,54)),54) & ((X((t-Z(53,27)),27)|X((t-Z(53,18)),18)))); % ACTIN

%n(53)=0;

end

if t> Z(54,14)

m(54)=X((t-Z(54,14)),14);%atrAC1

%n(54)=0;

end

if t> Z(55,18)&& t<=Z(55,7)

m(55)= X((t-Z(55,18)),18); %ARP23

%n(55)=0;

elseif (t>Z(55,7) && t> Z(55,18) )

m(55)=X((t-Z(55,7)),7)|X((t-Z(55,18)),18); %ARP23

end

if t> Z(56,27)

m(56)=X((t-Z(56,27)),27); % ATALMT6

%n(56)=0;

end

if t> (Z(57,30) && Z(57,41) && Z(57,53) && Z(57,24) && Z(57,39))

m(57)=((X((t-Z(57,30)),30)& X((t-Z(57,53)),53) & X((t-Z(57,24)),24) & ~X((t-Z(57,39)),39)));%CLOSURE

end

%{

if t<30

m(42)=1;

elseif t>30

m(42)=0;

end

%}

X(t+1,:)=m;

end

display(X)

nmax=length(X(1,:));

kmax=length(X(:,1));

attractor=0;

for c=1 : kmax

for d= 2:kmax

IDNodes=length(find(X(c,:)==X(d,:)));

if (IDNodes == nmax)

attractor=1;

break;

end

end

if (attractor==1)

for p=d:c

attrStates(p-d+1,:)=X(p,:);

end

attrLength=c-d;

else

attrStates=[];

attrLength=inf;

end

end

for r=1:length(attrStates(:,1))

for j=2:length(attrStates(:,1))

if attrStates(r,:)==attrStates(j,:)

break;

end

end

end

attrStates (j:end,:) =[];

%display(attrLength);

%display(attrStates);

%[b,m,n] = unique(attrStates,'rows');

display(attrStates)

display(c)

display(d)

%display(Y)

xlswrite('Asyn_2.xlsx', X,'X35');

xlswrite('Asyn_2.xlsx', attrStates,'A35');

%CL(:,a)=X(:,57);

%CAL(:,a)=X(45:51,27);

end

SK=X(:,1);

S1P=X(:,2);

PLD=X(:,3);

PA=X(:,4);

RCN1=X(:,5);

PI4P=X(:,6);

PIP2=X(:,7);

PLC=X(:,8);

InSP3=X(:,9);

InSP6=X(:,10);

PYR=X(:,11);

ROP11=X(:,12);

Ph=X(:,13);

PP2C=X(:,14);

SnRK2=X(:,15);

RBOH=X(:,16);

CuAO=X(:,17);

ROS=X(:,18);

ATGPX3=X(:,19);

NO=X(:,20);

CADPR=X(:,21);

CGMP=X(:,22);

Ica=X(:,23);

SLAC1=X(:,24);

MAPK=X(:,25);

CDPK=X(:,26);

CA=X(:,27);

CBL=X(:,28);

TPC1=X(:,29);

GORK=X(:,30);

CaATPASE=X(:,31);

QUAC=X(:,32);

CAX=X(:,33);

CIPK=X(:,34);

TPK=X(:,35);

PP1=X(:,36);

AHA1=X(:,37);

PEPC=X(:,38);

MALATE=X(:,39);

DEPOLAR=X(:,40);

KAT1=X(:,41);

ABA=X(:,42);

ERA1=X(:,43);

ABH1=X(:,44);

MRP5=X(:,45);

GPA1=X(:,46);

GTG = X(:,47);

PI3P =X(:,48);

CaM =X(:,49);

SCAB1=X(:,50);

Icas =X(:,51);

ABPS = X(:,52);

ACTIN = X(:,53);

AtRAC1=X(:,54);

ARP23 = X(:,55);

ATALMT6 = X(:,56);

CLOSURE=X(:,57);

%display(CL)

%{

CLX=CL(42:51,:);

for g=1:100;

osci=sum(CAL(:,g));

if CLX(:,g)==1;

z1=z1+1;

end

if osci==3;

z2=z2+1;

end

end

%display(CAL)

%display(z1)

%display(z2)

%}

figure(1);

subplot(3,2,1); plot(1:length(X(:,1)),CLOSURE,'color','black','LineWidth',3)

title('CLOSURE')

subplot(3,2,2);plot(1:length(X(:,1)),SLAC1,'color','green','LineWidth',3)

title('SLAC1')

subplot(3,2,3);plot(1:length(X(:,1)),CA,'color','red','LineWidth',3)

title('Ca')

subplot(3,2,4);plot(1:length(X(:,1)),GORK,'color','blue','LineWidth',3)

title('GORK')

subplot(3,2,5);plot(1:length(X(:,1)),ACTIN,'color','c','LineWidth',3)

title('ACTIN')

subplot(3,2,6);plot(1:length(X(:,1)),MALATE,'color','magenta','LineWidth',3)

title('MALATE')

1. Umezawa, T. *et al.* Type 2C protein phosphatases directly regulate abscisic acid-activated protein kinases in Arabidopsis. *Proc. Natl. Acad. Sci.* **106,** 17588–17593 (2009).

2. Umezawa, T. *et al.* Molecular basis of the core regulatory network in ABA responses: sensing, signaling and transport. *Plant cell Physiol.* **51,** 1821–1839 (2010).

3. Barbier-Brygoo, H. *et al.* Anion channels/transporters in plants: from molecular bases to regulatory networks. *Annu. Rev. Plant Biol.* **62,** 25–51 (2011).

4. Brandt, B. *et al.* Reconstitution of abscisic acid activation of SLAC1 anion channel by CPK6 and OST1 kinases and branched ABI1 PP2C phosphatase action. *Proc. Natl. Acad. Sci.* **109,** 10593–10598 (2012).

5. Marten, H., Konrad, K. R., Dietrich, P., Roelfsema, M. R. G. & Hedrich, R. Ca2+-Dependent and -Independent Abscisic Acid Activation of Plasma Membrane Anion Channels in Guard Cells of Nicotiana tabacum. *Plant Physiol.* **143,** 28–37 (2007).

6. Liu, Y. Roles of mitogen-activated protein kinase cascades in ABA signaling. *Plant Cell Rep.* **31,** 1–12 (2012).

7. Danquah, A., de Zelicourt, A., Colcombet, J. & Hirt, H. The role of ABA and MAPK signaling pathways in plant abiotic stress responses. *Biotechnol. Adv.* **32,** 40–52 (2013).

8. Pitzschke, A. & Hirt, H. Disentangling the complexity of mitogen-activated protein kinases and reactive oxygen species signaling. *Plant Physiol.* **149,** 606–615 (2009).

9. Dreyer, I. & Blatt, M. R. What makes a gate? The ins and outs of Kv-like K+ channels in plants. *Trends Plant Sci.* **14,** 383–390 (2009).

10. Fan, L.-M., Wang, Y.-F. & Wu, W.-H. Outward K+ channels in Brassica chinensis pollen protoplasts are regulated by external and internal pH. *Protoplasma* **220,** 143–152 (2003).

11. Pandey, S., Zhang, W. & Assmann, S. M. Roles of ion channels and transporters in guard cell signal transduction. *FEBS Lett.* **581,** 2325–2336 (2007).

12. Tran, D. *et al.* Post‐transcriptional regulation of GORK channels by superoxide anion contributes to increases in outward‐rectifying K+ currents. *New Phytol.* **198,** 1039–1048 (2013).

13. Dreyer, I. & Uozumi, N. Potassium channels in plant cells. *FEBS J.* **278,** 4293–4303 (2011).

14. Sokolovski, S. & Blatt, M. R. Nitric oxide block of outward-rectifying K+ channels indicates direct control by protein nitrosylation in guard cells. *Plant Physiol.* **136,** 4275 (2004).

15. Czempinski, K., Zimmermann, S., Ehrhardt, T. & Müller-Röber, B. New structure and function in plant K+ channels: KCO1, an outward rectifier with a steep Ca2+ dependency. *EMBO J.* **16,** 2565–2575 (1997).

16. Gobert, A., Isayenkov, S., Voelker, C., Czempinski, K. & Maathuis, F. J. M. The two-pore channel TPK1 gene encodes the vacuolar K+ conductance and plays a role in K+ homeostasis. *Proc. Natl. Acad. Sci.* **104,** 10726–10731 (2007).

17. Hosy, E. *et al.* The Arabidopsis outward K+ channel GORK is involved in regulation of stomatal movements and plant transpiration. *Proc. Natl. Acad. Sci.* **100,** 5549–5554 (2003).

18. Jacob, T., Ritchie, S., Assmann, S. M. & Gilroy, S. Abscisic acid signal transduction in guard cells is mediated by phospholipase D activity. *Proc. Natl. Acad. Sci.* **96,** 12192–12197 (1999).

19. Coursol, S. *et al.* Sphingolipid signalling in Arabidopsis guard cells involves heterotrimeric G proteins. *Nature* **423,** 651–654 (2003).

20. Guo, L., Mishra, G., Taylor, K. & Wang, X. Phosphatidic acid binds and stimulates Arabidopsis sphingosine kinases. *J. Biol. Chem.* **286,** 13336–13345 (2011).

21. Guo, L. & Wang, X. Crosstalk between phospholipase D and sphingosine kinase in plant stress signaling. *Front. Plant Sci.* **3,** (2012).

22. Coursol, S. *et al.* Arabidopsis sphingosine kinase and the effects of phytosphingosine-1-phosphate on stomatal aperture. *Plant Physiol.* **137,** 724–737 (2005).

23. Sato, K., Ui, M. & Okajima, F. Differential roles of Edg-1 and Edg-5, sphingosine 1-phosphate receptors, in the signaling pathways in C6 glioma cells. *Mol. Brain Res.* **85,** 151–160 (2000).

24. Guo, L. *et al.* Connections between Sphingosine Kinase and Phospholipase D in the Abscisic Acid Signaling Pathway in Arabidopsis. *J. Biol. Chem.* (2012).

25. Distéfano, A. M., Scuffi, D., García-Mata, C., Lamattina, L. & Laxalt, A. M. Phospholipase Dδ is involved in nitric oxide-induced stomatal closure. *Planta* 1–9 (2012).

26. Lanteri, M. L., Laxalt, A. M. & Lamattina, L. Nitric Oxide Triggers Phosphatidic Acid Accumulation via Phospholipase D during Auxin-Induced Adventitious Root Formation in Cucumber. *Plant Physiol.* **147,** 188–198 (2008).

27. Qin, C. & Wang, X. The Arabidopsis Phospholipase D Family. Characterization of a Calcium-Independent and Phosphatidylcholine-Selective PLDζ1 with Distinct Regulatory Domains. *Plant Physiol.* **128,** 1057–1068 (2002).

28. Kessels, G. C., Roos, D. & Verhoeven, A. J. fMet-Leu-Phe-induced activation of phospholipase D in human neutrophils. Dependence on changes in cytosolic free Ca2+ concentration and relation with respiratory burst activation. *J. Biol. Chem.* **266,** 23152–23156 (1991).

29. Li, M., Hong, Y. & Wang, X. Phospholipase D-and phosphatidic acid-mediated signaling in plants. *Biochim. Biophys. Acta (BBA)-Molecular Cell Biol. Lipids* **1791,** 927–935 (2009).

30. Cutler, S. R., Rodriguez, P. L., Finkelstein, R. R. & Abrams, S. R. Abscisic acid: emergence of a core signaling network. *Annu. Rev. Plant Biol.* **61,** 651–679 (2010).

31. Hao, Q. *et al.* The Molecular Basis of ABA-Independent Inhibition of PP2Cs by a Subclass of PYL Proteins. *Mol. Cell* **42,** 662–672 (2011).

32. An, Z., Jing, W., Liu, Y. & Zhang, W. Hydrogen peroxide generated by copper amine oxidase is involved in abscisic acid-induced stomatal closure in Vicia faba. *J. Exp. Bot.* **59,** 815–825 (2008).

33. Lee, Y. *et al.* Abscisic acid-induced phosphoinositide turnover in guard cell protoplasts of Vicia faba. *Plant Physiol.* **110,** 987–996 (1996).

34. Stevenson, J. M., Perera, I. Y. & Boss, W. F. A phosphatidylinositol 4-kinase pleckstrin homology domain that binds phosphatidylinositol 4-monophosphate. *J. Biol. Chem.* **273,** 22761–22767 (1998).

35. Webb, A. A. R. & Robertson, F. C. Calcium Signals in the Control of Stomatal Movements. *Coding Decod. Calcium Signals Plants* 63–77 (2011).

36. Munnik, T. & Vermeer, J. E. M. Osmotic stress‐induced phosphoinositide and inositol phosphate signalling in plants. *Plant. Cell Environ.* **33,** 655–669 (2010).

37. Perera, I. Y., Hung, C.-Y., Moore, C. D., Stevenson-Paulik, J. & Boss, W. F. Transgenic Arabidopsis Plants Expressing the Type 1 Inositol 5-Phosphatase Exhibit Increased Drought Tolerance and Altered Abscisic Acid Signaling. *Plant Cell Online* **20,** 2876–2893 (2008).

38. Munnik, T. in *Phospholipases in Plant Signaling* 27–54 (Springer, 2014).

39. Suh, B.-C. & Hille, B. Does diacylglycerol regulate KCNQ channels? *Pflügers Arch. Eur. J. Physiol.* **453,** 293–301 (2006).

40. Stevenson-Paulik, J., Odom, A. R. & York, J. D. Molecular and biochemical characterization of two plant inositol polyphosphate 6-/3-/5-kinases. *J. Biol. Chem.* **277,** 42711–42718 (2002).

41. Michell, R. H., Perera, N. M. & Dove, S. K. New insights into the roles of phosphoinositides and inositol polyphosphates in yeast. *Biochem. Soc. Trans.* **31,** 11–15 (2003).

42. Nagy, R. *et al.* The Arabidopsis ATP-binding cassette protein AtMRP5/AtABCC5 is a high affinity inositol hexakisphosphate transporter involved in guard cell signaling and phytate storage. *J. Biol. Chem.* **284,** 33614–33622 (2009).

43. Islam, M. M. *et al.* Cytosolic Alkalization and Cytosolic Calcium Oscillation in Arabidopsis Guard Cells Response to ABA and MeJA. *Plant cell Physiol.* **51,** 1721–1730 (2010).

44. Gonugunta, V. K., Srivastava, N., Puli, M. R. & Raghavendra, A. S. Nitric oxide production occurs after cytosolic alkalinization during stomatal closure induced by abscisic acid. *Plant. Cell Environ.* **31,** 1717–1724 (2008).

45. Ma, Y. *et al.* Regulators of PP2C phosphatase activity function as abscisic acid sensors. *Science (80-. ).* **324,** 1064 (2009).

46. Zhang, W., Qin, C., Zhao, J. & Wang, X. Phospholipase Dα1-derived phosphatidic acid interacts with ABI1 phosphatase 2C and regulates abscisic acid signaling. *Proc. Natl. Acad. Sci. U. S. A.* **101,** 9508–9513 (2004).

47. Castagnet, P. I. & Giusto, N. M. Effect of light and protein phosphorylation on photoreceptor rod outer segment acyltransferase activity. *Arch. Biochem. Biophys.* **403,** 83–91 (2002).

48. Meinhard, M. & Grill, E. Hydrogen peroxide is a regulator of ABI1, a protein phosphatase 2C from Arabidopsis. *FEBS Lett.* **508,** 443–446 (2001).

49. Miao, Y. *et al.* An Arabidopsis Glutathione Peroxidase Functions as Both a Redox Transducer and a Scavenger in Abscisic Acid and Drought Stress Responses. *Plant Cell Online* **18,** 2749–2766 (2006).

50. Meinhard, M., Rodriguez, P. L. & Grill, E. The sensitivity of ABI2 to hydrogen peroxide links the abscisic acid-response regulator to redox signalling. *Planta* **214,** 775–782 (2002).

51. Leube, M. P., Grill, E. & Amrhein, N. ABI1 of Arabidopsis is a protein serine/threonine phosphatase highly regulated by the proton and magnesium ion concentration 1. *FEBS Lett.* **424,** 100–104 (1998).

52. Yu, F. *et al.* FERONIA receptor kinase pathway suppresses abscisic acid signaling in Arabidopsis by activating ABI2 phosphatase. *Proc. Natl. Acad. Sci.* **109,** 14693–14698 (2012).

53. ronique Hugouvieux, V., Kwak, J. M. & Schroeder, J. I. An mRNA cap binding protein, ABH1, modulates early abscisic acid signal transduction in Arabidopsis. *Cell* **106,** 477–487 (2001).

54. Soon, F.-F. *et al.* Molecular mimicry regulates ABA signaling by SnRK2 kinases and PP2C phosphatases. *Science (80-. ).* **335,** 85–88 (2012).

55. Jones, J. A., Rawles, R. & Hannun, Y. A. Identification of a novel phosphatidic acid binding domain in protein phosphatase-1. *Biochemistry* **44,** 13235–13245 (2005).

56. Jammes, F. *et al.* MAP kinases MPK9 and MPK12 are preferentially expressed in guard cells and positively regulate ROS-mediated ABA signaling. *Proc. Natl. Acad. Sci.* **106,** 20520–20525 (2009).

57. Chen, Q., Olashaw, N. & Wu, J. Participation of Reactive Oxygen Species in the Lysophosphatidic Acid-stimulated Mitogen-activated Protein Kinase Kinase Activation Pathway. *J. Biol. Chem.* **270,** 28499–28502 (1995).

58. Mehlmer, N. Ca2+ dependent protein kinases in Arabidopsis thaliana. (2008).

59. Huang, S., Maher, V. M. & McCORMICK, J. J. Extracellular Ca2+ stimulates the activation of mitogen-activated protein kinase and cell growth in human fibroblasts. *Biochem. J* **310,** 881–885 (1995).

60. Leung, J. *et al.* Antagonistic interaction between MAP kinase and protein phosphatase 2C in stress recovery. *Plant Sci.* **171,** 596–606 (2006).

61. Harper, J. F. & Harmon, A. Plants, symbiosis and parasites: a calcium signalling connection. *Nat. Rev. Mol. cell Biol.* **6,** 555–566 (2005).

62. Geiger, D. *et al.* Guard cell anion channel SLAC1 is regulated by CDPK protein kinases with distinct Ca2+ affinities. *Proc. Natl. Acad. Sci.* **107,** 8023 (2010).

63. Sirichandra, C. *et al.* Phosphorylation of the Arabidopsis AtrbohF NADPH oxidase by OST1 protein kinase. *FEBS Lett.* **583,** 2982–2986 (2009).

64. Ogasawara, Y. *et al.* Synergistic Activation of the Arabidopsis NADPH Oxidase AtrbohD by Ca2+ and Phosphorylation. *J. Biol. Chem.* **283,** 8885–8892 (2008).

65. Saito, N. *et al.* Roles of RCN1, regulatory A subunit of protein phosphatase 2A, in methyl jasmonate signaling and signal crosstalk between methyl jasmonate and abscisic acid. *Plant cell Physiol.* **49,** 1396 (2008).

66. Zhang, W., Jeon, B. W. & Assmann, S. M. Heterotrimeric G-protein regulation of ROS signalling and calcium currents in Arabidopsis guard cells. *J. Exp. Bot.* **62,** 2371 (2011).

67. Kwak, J. M., Nguyen, V. & Schroeder, J. I. The Role of Reactive Oxygen Species in Hormonal Responses. *Plant Physiol.* **141,** 323–329 (2006).

68. Sagi, M. & Fluhr, R. Production of Reactive Oxygen Species by Plant NADPH Oxidases. *Plant Physiol.* **141,** 336–340 (2006).

69. del Rio, L. A. & Puppo, A. *Reactive oxygen species in plant signaling*. (Springer, 2009).

70. Zhang, L., Zhao, X., Wang, Y.-J. & Zhang, X. Crosstalk of Nitric Oxide with Ca< sup> 2+</sup> in Stomatal Movement in< i> Vicia faba</i> Guard Cells. *Acta Agron. Sin.* **35,** 1491–1499 (2009).

71. Labudda, M. & Azam, F. M. S. Glutathione-dependent responses of plants to drought: a review. *Acta Soc. Bot. Pol.* (2014).

72. Quintana-Cabrera, R. *et al.* γ-Glutamylcysteine detoxifies reactive oxygen species by acting as glutathione peroxidase-1 cofactor. *Nat Commun* **3,** 718 (2012).

73. Gutscher, M. *et al.* Proximity-based protein thiol oxidation by H2O2-scavenging peroxidases. *J. Biol. Chem.* **284,** 31532–31540 (2009).

74. Bright, J., Desikan, R., Hancock, J. T., Weir, I. S. & Neill, S. J. ABA‐induced NO generation and stomatal closure in Arabidopsis are dependent on H2O2 synthesis. *Plant J.* **45,** 113–122 (2006).

75. Lum, H. K., Butt, Y. K. C. & Lo, S. C. L. Hydrogen Peroxide Induces a Rapid Production of Nitric Oxide in Mung Bean (< i> Phaseolus aureus</i>). *Nitric oxide* **6,** 205–213 (2002).

76. Dubovskaya, L. V *et al.* cGMP-dependent ABA-induced stomatal closure in the ABA-insensitive Arabidopsis mutant abi1-1. *New Phytol.* **191,** 57–69 (2011).

77. Guo, F.-Q., Okamoto, M. & Crawford, N. M. Identification of a plant nitric oxide synthase gene involved in hormonal signaling. *Science (80-. ).* **302,** 100–103 (2003).

78. Wang, W.-H. *et al.* Calcium-sensing receptor regulates stomatal closure through hydrogen peroxide and nitric oxide in response to extracellular calcium in Arabidopsis. *J. Exp. Bot.* **63,** 177–190 (2012).

79. Durner, J. & Klessig, D. F. Nitric oxide as a signal in plants. *Curr. Opin. Plant Biol.* **2,** 369–374 (1999).

80. Leckie, C. P., McAinsh, M. R., Allen, G. J., Sanders, D. & Hetherington, A. M. Abscisic acid-induced stomatal closure mediated by cyclic ADP-ribose. *Proc. Natl. Acad. Sci. U. S. A.* **95,** 15837 (1998).

81. Bellamy, T. C., Wood, J., Goodwin, D. A. & Garthwaite, J. Rapid desensitization of the nitric oxide receptor, soluble guanylyl cyclase, underlies diversity of cellular cGMP responses. *Proc. Natl. Acad. Sci.* **97,** 2928–2933 (2000).

82. Moreau, M., Lindermayr, C., Durner, J. & Klessig, D. F. NO synthesis and signaling in plants–where do we stand? *Physiol. Plant.* **138,** 372–383 (2010).

83. Held, K. F. & Dostmann, W. R. Sub-nanomolar sensitivity of nitric oxide mediated regulation of cGMP and vasomotor reactivity in vascular smooth muscle. *Front. Pharmacol.* **3,** (2012).

84. Geiger, D. *et al.* Activity of guard cell anion channel SLAC1 is controlled by drought-stress signaling kinase-phosphatase pair. *Proc. Natl. Acad. Sci.*  **106,** 21425–21430 (2009).

85. Pei, Z.-M., Ghassemian, M., Kwak, C. M., McCourt, P. & Schroeder, J. I. Role of Farnesyltransferase in ABA Regulation of Guard Cell Anion Channels and Plant Water Loss. *Science (80-. ).* **282,** 287–290 (1998).

86. Suh, S. J. *et al.* The ATP Binding Cassette Transporter AtMRP5 Modulates Anion and Calcium Channel Activities in Arabidopsis Guard Cells. *J. Biol. Chem.* **282,** 1916–1924 (2007).

87. Raschke, K. Alternation of the slow with the quick anion conductance in whole guard cells effected by external malate. *Planta* **217,** 651–657 (2003).

88. Beguerisse-Díaz, M., Hernández-Gómez, M. C., Lizzul, A. M., Barahona, M. & Desikan, R. Compound stress response in stomatal closure: a mathematical model of ABA and ethylene interaction in guard cells. *BMC Syst. Biol.* **6,** 146 (2012).

89. Köhler, B., Hills, A. & Blatt, M. R. Control of guard cell ion channels by hydrogen peroxide and abscisic acid indicates their action through alternate signaling pathways. *Plant Physiol.* **131,** 385–388 (2003).

90. Schroeder, J. I. Quantitative analysis of outward rectifying K+ channel currents in guard cell protoplasts fromVicia faba. *J. Membr. Biol.* **107,** 229–235 (1989).

91. Imes, D. *et al.* Open stomata 1 (OST1) kinase controls R–type anion channel QUAC1 in Arabidopsis guard cells. *Plant J.* **74,** 372–382 (2013).

92. Hedrich, R., Busch, H. & Raschke, K. Ca2+ and nucleotide dependent regulation of voltage dependent anion channels in the plasma membrane of guard cells. *EMBO J.* **9,** 3889 (1990).

93. Schmidt, C. & Schroeder, J. I. Anion Selectivity of Slow Anion Channels in the Plasma Membrane of Guard Cells (Large Nitrate Permeability). *Plant Physiol.* **106,** 383–391 (1994).

94. Meyer, S. *et al.* Malate transport by the vacuolar AtALMT6 channel in guard cells is subject to multiple regulation. *Plant J.* **67,** 247–257 (2011).

95. Li, J., Lee, Y.-R. J. & Assmann, S. M. Guard cells possess a calcium-dependent protein kinase that phosphorylates the KAT1 potassium channel. *Plant Physiol.* **116,** 785–795 (1998).

96. Berkowitz, G., Zhang, X., Mercier, R., Leng, Q. & Lawton, M. Co-expression of calcium-dependent protein kinase with the inward rectified guard cell K+ channel KAT1 alters current parameters in Xenopus laevis oocytes. *Plant cell Physiol.* **41,** 785–790 (2000).

97. Sato, A. *et al.* Threonine at position 306 of the KAT1 potassium channel is essential for channel activity and is a target site for ABA-activated SnRK2/OST1/SnRK2. 6 protein kinase. *Biochem. J* **424,** 439–448 (2009).

98. Sutter, J.-U. *et al.* Abscisic Acid Triggers the Endocytosis of the< i> Arabidopsis</i> KAT1 K< sup>+</sup> Channel and Its Recycling to the Plasma Membrane. *Curr. Biol.* **17,** 1396–1402 (2007).

99. Latz, A. *et al.* TPK1, a Ca2+‐regulated Arabidopsis vacuole two‐pore K+ channel is activated by 14‐3‐3 proteins. *Plant J.* **52,** 449–459 (2007).

100. Gobert, A., Isayenkov, S., Voelker, C., Czempinski, K. & Maathuis, F. J. M. The two-pore channel TPK1 gene encodes the vacuolar K+ conductance and plays a role in K+ homeostasis. *Proc. Natl. Acad. Sci.* **104,** 10726–10731 (2007).

101. Becker, D. *et al.* AtTPK4, an Arabidopsis tandem-pore K+ channel, poised to control the pollen membrane voltage in a pH-and Ca2+-dependent manner. *Proc. Natl. Acad. Sci. U. S. A.* **101,** 15621–15626 (2004).

102. Paulus, J. K., Schlieper, D. & Groth, G. Greater efficiency of photosynthetic carbon fixation due to single amino-acid substitution. *Nat. Commun.* **4,** 1518 (2013).

103. Outlaw, W. H. & Zhang, S. Single‐cell dissection and microdroplet chemistry. *J. Exp. Bot.* **52,** 605–614 (2001).

104. Takemiya, A., Yamauchi, S., Yano, T., Ariyoshi, C. & Shimazaki, K. Identification of a Regulatory Subunit of Protein Phosphatase 1 Which Mediates Blue Light Signaling for Stomatal Opening. *Plant cell Physiol.* **54,** 24–35 (2013).

105. Hayashi, Y. *et al.* Biochemical characterization of in vitro phosphorylation and dephosphorylation of the plasma membrane H+-ATPase. *Plant cell Physiol.* **51,** 1186–1196 (2010).

106. Kinoshita, T., Nishimura, M. & Shimazaki, K. Cytosolic Concentration of Ca2+ Regulates the Plasma Membrane H+-ATPase in Guard Cells of Fava Bean. *Plant Cell Online* **7,** 1333–1342 (1995).

107. Morsomme, P. & Boutry, M. The plant plasma membrane H< sup>+</sup>-ATPase: structure, function and regulation. *Biochim. Biophys. Acta (BBA)-Biomembranes* **1465,** 1–16 (2000).

108. Ma, T., Liu, Q., Li, Z. & Zhang, X. Tonoplast H< sup>+</sup>-ATPase in response to salt stress in< i> Populus euphratica</i> cell suspensions. *Plant Sci.* **163,** 499–505 (2002).

109. Schwanninger, M., Kant, M., Schuurink, R., Mauch, F. & Buchala, A. The PP2C-Type Phosphatase AP2C1 , Which Negatively Regulates MPK4 and MPK6 , Modulates Innate Immunity , Jasmonic Acid , and Ethylene Levels in Arabidopsis. **19,** 2213–2224 (2007).

110. Cosgrove, D. J. & Hedrich, R. Stretch-activated chloride, potassium, and calcium channels coexisting in plasma membranes of guard cells of Vicia faba L. *Planta* **186,** 143–153 (1991).

111. Siegel, R. S. *et al.* Calcium elevation-dependent and attenuated resting calcium-dependent abscisic acid induction of stomatal closure and abscisic acid-induced enhancement of calcium sensitivities of S-type anion and inward-rectifying K+ channels in Arabidopsis guard cells. *Plant J.* **59,** 207–220 (2009).

112. Bose, J., Pottosin, I. I., Shabala, S. S., Palmgren, M. G. & Shabala, S. Calcium efflux systems in stress signaling and adaptation in plants. *Front. Plant Sci.* **2,** (2011).

113. Zhai, J. *et al.* Ca2+/H+ exchange in the plasma membrane of Arabidopsis thaliana leaves. *Acta Physiol. Plant.* **34,** 1–13 (2012).

114. Luoni, L., Bonza, M. C. & De Michelis, M. I. Calmodulin/Ca2+‐ATPase interaction at the Arabidopsis thaliana plasma membrane is dependent on calmodulin isoform showing isoform‐specific Ca2+ dependencies. *Physiol. Plant.* **126,** 175–186 (2006).

115. Teruel, M. N., Chen, W., Persechini, A. & Meyer, T. Differential codes for free Ca< sup> 2+</sup>–calmodulin signals in nucleus and cytosol. *Curr. Biol.* **10,** 86–94 (2000).

116. Hugouvieux, V. *et al.* Localization, ion channel regulation, and genetic interactions during abscisic acid signaling of the nuclear mRNA cap-binding protein, ABH1. *Plant Physiol.* **130,** 1276–1287 (2002).

117. Hedrich, R. Ion Channels in Plants. *Physiol. Rev.* **92,** 1777–1811 (2012).

118. Stange, A., Hedrich, R. & Roelfsema, M. R. G. Ca2+-dependent activation of guard cell anion channels, triggered by hyperpolarization, is promoted by prolonged depolarization. *Plant J.* **62,** 265–276 (2010).

119. Hashimoto, K. *et al.* Phosphorylation of Calcineurin B-like (CBL) Calcium Sensor Proteins by Their CBL-interacting Protein Kinases (CIPKs) Is Required for Full Activity of CBL-CIPK Complexes toward Their Target Proteins. *J. Biol. Chem.* **287,** 7956–7968 (2012).

120. Li, L., Kim, B.-G., Cheong, Y. H., Pandey, G. K. & Luan, S. A Ca2+ signaling pathway regulates a K+ channel for low-K response in Arabidopsis. *Proc. Natl. Acad. Sci.* **103,** 12625–12630 (2006).

121. Cheng, N.-H., Liu, J.-Z., Nelson, R. S. & Hirschi, K. D. Characterization of CXIP4, a novel< i> Arabidopsis</i> protein that activates the H< sup>+</sup>/Ca< sup> 2+</sup> antiporter, CAX1. *FEBS Lett.* **559,** 99–106 (2004).

122. Zhao, Y. *et al.* The Plant-Specific Actin Binding Protein SCAB1 Stabilizes Actin Filaments and Regulates Stomatal Movement in Arabidopsis. *Plant Cell Online* **23,** 2314–2330 (2011).

123. WANG, X. X., GAO, X. & WANG, X. X. Stochastic dynamics of actin filaments in guard cells regulating chloroplast localization during stomatal movement. *Plant. Cell Environ.* **34,** 1248–1257 (2011).

124. Zhang, W., Zhao, Y., Guo, Y. & Ye, K. Plant actin-binding protein SCAB1 is dimeric actin cross-linker with atypical pleckstrin homology domain. *J. Biol. Chem.* **287,** 11981–11990 (2012).

125. Zhang, W., Fan, L.-M. & Wu, W.-H. Osmo-sensitive and stretch-activated calcium-permeable channels in Vicia faba guard cells are regulated by actin dynamics. *Plant Physiol.* **143,** 1140–1151 (2007).

126. Kudla, J., Xu, Q., Harter, K., Gruissem, W. & Luan, S. Genes for calcineurin B-like proteins in Arabidopsis are differentially regulated by stress signals. *Proc. Natl. Acad. Sci.* **96,** 4718–4723 (1999).

127. Tominaga, M., Harada, A., Kinoshita, T. & Shimazaki, K. Biochemical Characterization of Calcineurin B-Like-Interacting Protein Kinase in Vicia Guard Cells. *Plant cell Physiol.* **51,** 408–421 (2010).

128. Weinl, S. & Kudla, J. The CBL–CIPK Ca2+-decoding signaling network: function and perspectives. *New Phytol.* **184,** 517–528 (2009).

129. Takemiya, A. & Shimazaki, K. Phosphatidic acid inhibits blue light-induced stomatal opening via inhibition of protein phosphatase 1. *Plant Physiol.* **153,** 1555–1562 (2010).

130. Yokota, E. *et al.* Plant villin, lily P-135-ABP, possesses G-actin binding activity and accelerates the polymerization and depolymerization of actin in a Ca2+-sensitive manner. *Plant cell Physiol.* **46,** 1690–1703 (2005).

131. Choi, Y., Lee, Y., Jeon, B. W., Staiger, C. J. & Lee, Y. Phosphatidylinositol 3- and 4-phosphate modulate actin filament reorganization in guard cells of day flower. *Plant. Cell Environ.* **31,** 366–377 (2008).

132. Li, X. I. N. *et al.* ARP2/3 complex-mediated actin dynamics is required for hydrogen peroxide-induced stomatal closure in Arabidopsis. *Plant. Cell Environ.* n/a-n/a (2014). doi:10.1111/pce.12259

133. LeClaire Iii, L. L. *et al.* Phosphorylation of the Arp2/3 complex is necessary to nucleate actin filaments. *J. Cell Biol.* **182,** 647–654 (2008).

134. Lemichez, E. *et al.* Inactivation of AtRac1 by abscisic acid is essential for stomatal closure. *Sci. STKE* **15,** 1808 (2001).

135. DalleDonne, I., Milzani, A. & Colombo, R. H2O2-treated actin: assembly and polymer interactions with cross-linking proteins. *Biophys. J.* **69,** 2710–2719 (1995).

136. Hayes, M. J., Shao, D., Bailly, M. & Moss, S. E. Regulation of actin dynamics by annexin 2. *EMBO J.* **25,** 1816–1826 (2006).

137. Ma, B. *et al.* Arabidopsis vacuolar H+-ATPase (V-ATPase) B subunits are involved in actin cytoskeleton remodeling via binding to, bundling, and stabilizing F-actin. *J. Biol. Chem.* **287,** 19008–19017 (2012).

138. Rozelle, A. L. *et al.* Phosphatidylinositol 4, 5-bisphosphate induces actin-based movement of raft-enriched vesicles through WASP-Arp2/3. *Curr. Biol.* **10,** 311–320 (2000).

139. Higgs, H. N. & Pollard, T. D. Activation by Cdc42 and PIP2 of Wiskott-Aldrich syndrome protein (WASp) stimulates actin nucleation by Arp2/3 complex. *J. Cell Biol.* **150,** 1311–1320 (2000).

140. Gonugunta, V. K., Srivastava, N. & Raghavendra, A. S. Cytosolic alkalinization is a common and early messenger preceding the production of ROS and NO during stomatal closure by variable signals, including abscisic acid, methyl jasmonate and chitosan. *Plant Signal. Behav.* **4,** 561–564 (2009).

141. Trouverie, J. *et al.* Anion channel activation and proton pumping inhibition involved in the plasma membrane depolarization induced by ABA in Arabidopsis thaliana suspension cells are both ROS dependent. *Plant Cell Physiol.* **49,** 1495–1507 (2008).

142. Lipscombe, D. in *eLS* (John Wiley & Sons, Ltd, 2001). doi:10.1038/npg.els.0004070

143. Demaurex, N. Functions of proton channels in phagocytes. *Wiley Interdiscip. Rev. Membr. Transp. Signal.* **1,** 3–15 (2012).

144. Vahisalu, T. *et al.* SLAC1 is required for plant guard cell S-type anion channel function in stomatal signalling. *Nature* **452,** 487–491 (2008).

145. Linder, B. & Raschke, K. A slow anion channel in guard cells, activating at large hyperpolarization, may be principal for stomatal closing. *FEBS Lett.* **313,** 27–30 (1992).

146. Meyer, S. *et al.* AtALMT12 represents an R‐type anion channel required for stomatal movement in Arabidopsis guard cells. *Plant J.* **63,** 1054–1062 (2010).

147. Kolb, H. A., Marten, I. & Hedrich, R. Hodgkin-Huxley analysis of a GCAC1 anion channel in the plasma membrane of guard cells. *J. Membr. Biol.* **146,** 273–282 (1995).

148. Eun, S. O. & Lee, Y. Actin Filaments of Guard Cells Are Reorganized in Response to Light and Abscisic Acid. *Plant Physiol.* **115,** 1491–1498 (1997).

149. Du, Z., Aghoram, K. & Outlaw, W. H. In VivoPhosphorylation of Phosphoenolpyruvate Carboxylase in Guard Cells ofVicia fabaL. Is Enhanced by Fusicoccin and Suppressed by Abscisic Acid. *Arch. Biochem. Biophys.* **337,** 345–350 (1997).

150. Vahisalu, T. *et al.* SLAC1 is required for plant guard cell S-type anion channel function in stomatal signalling. *Nature* **452,** 487 (2008).

151. Levchenko, V., Konrad, K. R., Dietrich, P., Roelfsema, M. R. G. & Hedrich, R. Cytosolic abscisic acid activates guard cell anion channels without preceding Ca2+ signals. *Proc. Natl. Acad. Sci. U. S. A.* **102,** 4203–4208 (2005).

152. Suhita, D., Raghavendra, A. S., Kwak, J. M. & Vavasseur, A. Cytoplasmic Alkalization Precedes Reactive Oxygen Species Production during Methyl Jasmonate- and Abscisic Acid-Induced Stomatal Closure. *Plant Physiol.* **134,** 1536–1545 (2004).

153. Puli, M. R. & Raghavendra, A. S. Pyrabactin, an ABA agonist, induced stomatal closure and changes in signalling components of guard cells in abaxial epidermis of Pisum sativum. *J. Exp. Bot.* (2011). doi:10.1093/jxb/err364

154. Zhang, Y. *et al.* Phospholipase Dα1 and Phosphatidic Acid Regulate NADPH Oxidase Activity and Production of Reactive Oxygen Species in ABA-Mediated Stomatal Closure in Arabidopsis. *Plant Cell Online* **21,** 2357–2377 (2009).

155. Allen, G. J., Kuchitsu, K., Chu, S. P., Murata, Y. & Schroeder, J. I. Arabidopsis abi1-1 and abi2-1 phosphatase mutations reduce abscisic acid-induced cytoplasmic calcium rises in guard cells. *Plant Cell* **11,** 1785–1798 (1999).

156. Allen, G. J., Murata, Y., Chu, S. P., Nafisi, M. & Schroeder, J. I. Hypersensitivity of Abscisic Acid–Induced Cytosolic Calcium Increases in the Arabidopsis Farnesyltransferase Mutant era1-2. *Plant Cell*  **14,** 1649–1662 (2002).

157. Szostkiewicz, I. *et al.* Closely related receptor complexes differ in their ABA selectivity and sensitivity. *Plant J.* **61,** 25–35 (2010).

158. Becker, D. *et al.* Regulation of the ABA-sensitive< i> Arabidopsis</i> potassium channel gene< i> GORK</i> in response to water stress. *FEBS Lett.* **554,** 119–126 (2003).

159. Guo, L. *et al.* Inter-relationship between sphingosine kinase and phospholipase D in signaling Arabidopsis response to abscisic acid. (2012).

160. Ng, C. K. Y. & Coursol, S. New Insights into Phospholipase D and Sphingosine Kinase Activation in Arabidopsis. *Front. Physiol.* **3,** (2012).

161. Kim, T. Y., Kim, H. U. & Lee, S. Y. Data integration and analysis of biological networks. *Curr. Opin. Biotechnol.* **21,** 78–84 (2010).

162. Cousson, A. Involvement of phospholipase C-independent calcium-mediated abscisic acid signalling during Arabidopsis response to drought. *Biol. Plant.* **53,** 53–62 (2009).

163. Assmann, S. M. D6. Abscisic Acid Signal Transduction in Stomatal Responses. *Plant Horm. Biosynthesis, Signal Transduction, Action!* 391 (2010).

164. Fan, L. M., Zhao, Z. & Assmann, S. M. Guard cells: a dynamic signaling model. *Curr. Opin. Plant Biol.* **7,** 537–546 (2004).

165. Kwak, J. M. *et al.* NADPH oxidase AtrbohD and AtrbohF genes function in ROS-dependent ABA signaling in Arabidopsis. *EMBO J.* **22,** 2623–2633 (2003).

166. Park, K. Y. *et al.* A role for phosphatidylinositol 3-phosphate in abscisic acid-induced reactive oxygen species generation in guard cells. *Plant Physiol.* **132,** 92–98 (2003).

167. Kwak, J. M. *et al.* Disruption of a guard cell–expressed protein phosphatase 2A regulatory subunit, RCN1, confers abscisic acid insensitivity in Arabidopsis. *Plant Cell Online* **14,** 2849–2861 (2002).

168. Xing, Y., Jia, W. & Zhang, J. AtMKK1 mediates ABA-induced CAT1 expression and H2O2 production via AtMPK6-coupled signaling in Arabidopsis. *Plant J.* **54,** 440–451 (2008).

169. Kuromori, T., Sugimoto, E. & Shinozaki, K. Arabidopsis mutants of AtABCG22, an ABC transporter gene, increase water transpiration and drought susceptibility. *Plant J.* **67,** 885–894 (2011).

170. Merlot, S. *et al.* Constitutive activation of a plasma membrane H&plus;-ATPase prevents abscisic acid-mediated stomatal closure. *EMBO J.* **26,** 3216–3226 (2007).

171. Luan, S. *Coding and Decoding of Calcium Signals in Plants*. **10,** (Springer, 2011).

172. Jiang, K. Identifying novel stomatal signalling components in Arabidopis thaliana. (University of Bristol, 2009).

173. Jiang, K. *et al.* The ARP2/3 Complex Mediates Guard Cell Actin Reorganization and Stomatal Movement in Arabidopsis. *Plant Cell Online* (2012). doi:10.1105/tpc.112.096263
